# Targeting Colorectal Cancer Stem Cells Through Inhibition of the Fibroblast Growth Factor Receptor 4 Pathway with a Novel Antibody

**DOI:** 10.3390/cancers18030418

**Published:** 2026-01-28

**Authors:** Gessica Filocamo, Mariachiara Buccarelli, Armin Lahm, Mirko Brunetti, Chantal Paolini, Gabriele De Luca, Michele Signore, Giorgia Castellani, Alessandra Boe, Romina Alfonsi, Mauro Biffoni, Ruggero De Maria, Lucia Ricci-Vitiani, Christian Steinkühler, Paola Gallinari

**Affiliations:** 1 Exiris s.r.l., Tecnopolo Castel Romano, 00128 Rome, Italy; mirko_brunetti@exiris.it (M.B.); christian_steinkuhler@exiris.it (C.S.); paola_gallinari@exiris.it (P.G.); 2 Department of Oncology and Molecular Medicine, Istituto Superiore di Sanità, 00161 Rome, Italy; mariachiara.buccarelli@iss.it (M.B.); gabriele.deluca@iss.it (G.D.L.); giorgia.castellani@iss.it (G.C.); mauro.biffoni@iss.it (M.B.); lucia.riccivitiani@iss.it (L.R.-V.); 3 Bioinformatics Project Support, 00153 Rome, Italy; mo1339@mclink.it; 4 IRBM S.p.A., 00071 Pomezia, Italy; c.paolini@irbm.it; 5 RPPA Unit, Proteomics Area, Core Facilities, Istituto Superiore di Sanità, 00161 Rome, Italy; michele.signore@iss.it; 6 Cytometry Unit, Core Facilities, Istituto Superiore di Sanità, 00161 Rome, Italy; alessandra.boe@iss.it; 7 National Centre for Control and Evaluation of Medicines, Istituto Superiore di Sanità, 00161 Rome, Italy; romina.alfonsi@iss.it; 8 Dipartimento di Medicina e Chirurgia Traslazionale, Università Cattolica del Sacro Cuore, 00168 Rome, Italy; ruggero.demaria@unicatt.it; 9 Fondazione Policlinico Universitario A. Gemelli IRCCS, 00168 Rome, Italy; 10 Research and Development, Italfarmaco Group, 20092 Cinisello Balsamo, Italy

**Keywords:** targeted therapy, cancer stem cells, colon cancer, FGFR4, EMT, monoclonal antibodies, microarray dataset, differentially expressed genes

## Abstract

Background/Objectives: The progression and dissemination of CRC are heavily influenced by a subpopulation of tumor cells known as CSCs. This study aimed to identify novel protein membrane antigens expressed by colorectal CSCs and the consequent development of targeted therapies based on monoclonal antibodies directed against the identified antigens. Methods: Integrated bioinformatics analyses were conducted using proprietary CSC gene expression profiles and public colon gene expression databases, leading to the identification of five plasma membrane proteins enriched in CSCs. Genetic immunization in rats was employed to generate monoclonal antibodies (mAbs) targeting these antigens. FGFR4 was prioritized due to its overexpression in colorectal tumors. Its function was characterized in vitro and in vivo through assays evaluating proliferation, colony formation, migration, and tumorigenicity. The anti-FGFR4 antibody 3B6 was selected based on its affinity and ability to inhibit FGFR4 signaling in CSCs. Its therapeutic potential was further assessed in xenograft models, and alterations in downstream signaling were analyzed via Western blot. Results: FGFR4 emerged as a key regulator of CRC CSC proliferation, migration, and tumorigenic capacity. The 3B6 antibody, a high-affinity FGFR4 binder, demonstrated robust in vitro inhibition of CSC features and significant antitumor effects in patient-derived xenograft models. Western blot analysis confirmed the modulation of FGFR4-driven signaling pathways, particularly those involved in epithelial–mesenchymal transition (EMT). Conclusions: This study successfully identified several CSC-selective membrane antigens that can become therapeutic targets in CRC. Among them, we focused on FGFR4 as a promising target and developed the anti-FGFR4 3B6 monoclonal antibody which offers potential for both diagnostic and therapeutic applications.

## Simple Summary

Metastatic colorectal cancer (CRC) is a severe disease, often resistant to current treatments due to a subpopulation of resilient tumor cells known as cancer stem cells (CSCs). The aim of this study was to find a new way to target these difficult-to-treat cells. Through genetic analysis, we discovered fibroblast growth factor receptor 4 (FGFR4) controlling the survival and growth of these cells. We then created a specific antibody to bind to this protein and inhibit tumor growth. The results demonstrate that this antibody could represent a novel and promising therapeutic approach for patients with metastatic CRC who currently lack effective treatment options.

## 1. Introduction

Metastatic solid tumors remain largely incurable despite remarkable advances in cancer research, with nearly 10 million cancer-related deaths reported worldwide in 2022 alone [1]. CRC exemplifies this challenge, ranking as the third most common cancer globally and the second leading cause of cancer-related mortality, with 1.9 million new cases and 900,000 deaths in 2022 [2]. The burden is projected to increase by 60% by 2040, underscoring the urgent need for innovative therapeutic approaches. Between 15% and 30% of patients present with metastases at diagnosis, and up to half of those initially diagnosed with localized disease will eventually develop metastases [3]. As a result, survival rates for advanced CRC continue to be unsatisfactory.

Current therapeutic strategies for metastatic CRC face significant limitations. First-line treatments typically involve cytotoxic chemotherapy combined with targeted biologics including bevacizumab, aflibercept, cetuximab, and panitumumab. However, anti-EGFR therapies benefit only 45% of patients lacking RAS mutations [4], while anti-PD-1 checkpoint inhibitors are restricted to the minority of cases with defective DNA mismatch repair or microsatellite instability [5]. These constraints highlight the critical need for novel, biomarker-driven therapeutic strategies.

A major contributor to therapeutic resistance in metastatic CRC [6] is a rare subpopulation of tumor cells known as CSCs [7]. Initially characterized by static traits such as fixed surface markers and asymmetric division patterns [8,9], CSCs are now recognized as highly dynamic entities capable of self-renewal, multilineage differentiation, and profound adaptation to environmental stressors [10]. Crucially, even differentiated tumor cells can revert to a stem-like state under specific conditions [11,12,13], enabling CSCs to withstand conventional therapies, drive recurrence, and facilitate long-term metastatic latency [14,15,16]. Some of the present study’s authors were among the first to isolate CSCs from CRC specimens, establishing standardized protocols for in vitro and in vivo amplification showing that colorectal CSCs can form xenografts in immunocompromised mice that closely resemble the original human tumors both genetically and histologically [17,18,19]. Despite their pivotal role in tumor progression, no clinically approved therapies currently exist that selectively target these cells [10,20,21].

FGFR4 a member of the FGF receptor tyrosine kinase family [22,23,24], has emerged as a promising oncogenic driver across various malignancies. FGFR4 and its ligand FGF19 are frequently overexpressed in solid tumors, including hepatocellular carcinoma (HCC), rhabdomyosarcoma, and CRC [25,26,27,28,29,30,31,32,33,34]. Overexpression and specific polymorphisms (e.g., Gly388Arg) correlate with poor clinical outcomes [35,36,37,38,39,40,41,42,43,44], while preclinical studies link FGFR4 to enhanced proliferation, survival, multidrug and radiotherapy resistance, and metastasis [45,46,47,48,49,50,51,52]—all hallmarks relevant to CRC pathophysiology.

Accumulating evidence identifies FGFR4 as a potential therapeutic target, particularly in HCC, where FGFR4 inhibitors are already in clinical testing [53]. Several anti-FGFR4 monoclonal antibodies have shown antitumor activity in preclinical HCC and other FGFR4-driven malignancy models [54,55,56,57], with only U3-1784 (Daiichi Sankyo, Tokyo, Japan) advancing to a Phase I trial in advanced solid tumors and liver cancer (NCT02690350), later discontinued for strategic reasons. Next-generation selective FGFR4 tyrosine kinase inhibitors—including fisogatinib (BLU-554), roblitinib (FGF401), and H3B-6527—have shown efficacy in HCC models and are in Phase I/II trials for FGF19- or FGFR4/KLB-positive tumors [58]; however, resistance mutations have emerged, as seen with other receptor tyrosine kinase inhibitors [59]. Importantly, no FGFR4-directed therapeutics have been shown to selectively target colorectal CSCs, representing a significant therapeutic gap. The controversy surrounding CSC plasticity and their role in drug resistance further complicates therapeutic development, with ongoing debates about whether targeting these cells can effectively prevent recurrence and metastasis. Thus, a monoclonal antibody that could impair growth or induce death in this cell population may represent a novel and promising therapeutic approach for patients with metastatic, FGFR4-positive CRC—a group currently lacking effective targeted options.

Through integrated bioinformatic analyses of colorectal CSC gene expression datasets, we identified several novel surface antigens enriched in colorectal CSCs, with FGFR4 ranking among the top five receptors. Our findings demonstrate that FGFR4 is specifically overexpressed in metastatic CRC, particularly in microsatellite stable (MSS) tumors, and controls crucial CSC functions including migration, survival, proliferation, and in vivo tumorigenicity. We subsequently generated and validated the monoclonal antibody 3B6, a subnanomolar and highly specific FGFR4 binder that demonstrates significant efficacy against colorectal CSCs in both in vitro and in vivo models.

This work establishes FGFR4 as a critical regulator of the colorectal CSC population responsible for treatment resistance and tumor relapse. Our novel anti-FGFR4 monoclonal antibody represents a promising therapeutic strategy for patients with metastatic, FGFR4-positive CRC—a population currently lacking effective targeted treatment options. These findings provide a foundation for developing CSC-directed therapies that could address the fundamental challenge of therapeutic resistance in CRC.

## 2. Materials and Methods

### 2.1. Bioinformatic Analysis

Total RNA was extracted from eight colorectal CSC lines that were either propagated under spherogenic conditions to preserve the stem phenotype or let progress into a differentiated phenotype by exposing them to 10% serum [17]. Purified RNA was assessed for integrity using the Bioanalyzer (Agilent Technologies, Santa Clara, CA, USA), labeled using the Affymetrix Whole-Transcript protocol and hybridized to the GeneChip Human Gene 1.0 ST array (Affymetrix, Santa Clara, CA, USA) following the manufacturer’s protocol. Gene expression data were then normalized using the robust multichip average (RMA) algorithm [60] and differentially expressed genes (DEGs) between the stem-phenotype CSCs (sCSCs) and the differentiated CSCs (dCSCs) were determined using RankProd [61] implemented in R 4.3.2 in Bioconductor (https://www.bioconductor.org/; https://www.r-project.org/) [62] applying a false positive prediction (pfp) cut-off of 0.1. Identification of DEGs suitable as target genes accessible to antibody therapy (integral membrane or glycosylphosphatidylinositol (GPI)-linked membrane-bound proteins) were identified using annotation available from the UNIPROT (https://www.uniprot.org/) database and data in the literature [63]. Gene expression data from Affymetrix HuGene arrays or arrays with probe sets overlapping with the HuGene Array (U133plus 2.0 arrays; 17,868/transcript from 17,090 genes; U133PlusVsHuGene_BestMatch.csv) from normal colonic mucosa and colon tumor samples were obtained from datasets in the NCBI GEO database (Figure S1) and normalized using Global Rank-Invariant Set Normalization (GRSN) [64]. Possible batch effects, due to the different array platforms utilized, were compensated for by ComBat [65]. ComBat batches were defined by the array platform and tissue types (sCSC, dCSC, normal colon, micro-dissected normal colon, micro-dissected normal colon crypt, colon tumor, LCM colon tumor, micro-dissected colon tumor, micro-dissected colon tumor crypts, colon cancer cell lines) represented the ComBat Covariate1 variable. In the combined dataset the large group of normal and tumor colon samples showed a relatively large heterogeneity and was reduced to a more focused representative subset by selecting only those samples falling within one median absolute deviation (MAD) from the medoid sample. This combined dataset comprising 507 samples was then examined with RankProd to estimate the fold-change gene expression difference between the sCSC group and the individual tissue classes (Table S1, “Combact” sheet). Further prioritization and reduction in the target gene list was performed with information from “BioGPS” (http://biogps.org/), “Body Atlas” gene expression data (NCBI GEO GSE14938), Uniprot annotation (https://www.uniprot.org/), the NCBI Gene database (https://www.ncbi.nlm.nih.gov/gene/ accessed on 13 February 2016), and scientific literature through PubMed (https://pubmed.ncbi.nlm.nih.gov/).

Analysis of the FGFR4 probeset results in the GEO dataset GSE41258 was performed by calculating mean values of normal mucosa samples versus CRC primary tumor samples vs. CRC metastatic samples, and differences were assessed using one-way analysis of variance (ANOVA). As the ANOVA indicated a statistically significant effect, post hoc pairwise comparisons were performed using Tukey’s honestly significant difference (HSD) test to adjust for multiple testing. A *p*-value < 0.05 was considered statistically significant.

### 2.2. Cell Culture

Patient-derived colorectal CSCs were provided by Dr. Ricci-Vitiani [17] as lines isolated from surgical samples subjected to mechanical and enzymatic dissociation using type II collagenase (Gibco Invitrogen by Thermo Fisher Scientific, Walthman, MA, USA). The resulting single cell suspension was cultured as spheroids in a defined serum-free stem cell medium supplemented with growth factors (20 ng/mL of human recombinant epidermal growth factor, EGF, and 10 ng/mL of human recombinant basic fibroblast growth factor, bFGF), as previously described [17]. To validate colorectal CSC lines, Short Tandem Repeat (STR) DNA fingerprinting was performed, as previously described [66]. Caco-2, DLD-1, HCT116, HCT15, HT-29, LoVo, SNU-C2B, SW48, SW480, SW620, WiDr, Hep3B, HuH7, HepG2, and MDA-MD 453 cells (ATCC, Manassas, VA, USA) were grown in the specific culture medium recommended by the manufacturer for each cell line. Cells were maintained in a humidifed incubator at 37 °C with 5% CO_2_. All cell lines were tested and negative for mycoplasma (N-GARDE Mycoplasma PCR reagent set, Euroclone, Milan, Italy).

### 2.3. FGFR4 -Based Cell Sorting

Colorectal CSC line #85 was sorted via flow cytometry to isolate FGFR4_High_ and FGFR4_Low_ subpopulations, based on a bimodal binding profile using the proprietary anti-FGFR4 antibody BFG-5F7. After enzymatic dissociation and cell counting, 7 × 10^6^ cells were incubated for 1 h with BFG-5F7, followed by a goat anti-rat IgG (gamma chain specific) PE-conjugated secondary antibody (Thermo Fisher Scientific). Cells were washed, stained with 7-AAD to assess viability, and sorted on a FACS Aria III (BD Biosciences, San José, CA, USA). A sample stained only with the secondary antibody was used as a negative control. Post-sorting, purity of the populations was confirmed by FACS. FGFR4_High_ and FGFR4_Low_ subpopulations, along with the parental “mock-sorted” cells, were subjected to various in vitro and in vivo assays as detailed in Section 3.

### 2.4. Cell Proliferation Assays

For FGFR4_High_ and FGFR4_Low_ cells, 2 × 10^3^ cells were seeded in triplicate in a 96-well plate and cultured for 14 days. Cell proliferation was monitored every two days using the CellTiter-Blue™ assay, measuring fluorescence intensity with a Wallac Victor 2™ microplate reader (Perkin Elmer, Walthman, MA, USA). Values were normalized to day 0. For compound/antibody-treated cells, 1 × 10^3^ cells were seeded in 24-well plates with spherogenic medium. Antibodies (1.3 μM) and BLU9931 (0.5 μM) were added, and cell viability was measured after 8 days using the CellTiter-Glo™ assay, with luminescence intensity assessed by a Victor Nivo™ reader (Perkin Elmer). Values were normalized to controls.

### 2.5. Clonogenicity Assay

For FGFR4_High_ and FGFR4_Low_ sorted subpopulations, 500 µL of a 0.9% agarose solution was dispensed into each well of a 24-well plate to solidify. A top layer of 0.6% agarose and cell suspension (1.3 × 10^4^ cells/well) was added, followed by culture medium. Colony formation was monitored over 12 days, and colonies with ≥30 cells were counted. For antibody/compound-treated Huh7 cells, 300 cells/well were incubated with antibodies (50–100 µg/mL) or BLU9931 (0.3–5 µM) for 10 days, and colonies were stained with Crystal Violet. For CSC spheroid cells, a similar agarose-based method was used, with antibodies or BLU9931 added weekly, and colonies were stained and counted after 10–11 days.

### 2.6. Transwell Migration Assay

In the transwell migration assay, 3 × 10^3^ cells were seeded in the upper chamber of Corning^®^ FluoroBlock 96-well microplates (Corning Life Science, Tewksbury, MA, USA) in quadruplicate, with 200 µL of complete growth medium in the lower chamber. After a 48 h incubation at 37 °C, 5% CO_2_, cells were stained with 4 µM Calcein AM fluorescent dye for 30 min at room temperature. Fluorescence from viable cells migrating through the membrane was measured at 485/535 nm (Ex/Em) using the Multimode Detector DTX 880 (Beckman Coulter, Brea, CA, USA). Images were captured with an inverted fluorescence microscope, and cell counts were analyzed with ImageJ 1.54g software.

### 2.7. Xenograft Studies

The animal studies were conducted in accordance with the principles of the Declaration of Helsinki. Ethical approval for this study was provided by the ethics committee of the Istituto Superiore di Sanità (Prot. D99977.35 Authorization 259-2017PR). Xenograft tumor growth was evaluated by measuring the shortest (d) and longest (D) diameters using a Vernier caliper. Tumor volume (TV) was calculated as TV (mm^3^) = d^2^ × D/2. Mice were sacrificed at the end of the study or when tumors reached 2000 mm^3^ or under conditions of ulceration, >20% body weight loss, or significant health deterioration, using cervical dislocation after isoflurane anesthesia.

For subcutaneous xenografts, 6 mice were injected with FGFR4_High_ and FGFR4_Low_ sorted CSCs, monitoring tumor growth for 8 weeks. For antibody-treated CSCs, mice received subcutaneous injections of a 5 × 10^6^ cells/mL suspension in Matrigel (BD Biosciences), with tumors treated twice weekly with ch3B6 antibody (25 mg/kg) or PBS (n = 9). Tumor volume and animal weight were measured twice weekly.

In the hepatic metastasis model, 2.5 × 10^5^ LUC-GFP-transduced CSC #85 cells were injected into the livers of 20 mice (lentiviral vector: pRRLsin-cPPT-hCMV-hPGK-GFP-Wpre encoding both GFP and luciferase, as previously described in [67]). Tumor growth was tracked by bioluminescence imaging. After 10 days, mice were assigned to two groups (ch3B6 and control) and treated IP with ch3B6 antibody (25 mg/kg) or PBS for 4 weeks. Tumor growth, body weight, and mouse health were monitored weekly. Mice were sacrificed after 2 additional weeks without treatment.

### 2.8. Generation of Rat Monoclonal Antibodies

Monoclonal antibodies (mAbs) against colorectal CSC membrane antigens were generated using genetic immunization of rats via the GENOVAC core technology platform. Target cDNAs were cloned into proprietary vectors: pB8-HA (for immunization, HA-tag) and pB1-myc (for detection, myc-tag), ensuring surface expression. For FGFR4 and IL17RB, RGMB full-length sequences were used, while for EDAR and OR51E1 only extracellular domains were cloned. BOSC23 cells (HEK293-derivatives) were transiently transfected with antigen vectors and reciprocal empty vectors as negative controls, verifying membrane localization by FACS with anti-tag antibodies and secondary goat anti-mouse IgG R-phycoerythrin (10 μg/mL). Additional controls included BOSC23 cells transfected with irrelevant cDNAs. For immunization, 8–12-week-old rats received 10 μg DNA/vector, gold-particle coated, delivered intradermally by gene gun, repeated weekly (4–6 doses depending on antigen). Serum was collected 24–60 days post immunization, diluted 1:1000, incubated with goat anti-rat IgG R-phycoerythrin-conjugated, and tested by FACS against BOSC23 cells transfected with the pB1-myc constructs to assess antibody titers and select responder animals. From immunoresponsive rats, splenic and lymph node B cells were fused with murine myeloma cells to generate hybridomas. Following clonal selection and expansion, hybridoma supernatants were screened by FACS on BOSC23 cells and subsequently on colorectal CSCs. The 4–8 best mAbs per antigen were prioritized and subcloned by limiting dilution. VH and VL regions of FGFR4-positive hybridoma subclones (1E7-C4, 3B6-E4, 5B5-G7, 5H9-D1, 6C9-C11, 8D4-E2, 8E3-E4, 10G11-F3) were PCR-amplified with a set of Aldevron’s proprietary primers and sequenced. Monoclonal antibodies were subsequently affinity-purified from serum-free hybridoma supernatants via HiTrap™Protein G-Sepharose chromatography (Cytiva, Malborough, MA, USA), on an ÄKTA system. IgGs were eluted under acidic conditions, neutralized, and quantified by BCA assay. IgG purity was confirmed by SDS-PAGE and Coomassie staining, with yields consistently exceeding 90%.

### 2.9. Flow Cytometry

Hybridoma supernatants positive against ectopically expressed antigens were analyzed by FACS (LSR II, Becton Dickinson, Franklin Lakes, NJ, USA) on five patient-derived colorectal CSC lines. In 96-well plates, 10^5^ cells/well were seeded and incubated with 200 µL of each supernatant. Negative controls included conditioned medium from murine myeloma cells, cells incubated with secondary antibody only, and 7-AAD (5 µg/mL) staining to monitor viability. After 1 h at 4 °C, cells were incubated with a PE-conjugated goat anti-rat IgG secondary antibody (Southern Biotech #3030-09, Birmingham, AL, USA). For purified antibodies, binding to FGFR4 was evaluated on FGFR4-positive colorectal CSC and hepatocarcinoma cell lines using dose–response assays (0.06, 0.12, 3, and 15 µg/mL). Experimental conditions and controls were identical to those used for supernatants. FACS data were analyzed with GraphPad Prism, and dissociation constants (K_D_) and maximum binding values (B_max_) were determined by fitting normalized fluorescence fold-change values (MFI of sample/MFI of negative control) to a one-site binding equation: Y = B_max_·X/(K_D_ + X). Assays with rat-human chimeric antibodies were conducted similarly, but detection used a PE-conjugated goat anti-human IgG secondary antibody (Southern Biotech, Birmingham, AL, USA). 

### 2.10. FGFR4-Dependent Solid-Phase ELISA

The binding apparent affinity and specificity of anti-FGFR4 monoclonal antibodies was evaluated by solid-phase ELISA using recombinant FGFR isoforms (FGFR4, FGFR1α/β IIIb–IIIc, FGFR2α/β IIIb–IIIc, FGFR3 IIIc; Biotechne/R&D Systems, Minneapolis, MN, USA). ELISA plates were coated with FGFR-Fc proteins (2 µg/mL) and blocked with PBS/3% BSA. Antibodies were added in serial dilutions and incubated in triplicates. Binding was detected with HRP-conjugated secondary antibodies (anti-rat IgG diluted 1:4000 for rat antibodies or anti-human Fab-specific diluted 1:2500 for chimeric rat-human antibodies) followed by TMB substrate development and termination with sulfuric acid. Absorbance was measured at 450 nm using a Multimode Detector DTX 880 (Beckman Coulter).

### 2.11. Solid-Phase FGF19 Competition ELISA

Anti-FGFR4 antibody’s ability to inhibit FGF19/FGFR4 binding was assessed using the previously described solid-phase assay with modifications. Antibodies or hybridoma supernatants were incubated 1 h in triplicate at 1:3 serial dilutions, followed by addition of equimolar amounts of FGF19 (Peprotech, Cranbury, NJ, USA) and low molecular weight heparin (23 nM, Sigma-Aldrich, St. Louis, MO, USA). After 2 h, plates were washed and incubated 1 h with a biotinylated anti-FGF19 antibody (1:1000). Following 5 washes, 50 μL of HRP-streptavidin (Thermo Fisher Scientific) were added for 30 min, washed, and TMB substrate incubated for 12 min. Reaction stopped with 100 μL 2 M H_2_SO_4_, and absorbance read at 450 nm as described.

### 2.12. RT-qPCR

Total RNA was extracted with the RNeasy Mini Kit (Qiagen, Hilden, Germany), quantified by A260 by an Eppendorf BioPhotometer (Eppendorf, Hamburg, Germany) and quality-checked via an Agilent 2100 bioanalyzer (Agilent Technologies). Human normal colon RNA (FirstChoice, Thermo Fisher Scientific) was used as control. Triplicate assays were performed in 96-well MicroAmp plates using TaqMan^®^ probes and primers (Applied Biosystems, Walthman, MA, USA). Reaction mix (50 µL) contained 2x Master Mix, 0.5 µL RT, 2.5 µL TaqMan Assay, H_2_O, and 10 µL RNA (5 ng/µL). RT-qPCR was run on a Perkin Elmer ABI 7900 HT (50 °C 30 min; 95 °C 10 min; 40 cycles 95 °C 15 s, 60 °C 1 min). Quantification used 2^−ΔΔCt^ method.

### 2.13. Immunoblotting

Cells were lysed in 1% SDS/PBS and protein concentration measured by BCA assay (Thermo Fisher Scientific). Equal protein amounts (15–35 µg) were separated by SDS-PAGE (NuPage 4–12% Bis-Tris, MES buffer) and transferred to nylon membranes. Membranes were blocked (5% milk in TBS-T) and incubated overnight at 4 °C with the following primary antibodies: anti-FGFR4 (rabbit anti-human, #2894 Cell Signaling, Danver, MA, USA), anti-IL17RB (rabbit anti-human, ab229320 Abcam, Cambridge, UK), anti-RGMB (rabbit anti-human, ab96727, Abcam, Cambridge, UK), anti-EDAR (rabbit anti-human, [EPR8020] ab137021 Abcam, Cambridge, UK), anti-OR51E1 (rabbit anti-human, ab97471 Abcam, Cambridge, UK), anti-phospho-FRS2 (rabbit anti-human, #3864; #3861, Cell Signaling, Danver, MA, USA), anti-Vimentin (rabbit IgG clone D21H3, #5741, Cell Signaling, Danver, MA, USA), anti-E-cadherin (rabbit mAb clone 24E10, # 3195, Cell Signaling, Danver, MA, USA), anti-beta Actin (mouse mAb #8226, Abcam, Cambridge, UK), and anti-GAPDH (mouse monoclonal G8795, Sigma-Aldrich, St. Louis, MO, USA). After washes, HRP-conjugated secondary antibodies were applied 1 h at RT, and bands were visualized by ECL using ChemiDoc MP (Bio-Rad, Hercules, CS, USA) or a Fusion FX-7 (Vilber, Paris, France). 

### 2.14. Production of Anti-FGFR4 Human-Rat Chimeric Antibodies

Human–rat chimeric antibodies (ch3B6, ch5B5, ch6C9) were generated by cloning rat VH and VL regions in frame with human IgG1 and kappa constant regions in modified gWIZ vectors (Genovac/Aldevron, Fargo, ND, USA). Inserts were sequence-verified. Heavy and light chain vectors were co-transfected into 293F cells in Expi293™ medium using ExpiFectamine™ 293 (Thermo Fisher Scientific). Antibodies were purified from ≥2 L supernatant via Protein-A Sepharose in PBS (pH 7.4), yielding 2–3 mg/mL. Purity was confirmed by SDS-PAGE and Coomassie staining; aggregates assessed by HPLC-SEC. Antibodies were sterile-filtered (0.22 µm), stored at −80 °C, and endotoxin levels were measured by LAL test (<0.1 EU/mL).

### 2.15. Identification of Anti-FGFR4 Antibody Binding Sites

FGFR4 extracellular binding sites were mapped using deletion mutants and peptide ELISA. Three deletion mutants (d1: aa 22–118; d2: aa 22–151; d3: aa 22–248) of human FGFR4 were cloned in pB1-myc vector and expressed in BOSC23 cells. Antibody binding was analyzed by FACS. For epitope mapping, overlapping 15-aa synthetic peptides covering Ig I (23 peptides, aa 22–118), acid box (26 peptides, aa 119–156), and Ig II (22 peptides, aa 157–241) domains were biotinylated and immobilized on streptavidin-coated 96-well plates. Wells were blocked (PBS/0.1% BSA/0.5% Tween-20), coated with peptides (50 µM, 2 h RT), and incubated with chimeric antibodies (150 µg/mL, 1.5 h RT), followed by HRP-conjugated goat anti-human IgG Fab (1:10,000, 1 h RT). After washes, chemiluminescent substrate was added and luminescence measured with a Multimode Detector DTX 880 (Beckman Coulter). Chimeric anti-myc antibody with its epitope peptide served as positive control.

### 2.16. Surface Plasmon Resonance (SPR) Analysis

Ch3B6 antibody was immobilized on a CM5 sensor chip of a Biacore T200 instrument (Cytiva) via standard EDC/NHS chemistry (10 µg/mL in 10 mM Na acetate, pH 4.5, 180 s). Fc1 served as reference (no antibody). Recombinant FGFR4 extracellular domains (dimeric FGFR4-Fc and monomeric FGFR4-His) were injected at 100 ng/mL–5 µg/mL (180 s, 60 µL/min) and dissociation monitored for 800 s. Regeneration was performed with 10 mM glycine, pH 2. Sensorgrams were corrected by subtracting Fc1 responses, and kinetic parameters (ka, kd) and affinity (K_D_) calculated using a 1:1 binding model (BIAevaluation 2.0.1). PBS formulation buffer was used as control.

### 2.17. Statistical Analysis

Mean and Standard Deviation (SD) were calculated using Microsoft Office Excel 2010 software (Microsoft Corporation, Redmond, WA, USA) or GraphPad Prism Software v10. To calculate apparent K_D_ values, data from ELISA assays were analyzed and fitted according to nonlinear regression, one site binding hyperbola curve, using GraphPad Prism software. Statistical significance was determined using the two-tailed Student’s *t* test or Anova test. A value of *p* ≤ 0.05 was considered statistically significant.

## 3. Results

### 3.1. Bioinformatic Identification of Membrane Protein Targets Enriched in Colorectal CSCs

To identify novel CSCs membrane antigens, a computational analysis was performed by mining proprietary CSC-specific Affymetrix microarray data and cross-querying available public expression databases.

This analysis took advantage of the availability of a genome-wide gene expression data bank (Affymetrix microarray, Human Gene 1.0 ST platform), including data obtained from eight colorectal CSC lines (#1.1, #1.2, #18, #85, #CRO1, #CC1, #CC2, #CC5) isolated from primary tumor samples and metastases that were propagated as spheroids in defined serum-free media complemented with EGF and bFGF (sCSC), or differentiated in vitro in the presence of 10% serum (dCSC) [17]. This dataset contains 28,231 distinct probe sets/transcripts for 26,917 different human genes.

**Figure 1:**
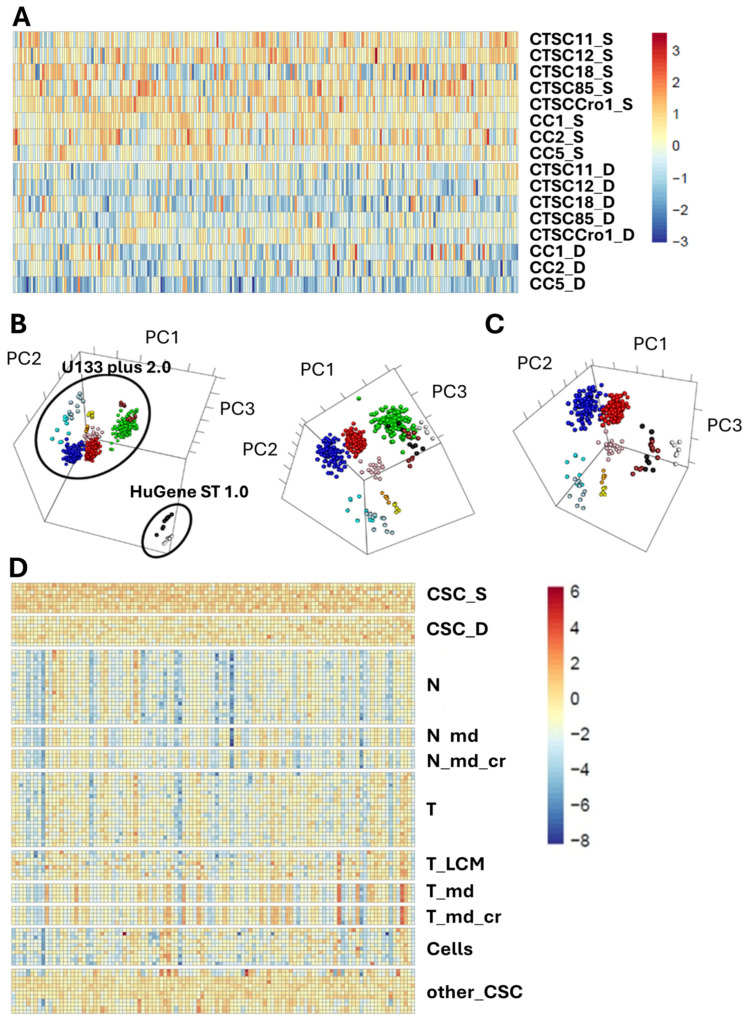
Bioinformatic identification of CSC-enriched gene targets. (**A**) Heatmap of 250 candidate genes differentially expressed between undifferentiated (CSC_S, top) and differentiated (CSC_D, bottom) colorectal CSCs. Values are shown as Z-scores relative to the median expression across both groups. (**B**) PCA of normal and tumor colon datasets before (left) and after (right) ComBat batch correction. CSCs from the current study (HuGene St 1.0, black), published CSCs (U133 plus 2.0, brown), and human stem cells (HuGene ST 1.0, white) are shown. Other samples include macrodissected normal (blue) and tumor (red) tissues, microdissected normal (light blue), microdissected normal crypt (turquoise), laser-capture microdissected tumor (pink), microdissected tumor (orange), microdissected tumor crypt (yellow) samples and colon cancer cell lines (green). (**C**) PCA after ComBat correction, excluding cell lines for clarity. (**D**) Heatmap showing expression of 103 shortlisted genes across representative samples, including normal colon (N), colon tumors (T), laser-microdissected tumors (T_LCM), and colon cancer cell lines.

To define a list of potential target genes, the following overall workflow was adopted. Using the colorectal CSC dataset, a first list of genes (1325) differentially up-regulated in the undifferentiated colorectal CSCs with respect to their in vitro differentiated counterparts was generated including only genes with a statistically significant differential expression (pfp-value ≤ 0.1). The target gene list was then reduced to 250 entries annotated as genes encoding potential trans-membrane proteins or GPI-anchored proteins, according to two different sources (https://www.uniprot.org/ and [63]). Within this gene list it was evident that the up-regulation levels in the undifferentiated cell samples compared with their differentiated counterparts were generally low and often less than 2-fold (Figure 1A).

Therefore, an additional prioritization strategy, taking advantage of the vast number of public gene expression data, was implemented with the main focus on genes up-regulated in the undifferentiated colorectal CSC samples compared to normal colon tissue. In addition, colon tumor tissue samples and commercial tumor cell line samples were included as further selection criteria towards CSC specificity.

To this aim, public gene expression data repositories (NCBI GEO and EBI ArrayExpress) were searched for datasets containing human colon tissue samples (normal or tumor) or data for commercially available colon cancer cell lines (Figure S1). No sub-selection was performed with respect to the exact anatomic localization of the tumor samples (where available) used to generate accessible data. For the tumor category, only carcinoma samples were accepted excluding all identifiable adenoma samples or other clearly distinct samples, e.g., samples from inflammatory bowel disease patients.

Overall, the global dataset comprised a total of 200 normal samples, 20 of which were obtained by LCM, 1100 tumor samples (87 of which obtained by LCM) from 16 different datasets, and 145 colon tumor cell line samples (74 unique cell lines) from 8 different datasets. In addition, 6 datasets containing human normal and tumor stem cell samples were included. Only two datasets have the same microarray architecture (HuGene 1.0 ST) as the reference colorectal CSC dataset, while all other datasets were based on the Affymetrix U133 plus 2.0 platform.

To correct technical variability between platforms, we applied the ComBat algorithm [65]. Principal component analysis confirmed improved clustering by biological category rather than platform post-correction (Figure S2A). After balancing normal and tumor groups with equal number of samples, i.e., excluding data farthest from the clusters’ centers (Figure S2B,C), these batch-corrected sub-datasets (Table S1, “ComBat” sheet) were combined with our proprietary colorectal CSC dataset. The inclusion of an additional colon CSC dataset on cells closely related to ours, which used the Affymetrix U133 plus 2.0 platform (NCBI GEO GSE33112) [68], enabled further alignment between the two Affymetrix platforms through ComBat. Furthermore, an additional human stem cell dataset (NCBI GEO GSE21244) [69] generated with the same “HuGene 1.0 ST” platform as our colon CSC data was also included as reference. As illustrated in Figure 1B,C, the dataset initially exhibited a clustering pronouncedly based on microarray platform rather than biological origin. Following ComBat correction, this platform-specific bias was markedly reduced, resulting in the colorectal CSC samples aligning more closely with the corresponding colon CSC published dataset, while preserving the relative distribution of normal, tumor, and cell line samples.

Following normalization, average fold changes (FC) between the undifferentiated CSC samples and the other subsets (macro-dissected colon normal and tumor datasets, micro-dissected colon normal and tumor datasets, and colon tumor cell line dataset) were calculated using the RankProd algorithm [61]. Genes with a pfp-value > 0.1 were excluded, and 250 genes were retained (Table S1, “250 selected genes” sheet). Then, a selection of those consistently upregulated in CSCs relative to both normal and tumor samples narrowed this number to 103 genes (Table S1, “103 selected genes” sheet). Their gene expression pattern across the whole dataset are shown in Figure 1D. From the resulting list of 103 genes, those with predicted plasma membrane localization were further selected, narrowing down the list to 56 candidates based on (1) expression fold-change (≥2) compared to colon tumor tissues and cell lines; (2) low expression in vital normal tissues; (3) limited shedding variants; and (4) existing knowledge on their function and interaction networks (Table S1, “56 CSC targets Score” sheet). Each of the 56 candidates was rated as positive (YES), intermediate (MAYBE), or negative (NO), based on available information. The 20 genes identified with “NO” were excluded from the study. Among the remaining 36 candidates (18 identified with “YES” and 18 with “MAYBE”) (Table S1, “36 CSC targets Score” sheet), 11 were expressed in one or more vital organs but showed an acceptable profile in all other respects, making them potential targets for the generation of monoclonal antibodies for diagnostic purposes or for monitoring CSCs during therapy (B = “Biomarker”). In addition, 8 genes were included which, although showing significantly higher expression levels in colorectal CSC lines than in normal colon tissues, did not present sufficient specificity with respect to tumor tissues and cell lines. The bioinformatic analysis of these 36 genes is reported in Table S1, “36 genes relative expression” sheet. Further screening based on immunogenicity, ectopic expression, and similarity to human paralogs led to a final list of 21 candidate genes, summarized in Table S1, “Final 21 targets” sheet.

### 3.2. Experimental Validation of Selected Targets Specificity in CRC CSCs

**Figure 2:**
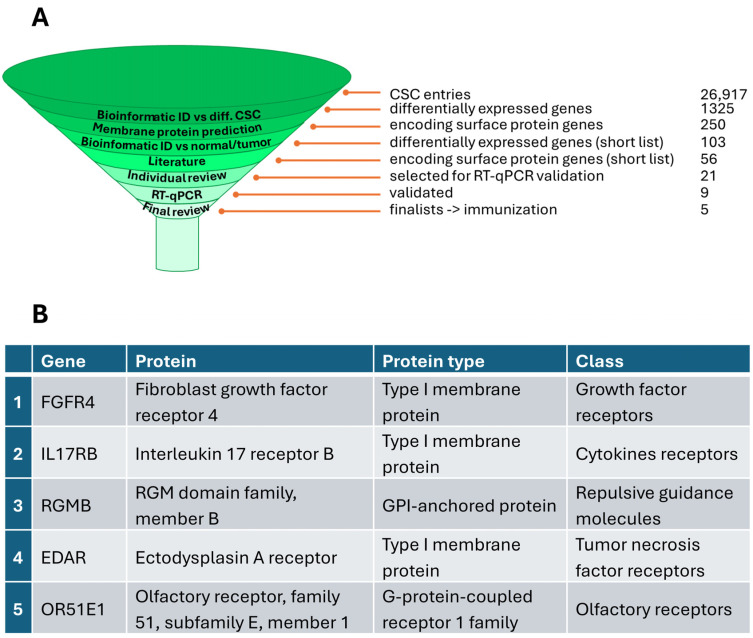
Prioritization of colorectal CSC membrane targets. (**A**) Gene selection funnel combining transcriptomic filtering, membrane localization criteria, and tissue specificity. (**B**) List of five selected candidate genes with their protein names, classes, and categories.

The differential expression of the top 21 candidate genes was validated via RT-qPCR using total RNA preparations isolated from the eight colorectal CSC lines and eleven commercial colon cancer cell lines listed in Table S2, as well as RNA from normal colon tissue (indicated as “n. RNA”). First, each gene expression was normalized to housekeeping genes expression (GAPDH or HNRNPK) whose expression was found relatively stable in Affymetrix data across cell lines. For each gene, normalized mRNA levels were measured in undifferentiated colorectal CSCs, in commercial tumor cell lines, and in normal colon tissue. The RT-qPCR values obtained for each gene in the cell lines and in normal tissue were then expressed as a percentage relative to their expression in undifferentiated CSCs. In this way, a gene specifically expressed in CSCs would appear with very low relative expression in differentiated tumor cells or normal colon, highlighting its stem-specific pattern. Table S3 summarizes these data, categorizing each gene based on how many CSC lines showed at least a 3-fold higher expression as compared to normal colon in at least four cancer cell lines. Based on these results, seven genes met the specificity criteria in at least three independent CSC lines and were therefore considered colorectal CSC-specific, experimentally “acceptable” (in orange in Table S3). Two additional genes, KIAA1524 and LRP8, while less CSC-specific, were notably overexpressed in tumor lines and were therefore also retained for further analysis (light orange in Table S3). Lastly, the top nine RT-qPCR validated genes were narrowed down to five, based on their predicted immunogenicity. The funnel diagram in Figure 2A summarizes the whole selection process, and Figure 2B lists the final five candidates and their main features.

### 3.3. Prioritization of FGFR4 as a Therapeutic Target

**Figure 3:**
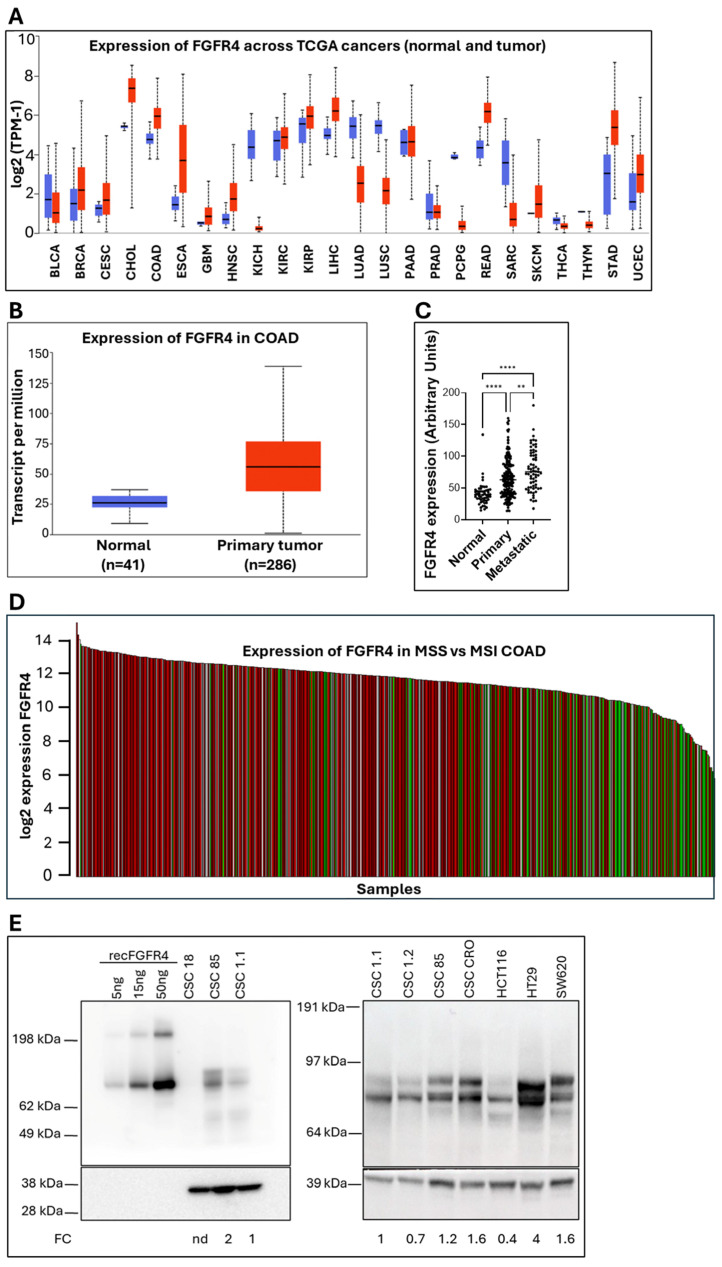
FGFR4 expression across tumor datasets. (**A**) FGFR4 expression in TCGA pan-cancer transcriptomes, with highest levels in cholangiocarcinoma (CHOL), colon adenocarcinoma (COAD), rectum adenocarcinoma (READ), and liver HCC (LIHC). Blue: normal samples; red: tumor samples. (**B**) FGFR4 is significantly upregulated in COAD tumors versus normal colon (*p* < 1 × 10^12^). Blue: normal samples; red: tumor samples. (**C**) Analysis of differences in FGFR expression among normal colonic mucosa, primary colorectal tumors, and metastatic lesions from GEO dataset GSE41258. Statistical significance was determined using one-way ANOVA followed by Tukey’s HSD post hoc test. ** *p* < 0.01; **** *p* < 0.0001. (**D**) FGFR4 expression stratified by MSI status in COAD: high in MSS (red), low in MSI-H (green). In white MSI-L samples are also shown. (**E**) Western blot analysis of FGFR4 expression in colon CSCs compared to commercial colon cell lines; 5–15–50 ng of the human recombinant FGFR4-Fc fusion protein was loaded as control. Bands quantification was normalized to GAPDH and expressed as FC, where all samples are compared to CSC #1.1, which therefore is represented as 1. The uncropped blots are shown in File S1.

Among the five top-ranked candidates, FGFR4 was selected as the initial target for further investigation, based on integrative internal analyses, including both bioinformatic and experimental data, and as it is a member of a well-characterized family of oncogenic receptors. Analysis of The Cancer Genome Atlas (TCGA) pan-cancer dataset revealed elevated FGFR4 mRNA levels in CRC (COAD), with significantly higher expression in tumor tissues compared to normal colon (Figure 3A,B). When compared to the other four shortlisted targets, IL17RB, RGMB, EDAR, and OR51E1, FGFR4 consistently showed the greatest tumor-to-normal expression difference and the highest absolute expression levels in both the pan-cancer and colon-specific datasets (Figure S3A,B).

Moreover, we evaluated FGFR4 gene expression using data from an independent GEO dataset (GSE41258), which comprises approximately 390 samples profiled on the Affymetrix Human Genome U133A platform. This dataset was selected because it includes tissue specimens representing distinct stages of CRC progression analyzed under uniform experimental conditions, allowing for direct comparisons. Specifically, the cohort consists of 54 samples of normal colonic mucosa, 186 primary colon adenocarcinoma samples, and 67 metastatic samples, including 47 liver metastases and 20 lung metastases. Our analysis demonstrated a stepwise increase in FGFR4 expression from normal tissue to primary tumors, with the highest expression observed in metastatic lesions (Figure 3C). These differences were statistically significant, as assessed using one-way analysis of variance (ANOVA *p* < 0.001). Post hoc analysis using Tukey’s HSD test showed significantly higher FGFR4 expression in primary tumors compared with normal tissue (*p* < 0.0001), with a further significant increase in metastatic lesions compared to primary tumors (*p* = 0.0068). All these results indicate that an increase in FGFR4 expression is associated with CRC progression and further support its potential as a therapeutic target.

Further stratification by microsatellite instability (MSI) status showed FGFR4 expression to be lowest in MSI-High (MSI-H) tumors and highest in microsatellite stable (MSS) tumors (Figure 3D). This finding is particularly relevant as MSS status characterizes 80–85% of CRC cases, which are generally less responsive to immune checkpoint inhibitors [70] and more prone to recurrence [71], suggesting potential therapeutic benefits of FGFR4 targeting in a wide pool of CRC patients.

The expression of FGFR4 in colon CSC lines was analyzed by Western blot in comparison to tumor cell lines, namely HCT116, HT29, and SW480. In parallel, 5, 15, and 50 ng of a recombinant FGFR4-Fc fusion protein was loaded as quantitative control (Figure 3E). Among the five colon CSC lines, 85 and CRO showed the highest expression level, while #18 showed almost undetectable levels (Figure 3E).

Finally, the analysis of published clinical studies demonstrated that high FGFR4 expression in colorectal carcinoma is significantly correlated with a poorer prognosis, acting as an independent predictive factor for reduced overall survival (OS) and recurrence-free survival (RFS). For example, Li et al. [28] showed that a Kaplan–Meier analysis of 316 CRC patients in which FGFR4 positivity was significantly correlated with shorter disease-free survival (DFS). Also, Ye et al. in 2020 [52] showed that the overall survival time of patients with FGFR4 high expression at tumor stages T2 and T3 was significantly poorer compared with those with FGFR4 low expression, with patients with FGFR4 high expression having a lower 5-year survival time compared with patients with FGFR4 low expression (64 vs. 74%).

Based on the totality of available data, FGFR4 was selected as a high-priority target for therapeutic intervention in CRC.

### 3.4. Functional Assessment of FGFR4 Role in Colorectal CSCs Enriched for and Depleted of FGFR4

Since the culture medium optimized for colorectal CSCs is complemented with “basic fibroblast growth factor” (bFGF/FGF2) [17], a potential ligand of FGFR4, starvation experiments were conducted to rule out the possibility that FGFR4 expression was induced by bFGF in the culture medium. Four colorectal CSC lines were grown in standard spheroid-forming medium with or without bFGF for at least one duplication time (3–7 days depending on the cell line). Total RNA was isolated, quantified, and quality-checked, and FGFR4 expression was measured by RT-qPCR (Figure S4). No significant differences in FGFR4 expression were found between the conditions, particularly in the #85 CSC line, validating the use of the stem cell medium in the evaluation of FGFR4 in CRC CSCs.

**Figure 4:**
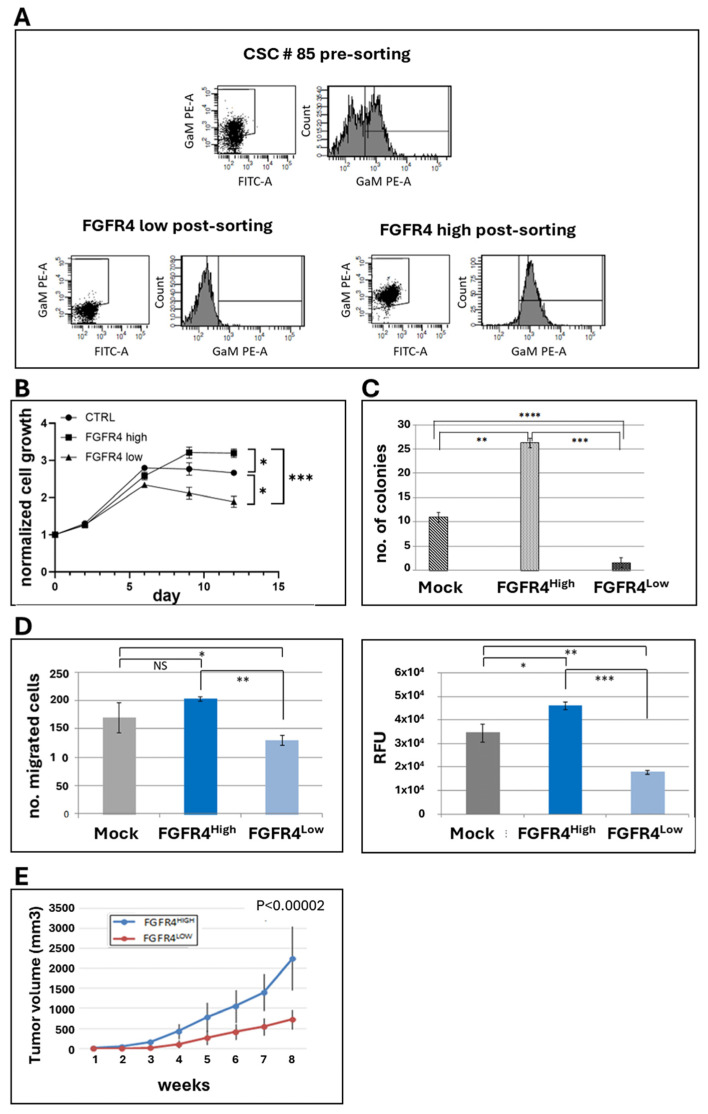
Functional role of FGFR4 in colorectal CSCs. (**A**) FACS-based sorting of CTSC 85 cells into FGFR4_High_ and FGFR4_Low_ populations. (**B**) Growth curves comparing mock-sorted, FGFR4_High_, and FGFR4_Low_ cells (n = 3; CellTiter-Blue assay). (**C**) Colony formation in soft agar; FGFR4_High_ cells show significantly higher clonogenicity (*p*-values as indicated). (**D**) Transwell migration assay with Calcein AM staining; FGFR4_High_ cells display increased migration (*p* < 0.04 to *p* < 0.001). (**E**) Tumor growth curves of FGFR4_High_ vs. FGFR4_Low_ cells in subcutaneous xenografts. Data shown as mean ± SD. NS: not significant; * *p* < 0.05; ** *p* < 0.01; *** *p* < 0.001; **** *p* < 0.0001.

To investigate the role of this receptor in colorectal CSC biology, we isolated FGFR4_High_ and FGFR4_Low_ subpopulations from the #85 CRC CSC line using flow cytometry (Figure 4A). These subpopulations were subjected to several functional assays alongside the unsorted parental population as a reference. In proliferation assays, FGFR4_High_ cells demonstrated an extended exponential growth phase and reached significantly higher cell densities compared to FGFR4_Low_ and parental cells over a 14-day culture period (Figure 4B). In addition, FGFR4_High_ cells showed higher clonogenic efficiency (Figure 4C) and migratory capacity (Figure 4D) when compared to FGFR4_Low_ cells. In fact, the knockdown of the receptor in CRC cells resulted in a significant reduction in clonogenic ability and impaired migration in Transwell assays, indicating that the receptor is relevant for tumorigenicity and metastatic potential. To validate these observations in vivo, equal numbers of FGFR4_High_ and FGFR4_Low_ CSC 85 cells were injected subcutaneously into opposite flanks of immunodeficient NOD/SCID mice. After eight weeks, tumors derived from FGFR4_High_ cells were significantly larger, confirming a higher tumorigenic capacity compared to FGFR4_Low_ cells (Figure 4E).

Together, these results show that FGFR4 expression characterizes a tumor cell population with enhanced tumorigenic potential, thereby reinforcing its prioritization as a therapeutic target and prompting us to the characterization of the monoclonal antibodies generated against this protein among all those raised against the five top targets.

### 3.5. Generation of Monoclonal Antibodies Against the Selected CSC Antigens and Screening of FGFR4 Binders

To evaluate the therapeutic potential of the five top candidates, monoclonal antibodies were generated using rat genetic immunization via the GENOVAC platform (https://genovac.com/solutions/immunization-strategies/). This method allows antibody development against native, correctly folded, and glycosylated proteins directly from their coding DNA sequences. Full-length cDNAs for FGFR4, IL17RB, and RGMB, as well as extracellular domains of EDAR and OR51E1, were cloned into proprietary expression vectors (pB8-HA for immunization, pB1-myc for detection). Transient transfection of BOSC23 cells confirmed correct plasma membrane expression of all five constructs via FACS analysis (Figure S5A). Immunized rat sera showed strong antigen-specific reactivity, confirming successful immunogenicity across all targets (Figure S5B). Hybridomas were then generated from antigen-reactive B cells by fusion with myeloma cells. Between 14 and 70 antibody-containing supernatants were produced per target and tested via FACS for specific binding to the BOSC23 transfected cells, yielding multiple positive hits for each antigen.

For FGFR4 a total of 70 hybridoma supernatants were screened across four FGFR4-positive colorectal CSC lines (#1.1, #1.2, #85, #CRO1) and one FGFR4_Low_ cell line (#18) using flow cytometry (Figure S6A). To aid preclinical translation, cross-reactivity to murine FGFR4 was evaluated early in the screening process. Of the 70 human-reactive antibodies, 50 also recognized the murine receptor—an important feature to assess the therapeutic index of an antibody in in vivo efficacy studies. Eighteen top-ranking supernatants were selected for further characterization, 14 of which demonstrated cross-species reactivity. These candidates were then tested in a solid-phase ELISA for their ability to inhibit FGF19 binding to immobilized FGFR4. Eight antibodies showed clear dose-dependent inhibition, including six cross-reactive (1E7, 3B6, 5B5, 5H9, 6C9, 10G11) and two human-specific (8D4, 8E3) clones (Figure S6B). Notably, these same antibodies also ranked among the highest for cell surface binding in the initial screen. From the top-performing hybridomas, eight stable clones were established: 1E7-C4, 3B6-E4, 5B5-G7, 5H9-D1, 6C9-C11, 8D4-E2, 8E3-E4, and 10G11-F3. Variable region sequences (VH and VL) were amplified and analyzed, revealing unique complementarity-determining regions (CDRs) for each antibody (Figure S7). Interestingly, 1E7-C4 and 3B6-E4 shared identical VH CDR1 and CDR2 sequences, differing in CDR3. Their light chains also displayed high similarity, suggesting a common progenitor. Similarly, 5B5-G7 and 6C9-C11 differed by a single amino acid in VL CDR1, indicating close clonal relatedness. The remaining clones presented more distinct CDR profiles.

### 3.6. Functional Characterization of Purified Rat Anti-FGFR4 Monoclonal Antibodies

The corresponding monoclonal antibodies were purified by protein G-Sepharose affinity chromatography and evaluated in several in vitro assays to select the more efficient ones for further characterization. First, they were evaluated for binding to FGFR4-expressing colorectal CSC lines (#1.2, #85, #CRO1) and the FGFR4-overexpressing Huh7 HCC line. Flow cytometry confirmed strong, dose-dependent binding for 3B6-E4, 5B5-G7, and 6C9-C11, with EC_50_ in the low nanomolar range (Table 1). Other clones, including 8E3-E4, showed weaker or less specific binding.

In parallel, the antibodies were tested in a solid-phase FGF19-FGFR4 binding inhibition assay. All clones demonstrated low nanomolar IC_50_ values, indicating efficient blockade of ligand–receptor interaction (Table 2).

**Figure 5:**
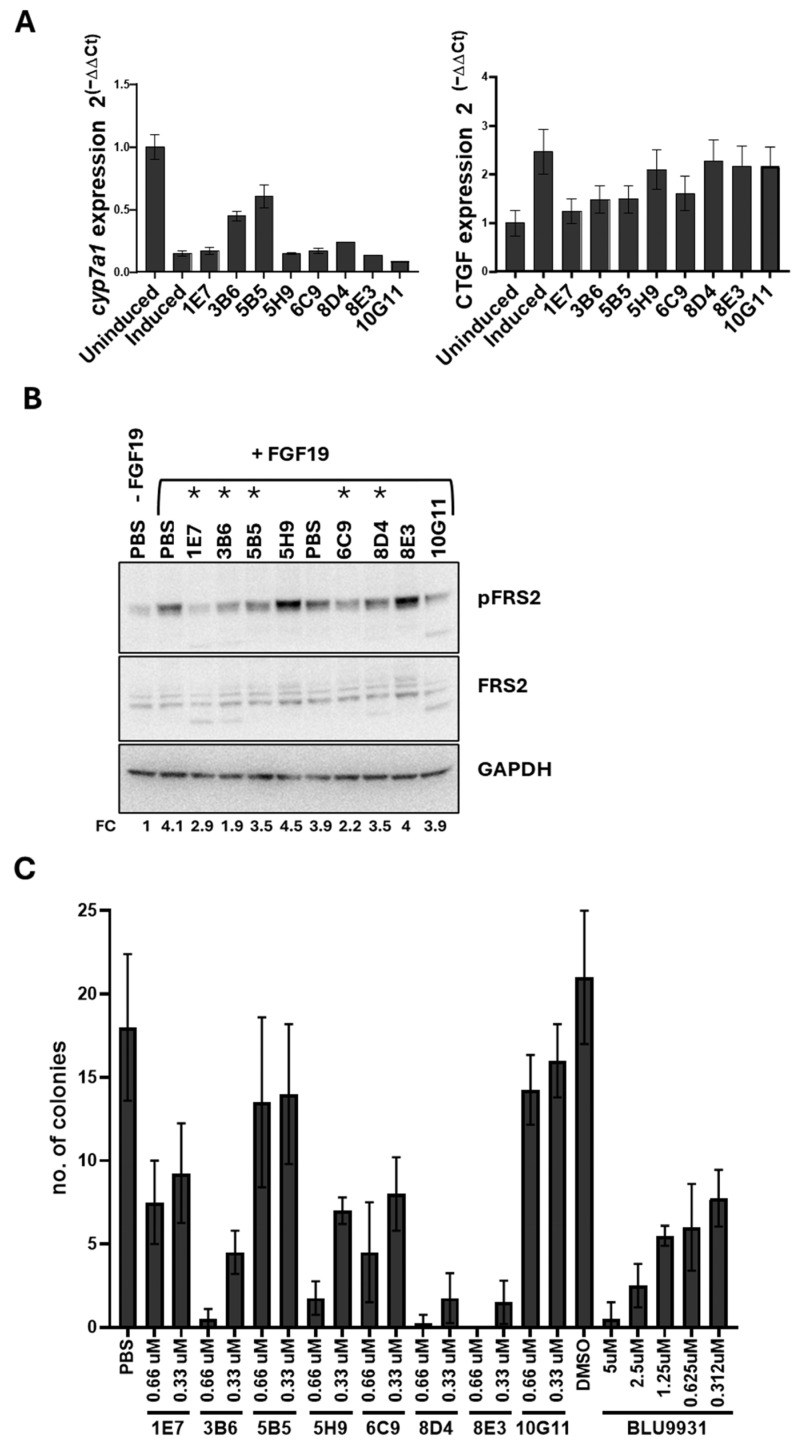
Functional characterization of anti-FGFR4 monoclonal antibodies. (**A**) RT-qPCR in HepG2 cells pretreated with antibodies, followed by FGF19 stimulation. CYP7A1 and CTGF transcript levels shown relative to controls. (**B**) Western blot of FRS2 phosphorylation in Hep3B cells, assessing FGFR4 pathway inhibition by each antibody. Bands were quantified and normalized as ratio (FC) between pFRS2 and total FRS2; (*) active antibodies. (**C**) Colony formation assay in Huh7 cells treated with antibodies or FGFR4 inhibitor BLU9931. Data normalized to vehicle controls. The uncropped blots are shown in File S1.

To assess the antibodies’ ability to inhibit the FGF19/FGFR4 pathway, their effects were tested on human HCC cells, which rely on this signaling for proliferation and survival. The mRNA expression of two downstream genes, i.e., the cholesterol 7a-hydroxylase (CYP7A1) and the connective tissue growth factor (CTGF), regulated by this pathway both in normal and tumor liver cells [72,73], was measured via RT-qPCR upon treatment with FGFR4-specific mAbs (Figure 5A).

FGF19 suppressed CYP7A1 and induced CTGF as expected; treatment with 1E7, 3B6, 5B5, 6C9, or 8D4 antibodies reversed these effects, restoring CYP7A1 and reducing CTGF levels. Western blot analysis of FRS2 phosphorylation further confirmed pathway inhibition by 1E7, 3B6, and 6C9—particularly 3B6—and to a lesser extent by 5B5 and 8D4 (Figure 5B). Lastly, the antiproliferative activity of the antibodies was assessed in a clonogenic assay using Huh7 cells. Representative results are shown in Figure 5C. Continuous treatment with 3B6, 5B5, 5H9, 6C9, 8D4, and 8E3 reduced colony formation to levels comparable to or better than the reference FGFR4 inhibitor BLU9931. These results highlighted 3B6, 5B5, and 6C9 as lead candidates based on binding affinity to colorectal CSCs in the low nanomolar range, pathway inhibition, and antiproliferative efficacy at concentrations compatible with standard therapeutic doses of antibody drugs.

### 3.7. Generation of Anti-FGFR4 Human-Rat Chimeric Antibodies

To advance the lead antibodies toward therapeutic development, the variable regions (VH and VL) of 3B6, 5B5, and 6C9 were cloned into expression vectors containing human IgG1 heavy and light kappa constant regions, respectively, generating chimeric antibodies ch3B6, ch5B5, and ch6C9. The constructs of each heavy and light chain couple were co-transfected into 293-F cells, and the resulting antibodies were purified via Protein A affinity chromatography (Figure S8).

**Figure 6:**
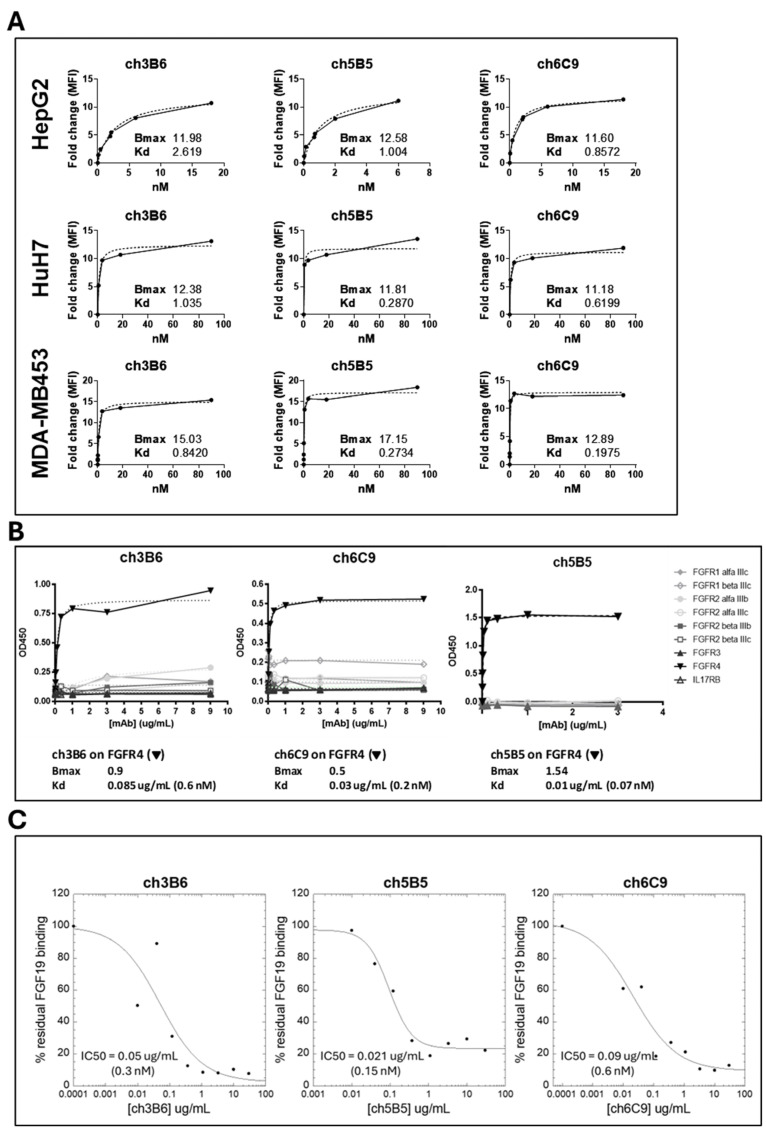
Binding specificity and apparent affinity of chimeric anti-FGFR4 antibodies. (**A**) FACS-based dose–response binding curves for ch3B6, ch6C9, and ch5B5 on HepG2, Huh7, and MDA-MB453 cells. (**B**) ELISA-based specificity assay against FGFR1-4 and control receptor. Binding curves fitted to a one-site model. For both (**A**) and (**B**) panels, dots represent experimental data, solid lines connect data points for visual guidance only, whereas dotted lines represent the nonlinear regression fit used to determine apparent affinity values. (**C**) Inhibition of FGF19-FGFR4 binding by chimeric antibodies measured in solid-phase competition assay.

Flow cytometry confirmed that the chimeric antibodies retained strong binding to FGFR4 on Huh7 and HepG2 HCC cells, with similar apparent dissociation constants in the low nanomolar range as the parental rat antibodies (Figure 6A).

Chimeric antibodies were also tested on the MDA-MB453 breast cancer line, which expresses the ligand-independent FGFR4 Y367C mutant [74]. Notably, they effectively bound this dominant, ligand-independent, constitutively activated variant that is known to be insensitive to the inhibition by a known antagonistic antibody, supporting its potential utility against tumors with constitutively active FGFR4, beyond CRC.

Solid-phase binding assays against FGFRs receptors other than FGFR4 revealed high specificity: all three chimeric antibodies exhibited sub-nanomolar affinity for FGFR4, with negligible binding to the other FGFR isoforms. Representative dose–response binding curves are shown in Figure 6B. In parallel, ch3B6 and its counterparts retained the ability to block in vitro FGF19 binding to FGFR4-Fc (Figure 6C), consistent with the parental antibody data.

### 3.8. Epitope Mapping of Chimeric Antibody Binding Sites and Binding Kinetics of the ch3B6 Lead Antibody by Surface Plasmon Resonance

**Figure 7:**
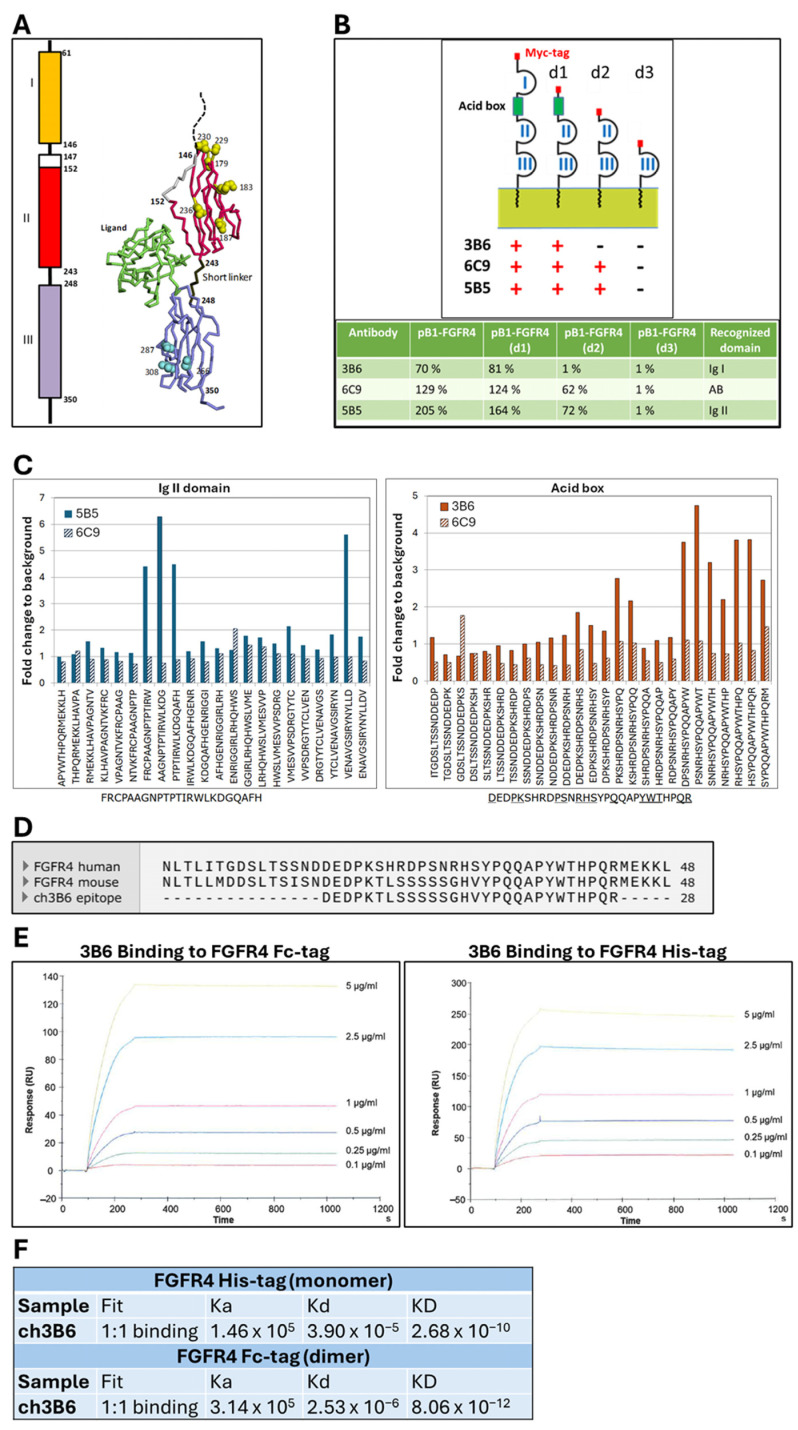
Epitope mapping of chimeric anti-FGFR4 antibodies and ch3B6 kinetics in SPR. (**A**) Schematic representation of the human FGFR4 extracellular region with Ig domains I, II, and III highlighted in orange, red and violet, respectively. The FGFR4 ligand (green) complex was obtained by superimposing a model of FGFR4 domains II (red) and III (violet) onto the corresponding domains from FGFR1 (PDB entry 1EVT). Amino acids that differ in human and mouse FGFR4 are highlighted in yellow and blue, respectively, in Ig II and III. (**B**) Binding of chimeric antibodies to full-length and deletion mutants (d1–d3) of FGFR4 in BOSC23 cells. (**C**) ELISA-based epitope mapping on overlapping peptide arrays. Binding sequences identified for ch3B6 and ch5B5. (**D**) Sequence alignment of the human and mouse FGFR4 acid box region showing mapped binding epitope. (**E**) Kinetic binding constants and affinity of the ch3B6 antibody by Surface Plasmon Resonance: sensorgrams representing the dose–response binding of FGFR4-Fc (left) and FGFR4-His (right) to ch3B6. (**F**) Kinetic association (ka) and dissociation (kd) rates and affinity constants (K_D_) of FGFR4-Fc and FGFR4-His were calculated applying a 1:1 binding as fitting model using the Bia T200 evaluation software 2.0.1.

A prototypical FGFR extracellular region contains three Ig-like domains (Ig I–III) joined by flexible linkers, with the Ig I–II linker hosting the glutamate/aspartate/serine-rich ‘acid box’ (AB) (Figure 7A).

To identify the binding epitopes, three deletion mutants of the FGFR4 extracellular region were generated: d1 (Ig I domain removed), d2 (Ig I + AB/linker removed), and d3 (Ig I, AB/linker, and Ig II domains removed) (Figure 7B). They were expressed in BOSC23 cells, and the binding of the chimeric antibodies to each deletion domain was assessed by FACS. All three chimeric antibodies bound full-length FGFR4 and the d1 mutant. However, ch3B6 failed to bind d2, indicating a binding site within the acid box region between Ig I and Ig II (Figure 7B). The other two antibodies retained reactivity towards the d2 mutant but resulted negative on d3, thus showing a binding specificity for the Ig II domain. This domain specificity was further confirmed using arrays of overlapping synthetic peptides spanning the Ig I, or AB and Ig II domain, in ELISA assays. As expected from the FGFR4 deletion analysis, none of the chimeric antibodies showed a specific reactivity on peptides corresponding to the Ig I domain, while the antibodies ch6C9 and ch5B5 resulted negative also on peptides corresponding to the AB/linker (Figure 7C). ch6C9 showed a low reactivity, distributed in multiple peaks on peptides corresponding to the Ig II domain, suggesting that it recognizes a discontinuous/conformational epitope in this region. On the contrary, ch5B5 showed a high and specific reactivity on three overlapping peptides within domain Ig II, thus defining a linear epitope of 23 amino acids (Figure 7C). Interestingly, different from the other antibodies, ch3B6 showed a clear positivity on specific peptides within the AB/linker, thus confirming the FGFR4 deletion data. Although in this case the reactivity was distributed within a discrete number of peaks, it was, however, possible to define for this antibody a discontinuous binding site within the AB/linker comprising amino acids between 127 and 154 (Figure 7C). The alignment of the human FGFR4 protein sequence with the mouse orthologous sequence, depicted in Figure 7D, shows that the key residues contributing to the binding within the experimentally identified ch3B6 epitope are conserved in the mouse sequence, thus supporting the observed cross-reactivity of the antibody for the murine receptor. The acid box has been implicated in auto-inhibition of FGFR signaling by mimicking heparan sulfate interaction sites, suggesting that ch3B6 may function by stabilizing FGFR4 in an inactive conformation or by inhibiting ligand binding through steric hindrance.

To quantitatively assess its binding affinity, the ch3B6 antibody was immobilized via amine coupling on a CM5 sensor chip for surface plasmon resonance (SPR) analysis. Two recombinant forms of the FGFR4 extracellular domain—FGFR4-Fc (dimeric) and FGFR4-His (monomeric)—were injected at increasing concentrations (100 ng/mL to 5 µg/mL). Association and dissociation were monitored over 180 and 800 s, respectively, and a 1:1 binding model was applied to calculate kinetic constants (Figure 7E). ch3B6 showed sub-nanomolar affinity for both FGFR4 variants, primarily due to a very slow dissociation rate (Figure 7F). As expected, binding to the monomeric FGFR4-His was slightly weaker compared to the dimeric FGFR4-Fc, which exhibited an avidity-enhanced signal. The extremely low dissociation rate observed for the FGFR4-Fc interaction exceeded instrument detection limits, preventing accurate kinetic modeling for this configuration. Nonetheless, the data confirmed ch3B6 as a high-affinity binder, capable of engaging both monomeric and multimeric FGFR4 in a concentration-dependent manner.

### 3.9. Inhibition of Colorectal CSCs Proliferation by ch3B6 Antibody

**Figure 8:**
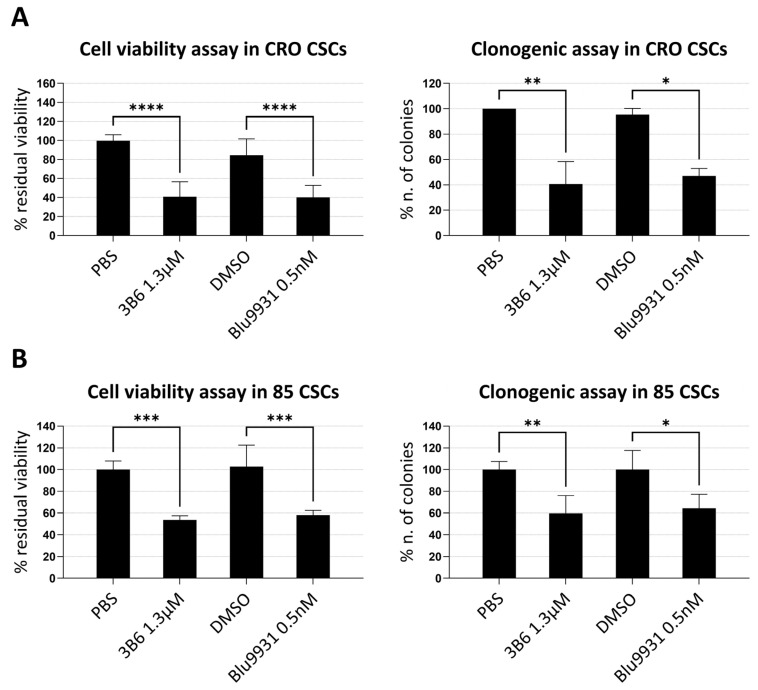
Inhibitory effects of ch3B6 antibody on colon CSC proliferation. (**A**) Inhibition of cell viability of colon #85 (left panel) and #CRO1 (right panel) CSCs upon treatment with ch3B6 or BLU9931. (**B**) Inhibition of colony forming efficiency of colon #85 (left panel) and #CRO1 (right panel) CSCs upon treatment with ch3B6 or BLU9931. * *p* < 0.05; ** *p* < 0.01; *** *p* < 0.001; **** *p* < 0.0001.

The ability of ch3B6 to inhibit FGFR4-dependent colorectal CSC growth was evaluated using cell viability and colony formation assays in two representative CSC lines, #CRO1 and #85 (Figure 8).

Cells were cultured for 8 days in the presence or absence of ch3B6 antibody. The selective FGFR4 tyrosine kinase inhibitor BLU9931 was included as a reference. Following treatment, overall viability was measured using the CellTiter-Glo™ luminometric assay, while colony formation was quantified by crystal violet staining and counting. As shown in Figure 8, ch3B6 significantly reduced both cell viability (Figure 8A) and clonogenic potential (Figure 8B) both in #CRO1 cells (right panels) and CTSC 85 (left panels) cells, even if to a lesser extent in this latter cell line. The degree of inhibition observed was comparable to that achieved with BLU9931 and in all cases statistically significant. These findings further support the specificity and functional relevance of ch3B6 as a targeted therapeutic candidate for FGFR4-positive colorectal CSCs.

### 3.10. Inhibition of FGFR4-Dependent FRS2 Phosphorylation in Colorectal CSCs Treated with ch3B6 Antibody

**Figure 9:**
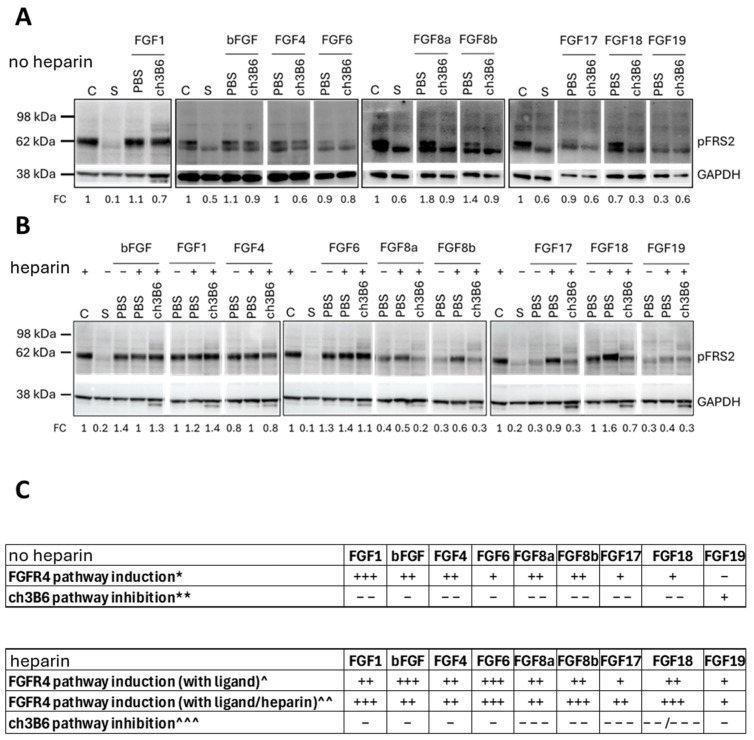
Inhibition of FGFR4 downstream signaling in CSCs by ch3B6. Western blot analysis showing decreased phosphorylation of FRS2 in CSC 85 cells after ch3B6 treatment in the absence or presence of the indicated FGFs compared to untreated or PBS controls. pFRS2 and GAPDH bands were quantified, and ratios were calculated and expressed as FC, arbitrarily considering samples “C” as 1. (**A**) Assay run in the absence of heparin. (**B**) Assay run in the presence of heparin. GAPDH served as a loading control. C, complete medium; S, starvation medium. For each experiment marked by a black box, C and S samples were run and presented; the gels were spliced to place ligand-treated groups side by side, with samples treated with the same ligand grouped together and separated from other ligand groups by a white space. (**C**) Schematic summary of Western blots shown in panels (**A**,**B**). FGFR4 pathway induction and inhibition by ch3B6 antibody are schematically represented with a “+” and a “–”, respectively, and summarized with the following symbols: for “no heparin” condition (panel (**A**)): induction * (− = no induction; + = 0–2x; ++ = 2–5x; +++ = 5–10x); inhibition ** (− = 0–30%; − − = 30–60%; + = induction); for “heparin” condition (panel B): ligand induction ^ (+ = 0–2x; ++ = 2–5x; +++ = 5–10x); ^^ (+ = 0–2x; ++ = 2–5x; +++ = 5–10x); ^^^ (− = 0–30%; − − = 30–60%; − − − = 60–100%). The uncropped blots are shown in File S1.

To further validate the specificity of ch3B6’s antiproliferative effects, we examined its ability to inhibit FGFR4-mediated signaling by measuring FRS2 phosphorylation (pFRS2), a key downstream event, in the #85 colorectal CSC line (Figure 9).

Under spherogenic conditions supplemented with EGF, bFGF, and heparin (complete medium, see “C” samples), CSCs exhibited strong FRS2 phosphorylation (Figure 9, “C” lanes). In contrast, when cultured in growth factors/heparin-depleted medium (starvation conditions, indicated as “S” samples), pFRS2 levels were much lower or nearly undetectable. Unlike in HCC cells, where FGFR4 signaling is typically driven by FGF19 (Figure 5B), colorectal CSCs did not respond well to FGF19 under starvation conditions—even when the ligand was supplied at concentrations 10 times higher than that of bFGF in complete medium (Figure 9A,B).

To explore alternative FGFR4-activating cues, we tested a panel of high-affinity FGFR4 ligands—FGF1, FGF2 (bFGF), FGF4, FGF6, FGF8 (a and b), FGF17, FGF18—with or without heparin. In the absence of heparin (Figure 9A), all ligands induced FRS2 phosphorylation to varying extents, with FGF19 producing the weakest response (compare “PBS” treated samples with “S” samples). Among all ligand-induced activation scenarios, ch3B6 reduced pFRS2 levels in the case of the FGF8a, FGF8b, FGF17, and FGF18 ligands, although the extent of inhibition varied by ligand.

When cells were co-treated with heparin and each ligand (Figure 9B), with bFGF, FGF1, FGF4, and FGF6, the presence of heparin did not enhance pFRS2 levels. However, when co-administered with heparin, FGF8a, FGF8b, FGF17, FGF18, and—more modestly—FGF19 robustly increased pFRS2 levels beyond the level of induction obtained with the ligand alone (Figure 9B, compare PBS—heparin vs. PBS + heparin). In all these latter cases, ch3B6 effectively blocked the ligand-induced phosphorylation.

These findings confirm that in colorectal CSCs, FGFR4 activation occurs through a broader and likely different range of FGF ligands than in HCC, and that ch3B6 can disrupt this signaling axis.

### 3.11. In Vivo ch3B6 Anti-Tumor Efficacy on Colorectal CSC-Derived Xenograft Models

We analyzed the effect of the ch3B6 antibody on subcutaneous tumors generated by inoculating colorectal CSCs into immunodeficient mice. To this aim, #85 CSCs were injected into the subcutaneous tissue of NOD/SCID mice, and three weeks after transplantation, mice that developed measurable tumors were randomized and assigned to two groups (n = 9) with a mean tumor volume of 65 mm^3^ to be treated with the ch3B6 antibody or vehicle (PBS), respectively. Animals were treated via intraperitoneal injection with 25 mg/kg of ch3B6 in PBS, administered twice a week for two weeks. Mice in the control group were injected with corresponding volumes of PBS.

**Figure 10:**
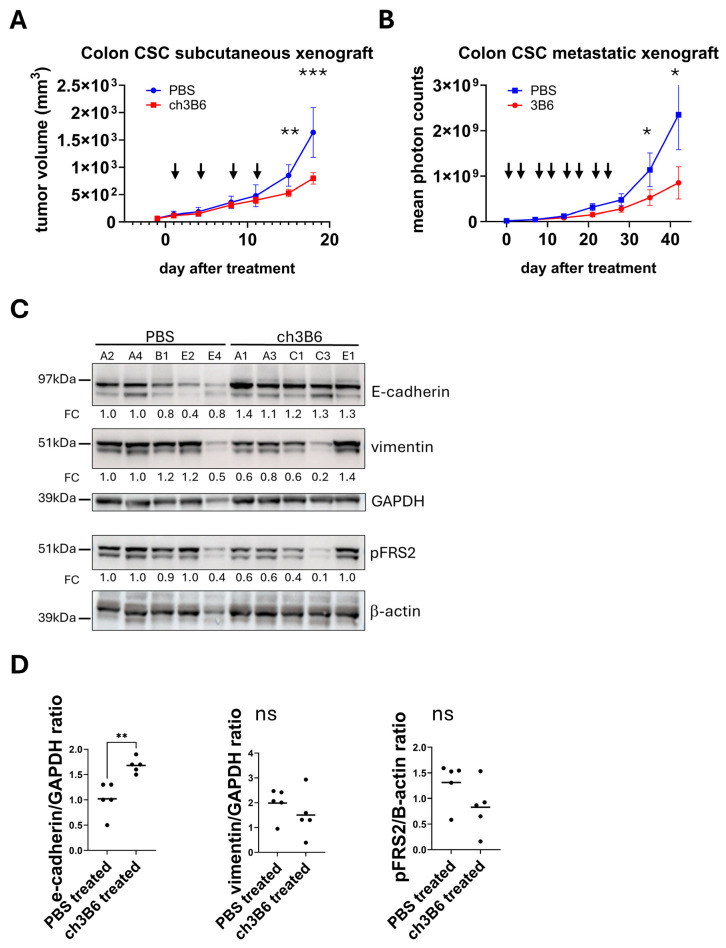
Therapeutic potential of ch3B6 in CSC xenografts. (**A**) Tumor volume reduction in CSC 85-derived subcutaneous xenografts after ch3B6 treatment. (**B**) Bioluminescence monitoring hepatic metastases in mice injected with luciferase-labeled CSCs and treated with ch3B6 or control. For both panels (**A**,**B**) arrows represent treatment administrations. (**C**) Total SDS protein extracts from tumor samples of the vehicle (PBS, samples # A2, A4, B1, E2, E4) and ch3B6 antibody (ch3B6, samples # A1, A3, C1, C3, E1) treatment groups were subjected to Western blot analysis using anti-vimentin and anti-e-cadherin antibodies together with an anti-GAPDH antibody as normalizer (upper gel), or with an anti-phospho-FRS2 antibody and an anti-b-actin antibody as normalizer (lower gel). Bands were quantified and ratios to the indicated normalizers were calculated and expressed as FC, arbitrarily considering sample “A2” as 1. (**D**) Bands visualized in the two gels were quantified with the Vilber Fusion FX Spectra system and normalized vs. GAPDH (e-cadherin and vimentin) or Beta-actin (pFRS2); the ratio in content was plotted with GraphPad. The uncropped blots are shown in File S1. ns: *p* > 0.05; * *p* < 0.05; ** *p* < 0.01; *** *p* < 0.001.

In mice treated with the ch3B6 antibody, tumor growth was reduced compared to control mice, with a statistically significant difference observed starting on day 16 of treatment (Figure 10A). No body weight loss or clinical adverse events were observed in treated mice (Supplementary Figure S9).

At the end of the four weeks treatment, residual tumors were explanted and homogenized for Western blot analysis of total proteins. As shown in Figure 10C,D, ch3B6 induced a decrease in the phosphorylation of the first downstream marker of FGFR4, i.e., FRS2 protein, indicative of downregulation of the pathway. More interestingly, treatment with ch3B6 antibody induced an increase in E-cadherin and in parallel a decrease in vimentin content, sustaining the role of FGFR4 in regulating the EMT process in CRC and demonstrating the ability of the ch3B6 antibody to inhibit FGFR4-induced EMT in vivo.

Then we assayed the effect of ch3B6 on hepatic metastases generated by injection of colorectal CSCs in the liver of immunodeficient mice. To this aim a reporter # 85 LUC-GFP cell line was used. This line was generated by transducing #85 CSCs with a lentiviral vector encoding both the fluorescent protein GFP and luciferase. #85-LUC-GFP cells were injected into the liver parenchyma of each of twenty NOD/SCID mice, and tumor growth was monitored by bioluminescence imaging of luciferase-labeled tumor cells. Ten days after transplantation, mice were randomly assigned to two groups (n = 7) and treated, respectively, with ch3B6 or PBS (control) twice a week for four weeks via intraperitoneal injection with 25 mg/ Kg of antibody, or PBS. Mice were then monitored for an additional two weeks after the end of treatment prior to sacrifice. Compared to vehicle-treated controls, ch3B6 was able to reduce the growth rate of the liver tumor metastases (Figure 10B). Notably, the anti-tumor effect shown by ch3B6 persisted after the end of treatment, reaching statistical significance starting from the seventh day of the observation period. Also in this model, no negative effects on body weight or pathological signs were observed in treated mice (Supplementary Figure S9).

## 4. Discussion

The cancer stem cell (CSC) concept was developed about 40 years ago and posits that, in analogy to the renewal of healthy tissues, cancer growth is fueled by cell populations with stem-like characteristics that are responsible for relapse, metastatic spread, and therapy resistance [7]. While CSCs have been identified in many hematologic and solid tumors, it became evident that CSC hierarchies are not rigid and that CSCs and non-CSCs may interconvert in a highly plastic way, often as a result of environmental stimuli [12]. Stemness characteristics have been confirmed to track with tumor aggressiveness, justifying the identification of druggable targets in CSCs, with the caveat that exclusive targeting of CSCs may lead to clinical benefit only in combination settings due to the aforementioned cellular plasticity. CRC is considered a prototype of a cancer stem cell (CSC)-driven tumor, but only a limited number of potential colorectal CSC druggable targets have been identified to date against which in only a few cases therapeutic drugs have been described, none of them being monoclonal antibodies [75,76,77,78,79,80,81].

The availability of a large collection of colorectal CSC lines and the access to genome-wide expression data obtained on a significant number of these cells had offered us the opportunity for the generation of specific antibodies against newly identified membrane proteins enriched in undifferentiated colorectal CSC cultured as spheroids. Using a combination of bioinformatic and experimental methodologies, FGFR4 was selected as a preferred potential target for the development of a therapeutic monoclonal antibody. We found that this protein has a major role in driving several important functions of colorectal CSCs linked to their migration, survival, proliferation, and in vivo tumorigenicity. We generated monoclonal antibodies against this antigen and selected one of them, namely 3B6, a high-affinity and highly specific FGFR4 binder that inhibits these CSC properties in vitro and shows in vivo anti-tumor activity in CRC animal models induced with patient-derived CSCs.

Interestingly, we found that the 3B6 antibody exerted anti-proliferative and anti-tumor activities both on RAS wt (#CRO-1) and RAS-mutated (#85) colorectal CSCs [66]. RAS-mutated colon CSC were shown to be resistant to anti-EGFR agents such as cetuximab, mirroring the lack of response to this agent by RAS-mutated metastatic CRC patients [67]. Clinically, 3B6 could represent a promising therapeutic option for patients with mutated RAS tumors who represent approximately 50% of all metastatic CRC patients, potentially also in combination with the emerging RAS inhibitors and an additional targeted-therapy agent that in combination with cetuximab could help to achieve the optimal response to anti-EGFR therapy. Since FGFR4 expression is higher in MSS colon cancers, which represent the vast majority of the cases, 3B6 could be a therapeutic option where also immune checkpoint inhibitors are not indicated.

Canonical signaling via FGFR4 is dependent on receptor dimerization upon FGF ligand binding that leads to autophosphorylation of tyrosine residues in the cytoplasmic receptor domain. These act as binding sites for signaling proteins and docking proteins, which form a complex with an additional complement of signaling proteins [45]. Although human FGF19 and its mouse orthologue FGF15 are the FGFs that most specifically signal through FGFR4, predominantly via the beta-klotho/FGFR4 complex, in cells lacking beta-klotho expression FGFR4 was previously shown to be effectively activated by a broader albeit specific subset of different ligands of the FGF family including FGF1, 2, 4, 6, 8, 17, and 18 [82]. Similarly to other FGFRs, FGF binding is enhanced by the formation of quaternary complexes including heparin or heparan sulfate proteoglycans (HSPG), which is instrumental for ligand-induced receptor activation [83].

Different from HCC, beta-klotho expression is downregulated in colon cancer tissues and in the colorectal CSCs used in the present study (TCGA and proprietary Affymetrix data). Consistent with these data, unlike in HCC cells in which the FGFR4 pathway is induced by the treatment with FGF19, this ligand did not induce FRS2 phosphorylation in representative colorectal CSC lines used in the present study.

A specific FGF subset including FGF1, 2, 4, 6, 8, 17, and 18 were instead able to induce FRS2 phosphorylation in the same representative CSC lines in agreement with previous evidence in cells lacking beta-klotho expression [82]. As expected, heparin addition increased activation levels of some of these ligands, among which FGF8a, 8b, 17, and 18 were described in previous studies to play a functional role in colon cancer cell proliferation, tumorigenesis, and metastatic potential either through autocrine or paracrine, CAF-mediated mechanisms [83,84,85,86,87]. Interestingly, in CRC patients both FGFR4 and FGF18 expression are predictors of poor prognosis, and a positive correlation between FGF18 and FGFR4 expression was reported, underlining the possible clinical relevance of the FGF18-FGFR4 axis in this patient population [88]. Most notably, now we showed that in the case of the mentioned FGF8a, 8b, 17, and 18 ligands, and to a lesser extent of FGF19, the induced FRS2 phosphorylation could be decreased by our FGFR4-specific 3B6 antibody, highlighting the activation role of these specific ligands in CRC CSCs and the therapeutic potential of our antibody in this tumor type. These findings are in partial disagreement with previously published data showing that in colon cancer xenograft models blockade of FGF19 by an antibody reduced in vivo tumor growth, arguing for the existence of an autocrine FGF19/FGFR4 oncogenic signal loop [89]. However, in the cited paper, commercial colon cancer cell lines were used that have the peculiarity of expressing endogenous FGF19, and no in vitro experiments were presented to show pFRS2 modulation or cell proliferation effects, thus making it difficult to compare results. More recent studies, also based on FGF19-positive or ELF4-positive colon cancer cell lines, have highlighted a role of FGF19 in colon liver metastasis formation in a microenvironment-dependent manner [90,91]. It would be interesting to confirm these findings in colorectal CSC-based liver metastatic animal models in which we demonstrated an anti-tumor effect by 3B6. Of relevance are also recent published data showing that the FGF2/FGFR4 axis mediates a rescue response upon treatment of colon CSCs with BCL-XL inhibitors, thus inducing apoptosis resistance [92]. On this basis, it would be interesting to test whether 3B6 in combination with BH3 mimetics can sensitize colon CSCs to apoptosis inducing drugs.

By deletion mutagenesis and peptide epitope mapping we have identified, as the 3B6 specific FGFR4 binding site, the linker region between the Ig-I and Ig-II domains, named “acid box” for its high content of acidic amino acid residues. Based on biological evidence and structural NMR studies, it has been proposed that the “acid box” can participate in the negative regulation of FGF binding to FGFR in that the multiple acidic residues of this region can mimic the negative potential surface of heparan sulfate and bind at the heparin-binding site located on the surface of FGFR Ig-II region, thus locking the receptor in a “closed” conformation and preventing initiating a biological response [93]. On this basis, it is tempting to hypothesize a possible 3B6 mechanism of action consisting in the stabilization of the receptor in the “closed”, auto-inhibitory conformation. Such an allosteric inhibition mechanism was not previously described for other neutralizing anti-FGFR4 antibodies that instead directly compete for FGF binding to the FGFR4 Ig-II domain. In tumors like colon cancer, which do not express beta-klotho, where heparan sulfate is an important co-activator, an antibody that blocks the heparan sulfate binding site, thus preventing the interaction with FGF, could have an advantage over antibodies that exclusively block the interaction with the ligand alone. Future efforts will be made to solve the structure of the 3B6-FGFR4 ECD complex by cryo-electron microscopy.

A CSCs–immune cells crosstalk is well documented in different tumor types, including colon cancer which creates an immunosuppressive tumor microenvironment that reshapes the stemness in tumor cells, resulting in tumor formation and progression [94]. It is also well known that the effects of anti-tumor cell antibodies can be potentiated if they are used in combination with antibodies blocking immune-checkpoints inhibitory signals. To overcome CSC immune evasion, it would be of interest to develop new therapeutic strategies based on a combination of anti-CSC antibodies and immune-oncology inhibitors. In a recent publication [95], it was demonstrated that lenvatinib, a multi-RTK inhibitor approved for the first-line treatment of patients with unresectable HCC, in addition to its antitumor activity, can improve the efficacy of an antibody anti-PD-1 by blocking specific FGFR4-dependent immuno-oncology pathways in tumor-infiltrating T regulatory cells. These findings pave the way to explore potential 3B6 and anti-PD-1 antibody combination treatments not only in HCC, but also in colon CSC models of primary tumors and liver tumor metastasis.

In preclinical studies small molecule FGFR4 TK inhibitors, when used as single agents, showed anti-tumor effects in colon carcinoma models at concentrations 100-fold higher than those efficacious in HCC cells [92,96,97]. However, we observed inhibitory activity by the FGFR4 TK inhibitor BLU9931 both on the viability and the colony-forming efficiency of the tested colorectal CSCs at concentrations comparable to those affecting HCC cell viability maybe due to different experimental conditions, more sensitive assays, or CSC-specific effects. Notably, 3B6 was an equally potent inhibitor in the same assays. FGFR4 TK inhibitors have shown preliminary efficacy in recent clinical trials for HCC patients with FGF19 overexpression. However, the observed responses only lasted a few months before tumors relapse [58]. Acquired FGFR4-resistant mutations were found in ~30% of FGFR4 inhibitor-responsive patients [98]. Similar FGFR4 mutations have also been found de novo in about 7–10% of Rhabdomyosarcoma (RMS) and ER-treated invasive lobular carcinoma patients [99,100]. FGFR4 inhibitors have minimal activity against these de novo or acquired resistant mutations [98,101]. Therefore, targeting FGFR4 with a therapeutic antibody could help overcome resistance and offer alternative, effective, and more durable treatment options.

## 5. Conclusions

This study successfully identified and validated several membrane proteins specifically expressed in colorectal CSCs, with FGFR4 standing out as a particularly promising therapeutic target. In response, the authors developed monoclonal antibodies against FGFR4, one of which stands out for the peculiar binding site and very high affinity for the receptor, as well as for the efficacy in both in vitro and in vivo assays. The 3B6 antibody therefore shows potential not only for improving the diagnosis and treatment of CRC, but also for providing a more targeted approach in eradicating CSCs. These findings open new avenues for personalized therapies in those cancers characterized by FGFR4 expression, aiming to enhance treatment efficacy and reduce the likelihood of cancer recurrence.

## 6. Patents

There is a patent derived from this work and it is US11643467B2.

## Figures and Tables

**Table 1:** EC_50_ measurement of the eight anti-FGFR4 mAbs on three colorectal CSCs and HCC Huh7 cells.

Cells	Parameter	5B5-G7	1E7-C4	3B6-E4	5H9-D1	6C9-C11	8D4-E2	8E3-E4B	10G11-F3
CSC 1.2	Bmax	5.6	4.9	4.5	7.5	4.7	5.1	4.5	3.2
	EC_50_ (mg/mL)	0.18	0.2	0.2	1.3	0.14	0.78	1.1	0.03
	EC_50_ (nM)	1.2	1.3	1.3	8.7	0.9	5.2	7.3	0.2
CSC 85	Bmax	4.6	6.0	3.9	7.7	3.9	4.6	8.4	4.0
	EC_50_ (mg/mL)	0.07	0.55	0.14	1.2	0.05	0.5	2.7	0.04
	EC_50_ (nM)	0.45	3.7	0.9	8.0	0.3	3.3	18.0	0.27
CSC CRO1	Bmax	5.0	6.6	4.46	7.9	4.3	5.0	9.6	2.4
	EC_50_ (mg/mL)	0.07	0.57	0.16	1.0	0.05	0.5	3.2	0.03
	EC_50_ (nM)	0.5	3.8	1.1	6.7	0.3	3.3	21.3	0.2
Huh7	Bmax	63.5	34.3	27.5	21.1	30.6	33.4	NC	8.3
	EC_50_ (mg/mL)	0.16	0.49	0.26	2.36	0.12	1.44		0.06
	EC_50_ (nM)	1.1	3.3	1.7	15.7	0.8	9.6		0.4 ^1^

^1^ FC of MFI values with respect to the control (secondary antibody only) were plotted as a function of the mAb concentration; Bmax and EC_50_ were calculated using the GraphPad Prism software and applying the “one site binding equation”. NC: not converge.

**Table 2:** IC_50_ values of the eight anti-FGFR4 mAbs in a solid-phase FGF19-FGFR4 binding inhibition assay.

mAb	IC_50_ (mg/mL)	IC_50_ (nM) ^1^
5B5-G7	0.2	1.7
1E7-C4	0.1	0.7
3B6-E4	0.2	1.1
5H9-D1	0.2	1.1
6C9-C11	0.3	1.7
8D4-E2	0.3	1.7
8E3-E4	0.8	5.5
10G11-F3	0.1	0.9

^1^ IC_50_ values were calculated by 4P logistic fitting of the experimental data with the KaleidaGraph 3.52 software based on the residual absorbance at 450 nm (% of PBS control) at increasing antibody concentrations (1:3 serial dilutions from 0.014 to 30 mg/mL; n = 3).

## Data Availability

The original Affymetrix gene expression dataset from colorectal CSC lines analyzed during the current study is available from the corresponding author upon reasonable request. All data generated and analyzed from it during this study are included in this published article and its Supplementary Information Files.

## Supplementary Materials

The following supporting information can be downloaded at https://www.mdpi.com/article/10.3390/cancers18030418/s1, Figure S1: Datasets within the public NCBI GEO database included in the analysis; Figure S2: Principal component analysis (PCA) of the data in the normal and tumor colon datasets; Figure S3: Expression of the five selected target genes in cancer patient samples; Figure S4: FGFR4 expression relative to GAPDH expression measured by RT-qPCR in different CRC CSC lines cultured with or without bFGF in the standard CSC medium; Figure S5: FACS analysis of BOSC23 overexpressing the five final targets and immunoreactivity of rat sera; Figure S6: Summary of FACS data and FGF19 ELISA competion assays obtained with the 70 supernatants on 5 different CSC lines; Figure S7: Characterization of the eight selected anti-FGFR4 rat antibodies; Figure S8: Quality controls of the antibody ch3B6 purification. Figure S9: Body weight changes during in vivo efficacy studies. File S1: Uncropped gels images. Table S1: Bionformatic ID of CSC-enriched genes (ComBat datasets list, 250 selected genes list, 103 selected genes list, 56 CSC target Score list, 36 CSC targets Score list, 36 genes relative expression list, Final 21 Targets list); Table S2: List of RNAs from cell lines used for experimental validation of selected targets; Table S3: Differential expression of the top 21 candidate genes in CSC vs. other cells.

## Abbreviations

The following abbreviations are used in this manuscript:

CRC	Colorectal cancer
CSC	Cancer stem cell
FGFR	Fibroblast growth factor receptor
EGF	Epidermal Groth Factor
PD-1	Programmed cell death protein 1
HCC	Hepatocellular carcinoma
DEGs	differentially expressed genes
LCM	Laser capture microdissection
ECD	Extracellular domain
IL17RB	Interleukin-17 receptor B
RGMB	Repulsive guidance molecule B
EDAR	Ectodysplasin A receptor
OR51E1	Olfactory receptor family 51 subfamily E member 1
COAD	Colon adenocarcinoma
MSI	Microsatellite instability
MSS	Microsatellite stable
CYP7A1	Cholesterol 7a-hydroxylase A1
CTGF	Connective tissue growth factor
AB	Acid box
FRS2	Fibroblast growth factor receptor substrate 2
HSPG	Heparan sulfate proteoglycans
TK	Tyrosine kinase
EMT	Epithelial-to-mesenchymal transition
ELF4	E74 Like ETS Transcription Factor 4
RMS	Rhabdomyosarcoma
