# Supplementary material for: Targeting Colorectal Cancer Stem Cells Through Inhibition of the Fibroblast Growth Factor Receptor 4 Pathway with a Novel Antibody
Source: Cancers (Basel). 2026 Jan 28;18(3):418. doi: 10.3390/cancers18030418 (PMC12896886; doi:10.3390/cancers18030418)

Supplementary File S1: uncropped western blot gels

Figure 3E

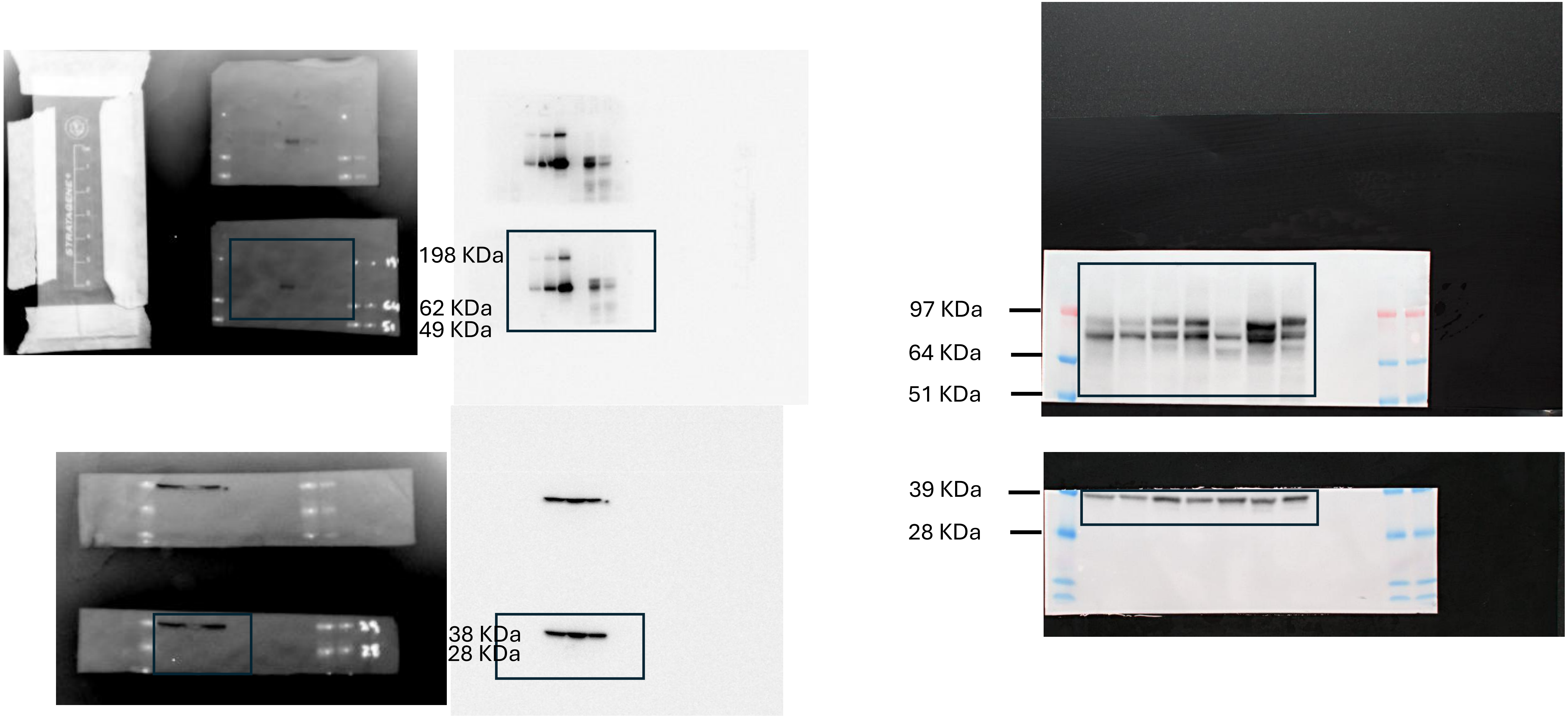

Figure 5B

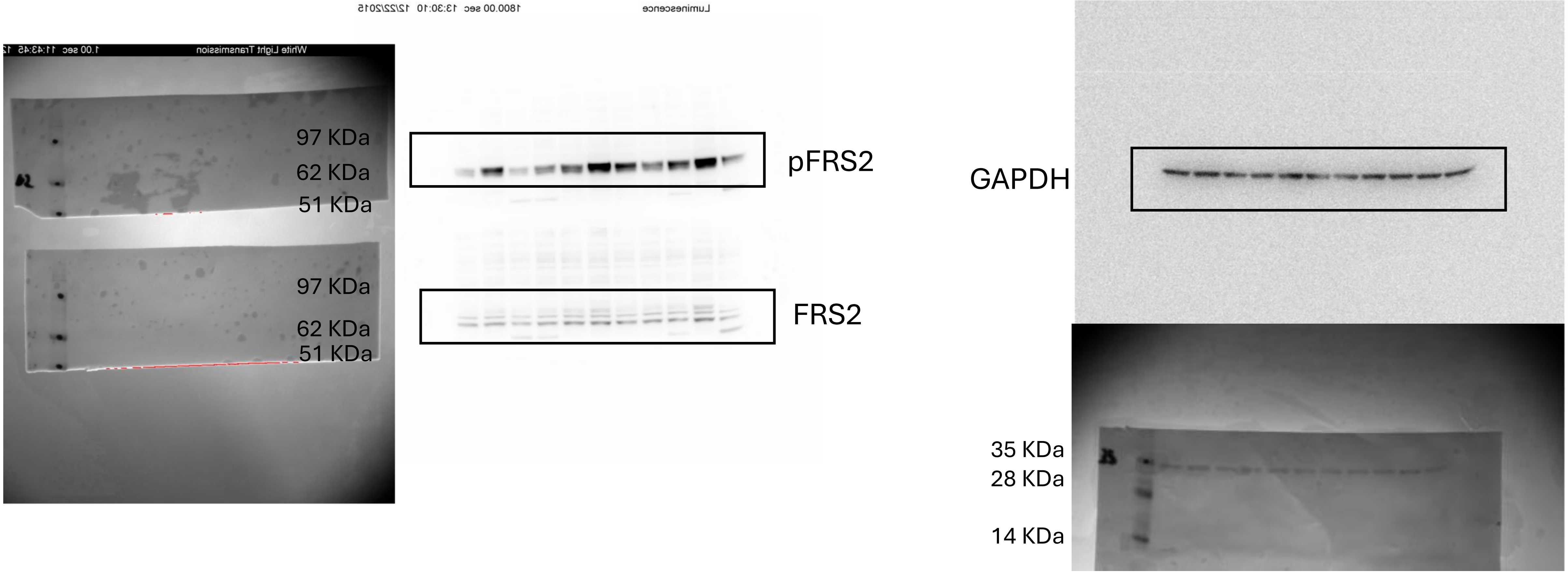
97 KDa
62 KDa
51 KDa
97 KDa
62 KDa
51 KDa
pFRS2
FRS2
GAPDH
35 KDa
28 KDa
14 KDa

Figure 9A No heparin

FGF1

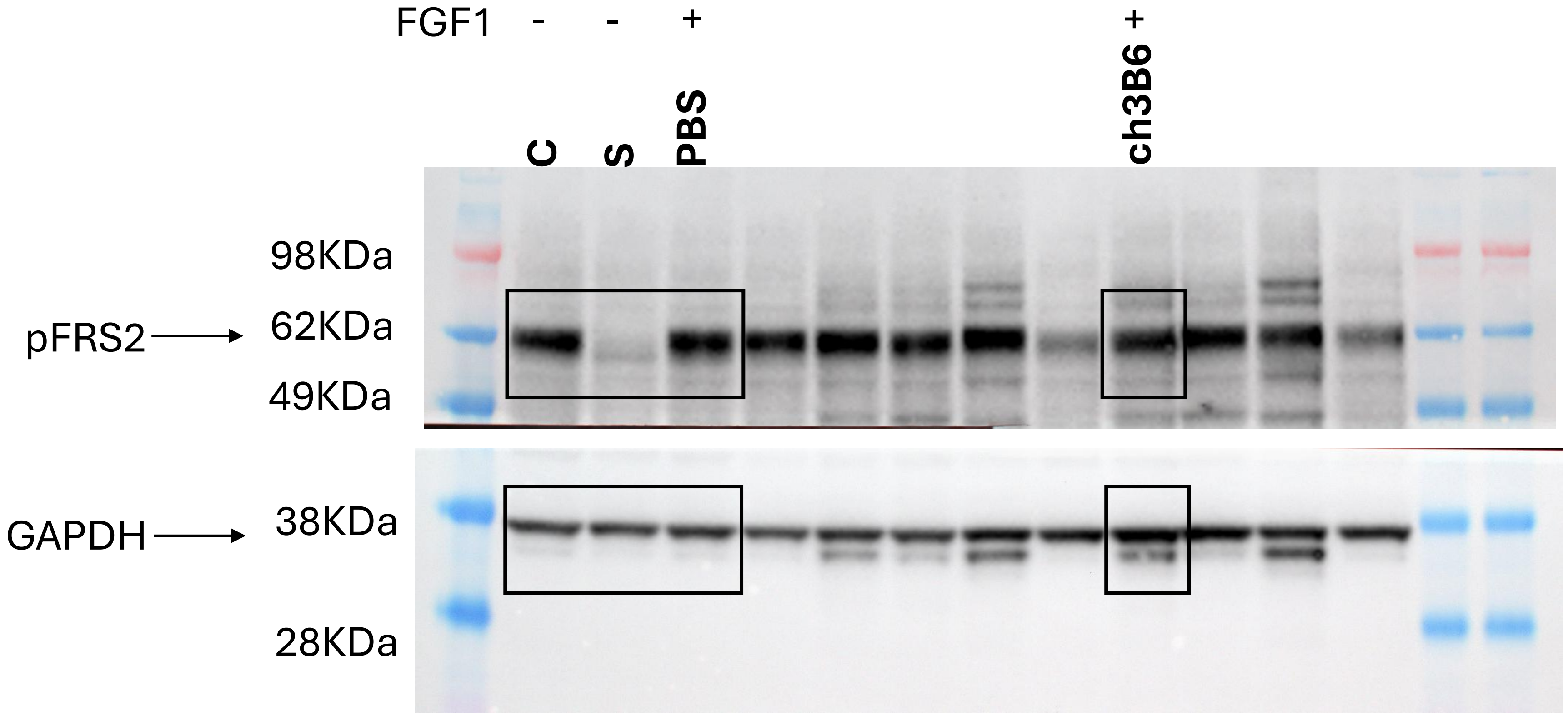

Figure 9A No heparin

bFGF, FGF4, FGF6

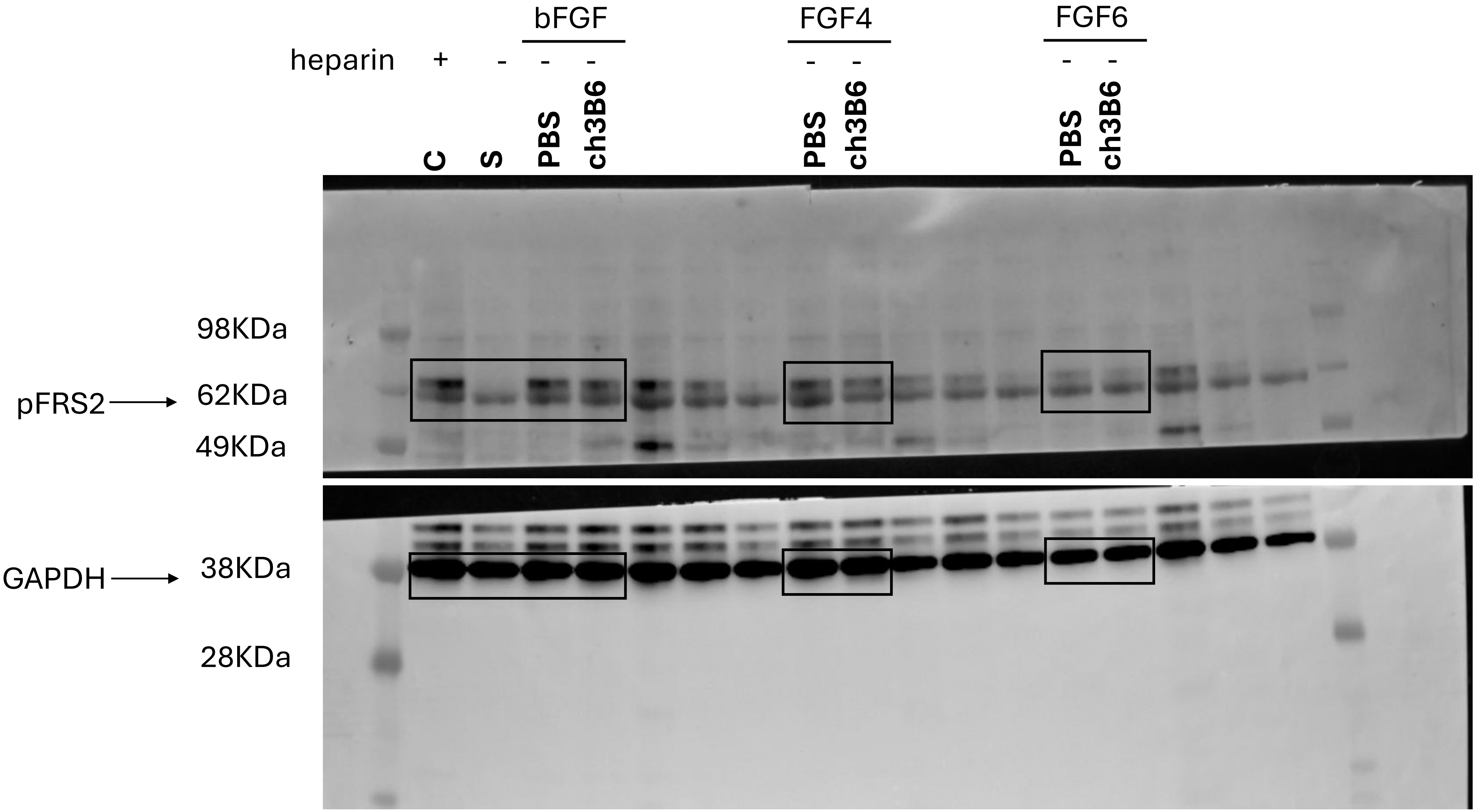

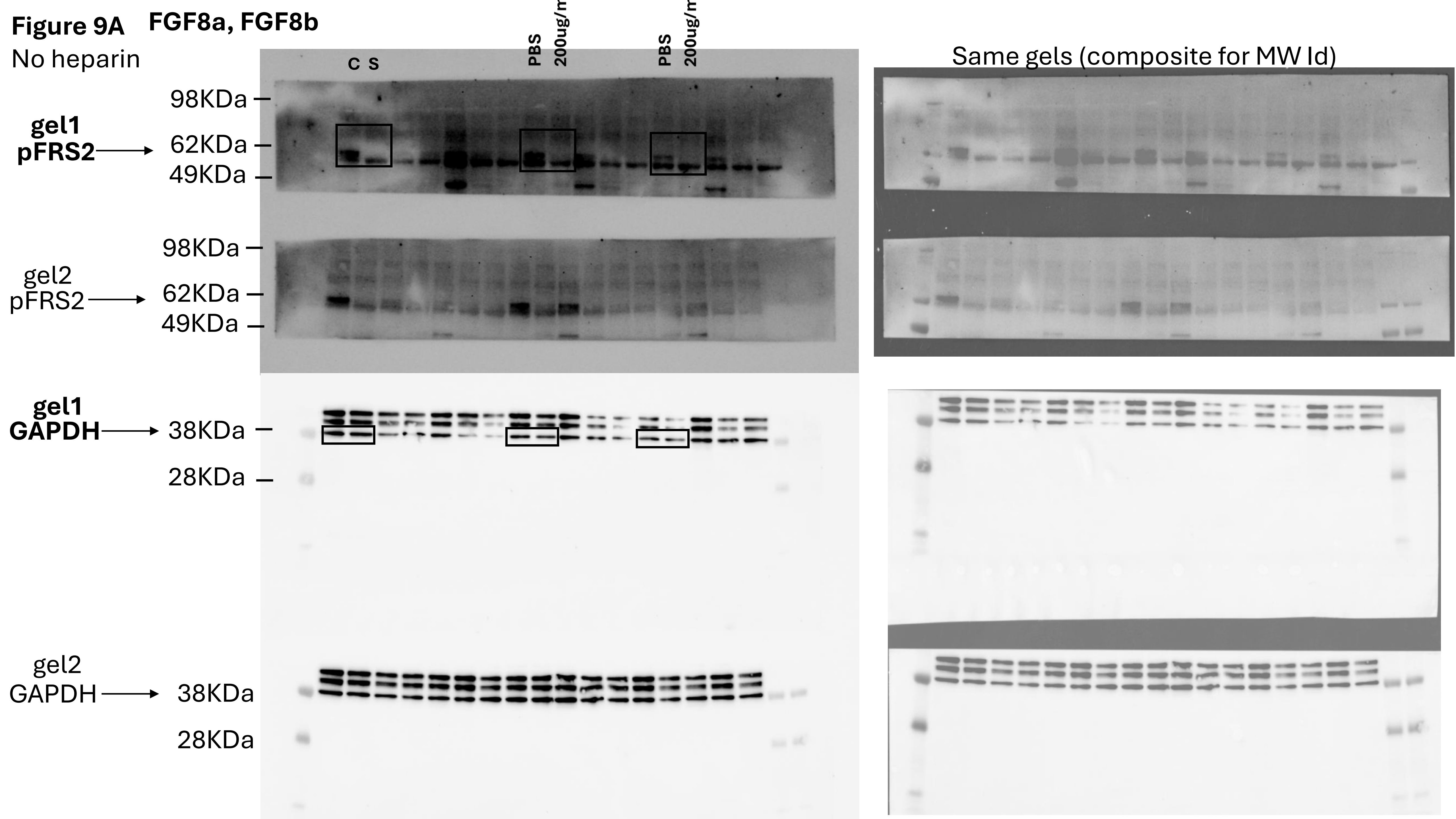

Figure 9A
No heparin

Figure 9A **FGF17. FGF18, FGF19**

No heparin

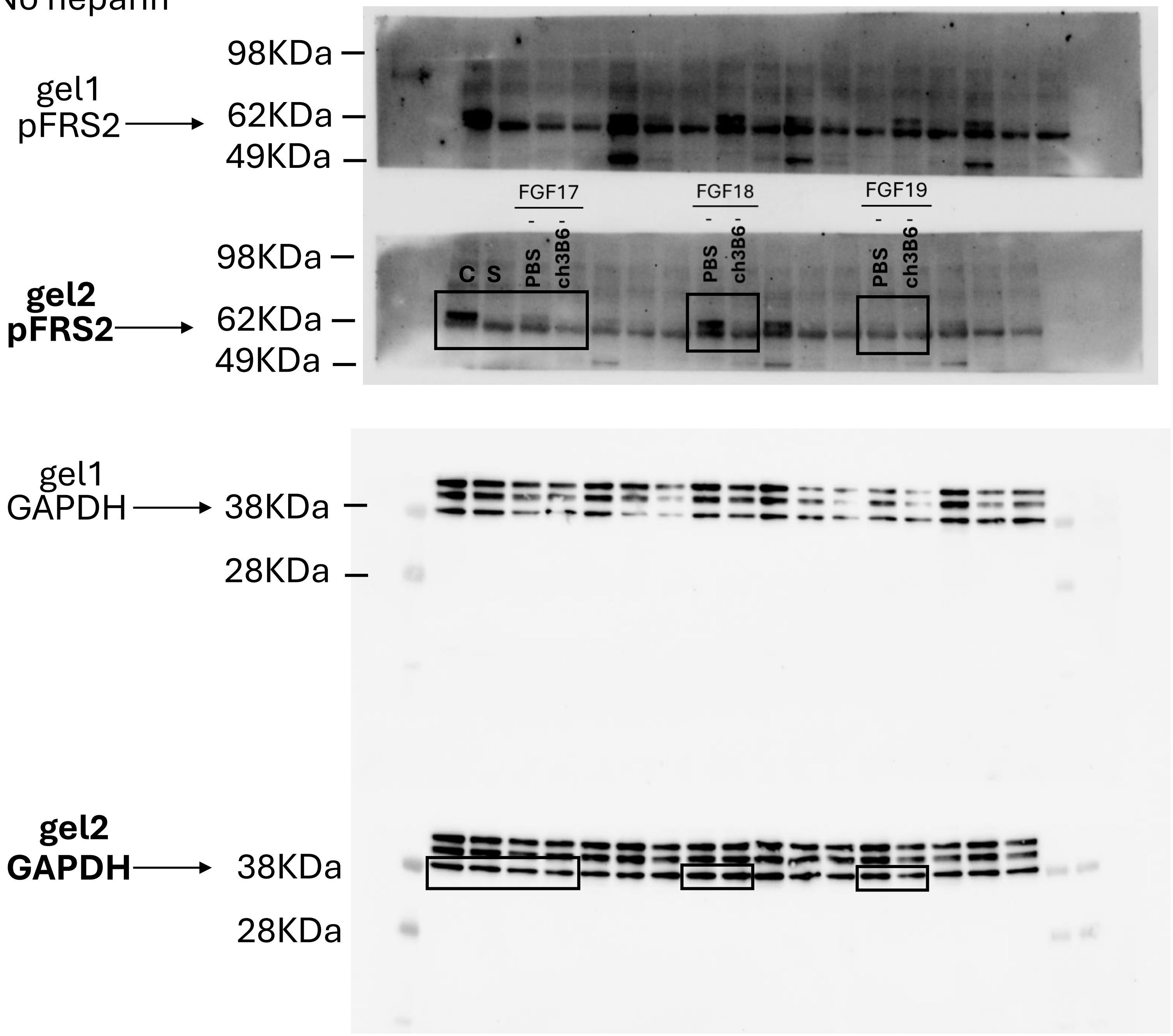

Same gels (composite for MW Id)

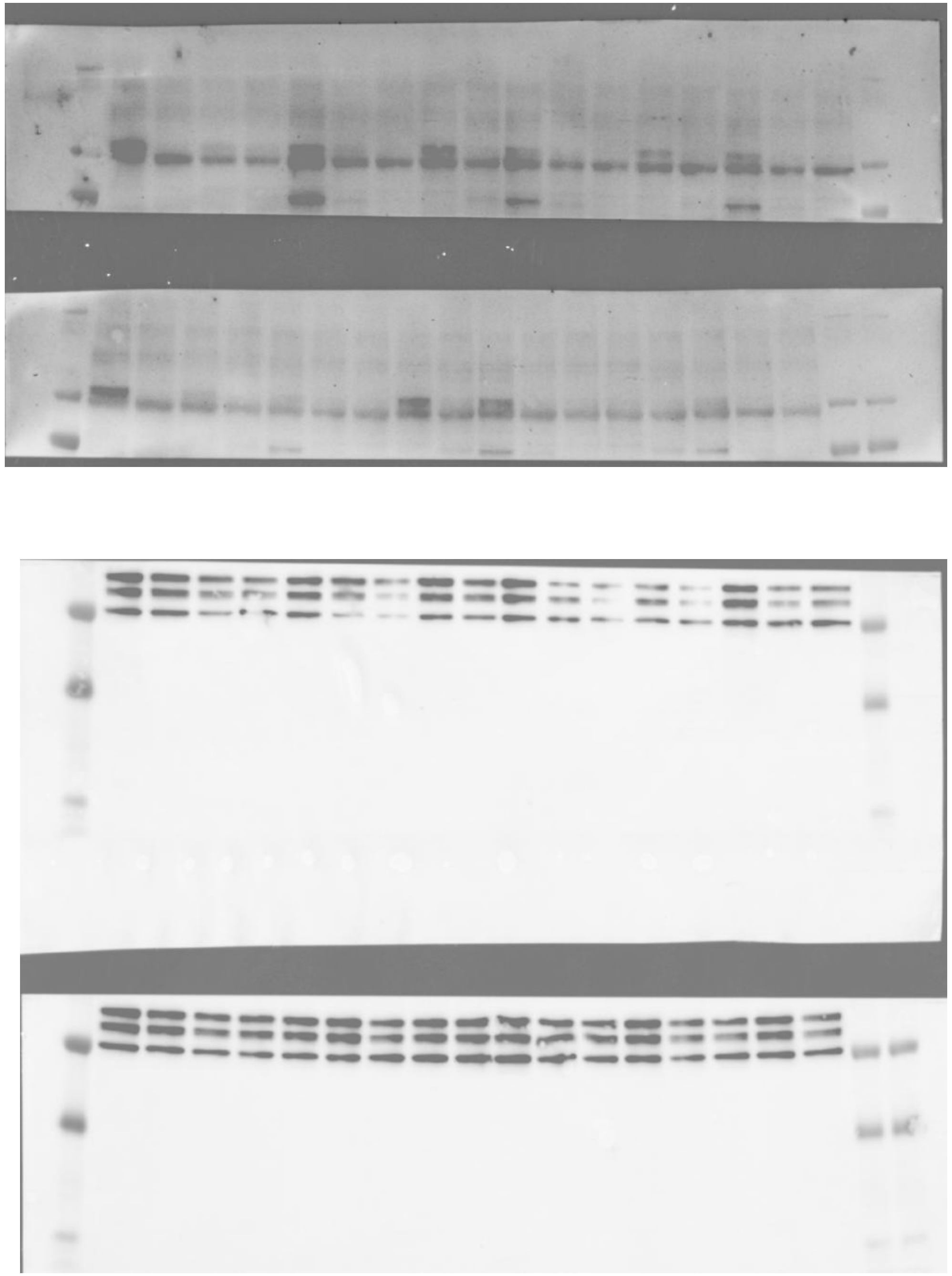

Figure 9B Heparin

bFGF, FGF1, FGF4

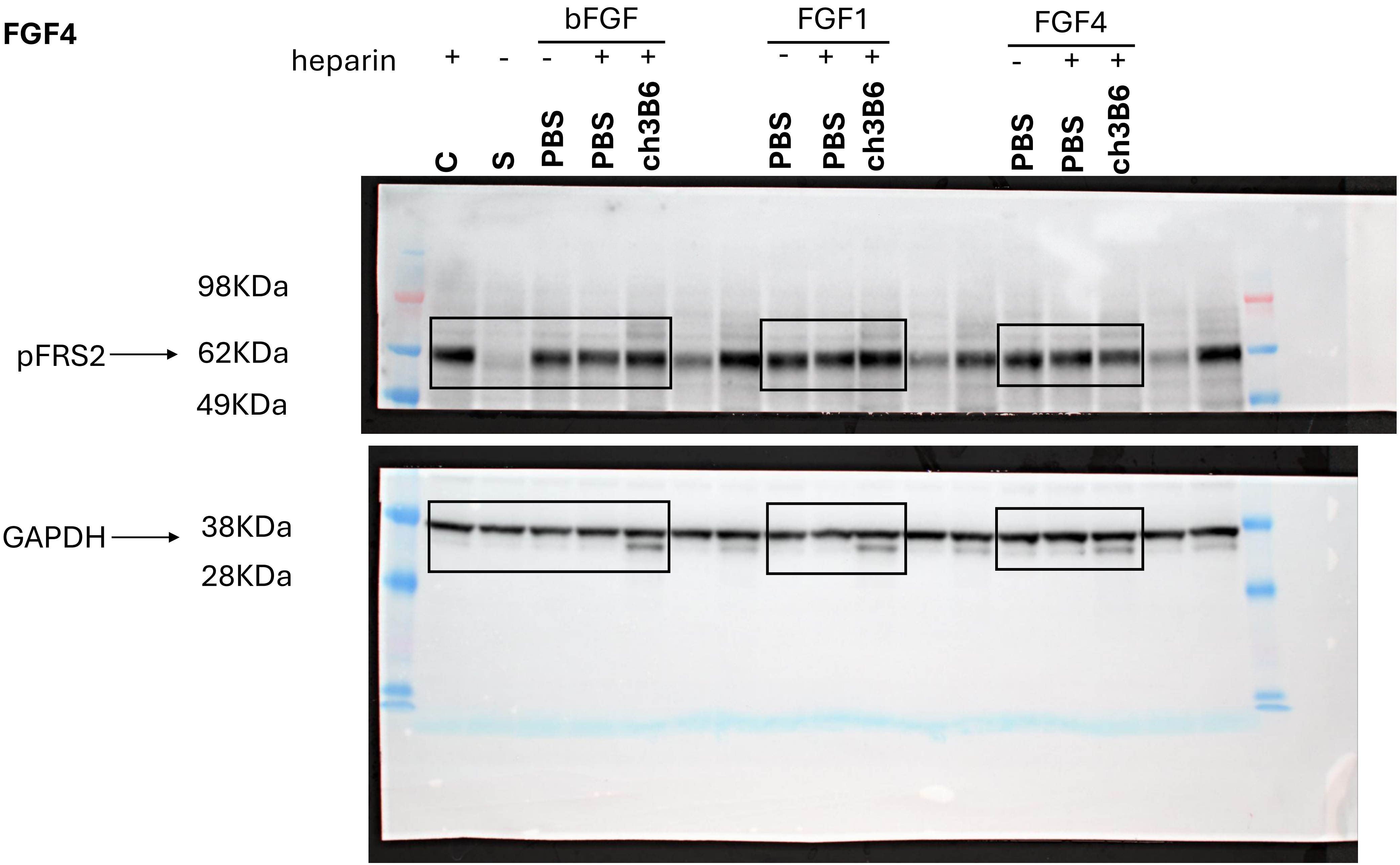

Figure 9B Heparin

FGF6, FGF8a, FGF8b

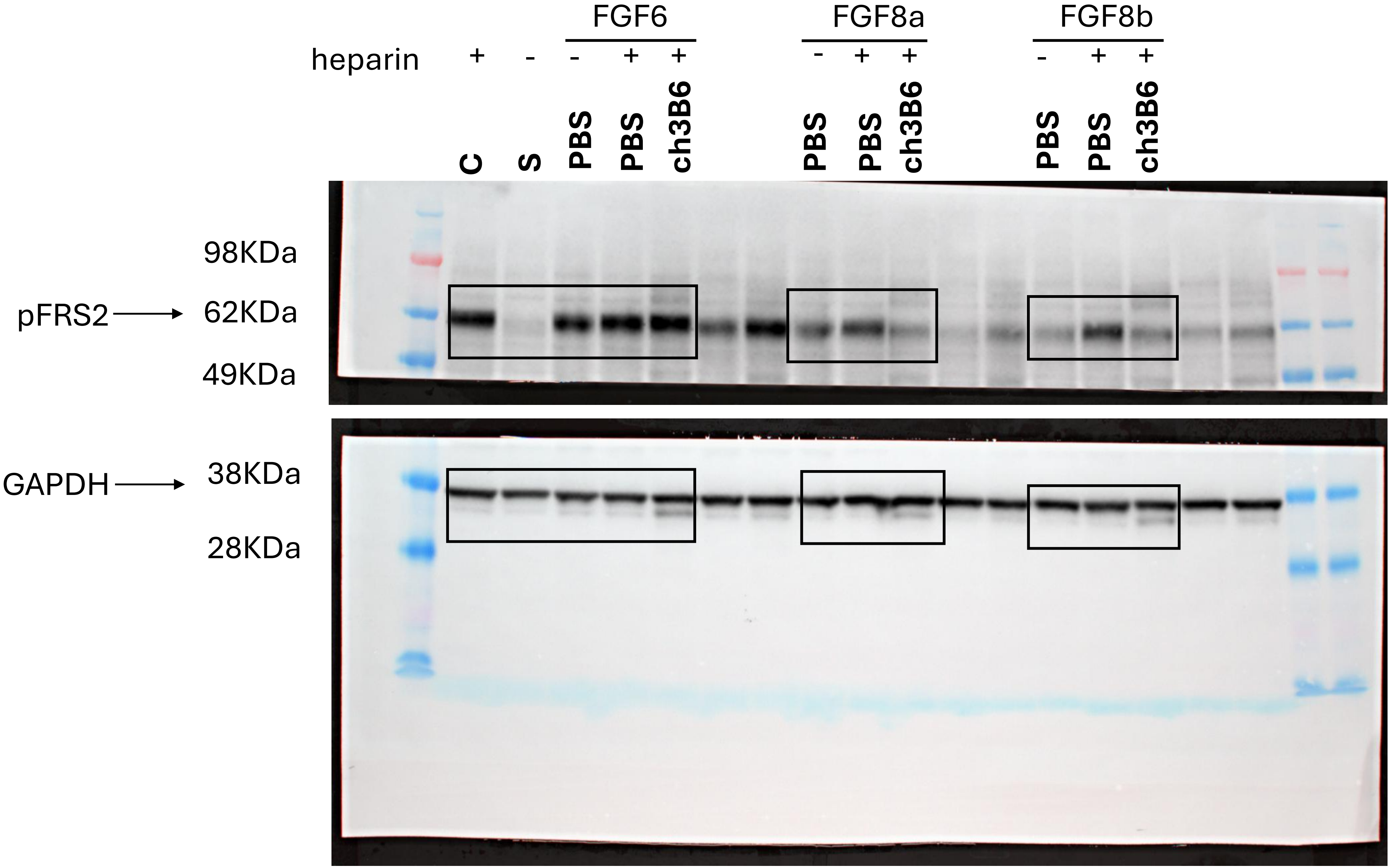

Figure 9B Heparin

FGF17, FGF18, FGF19

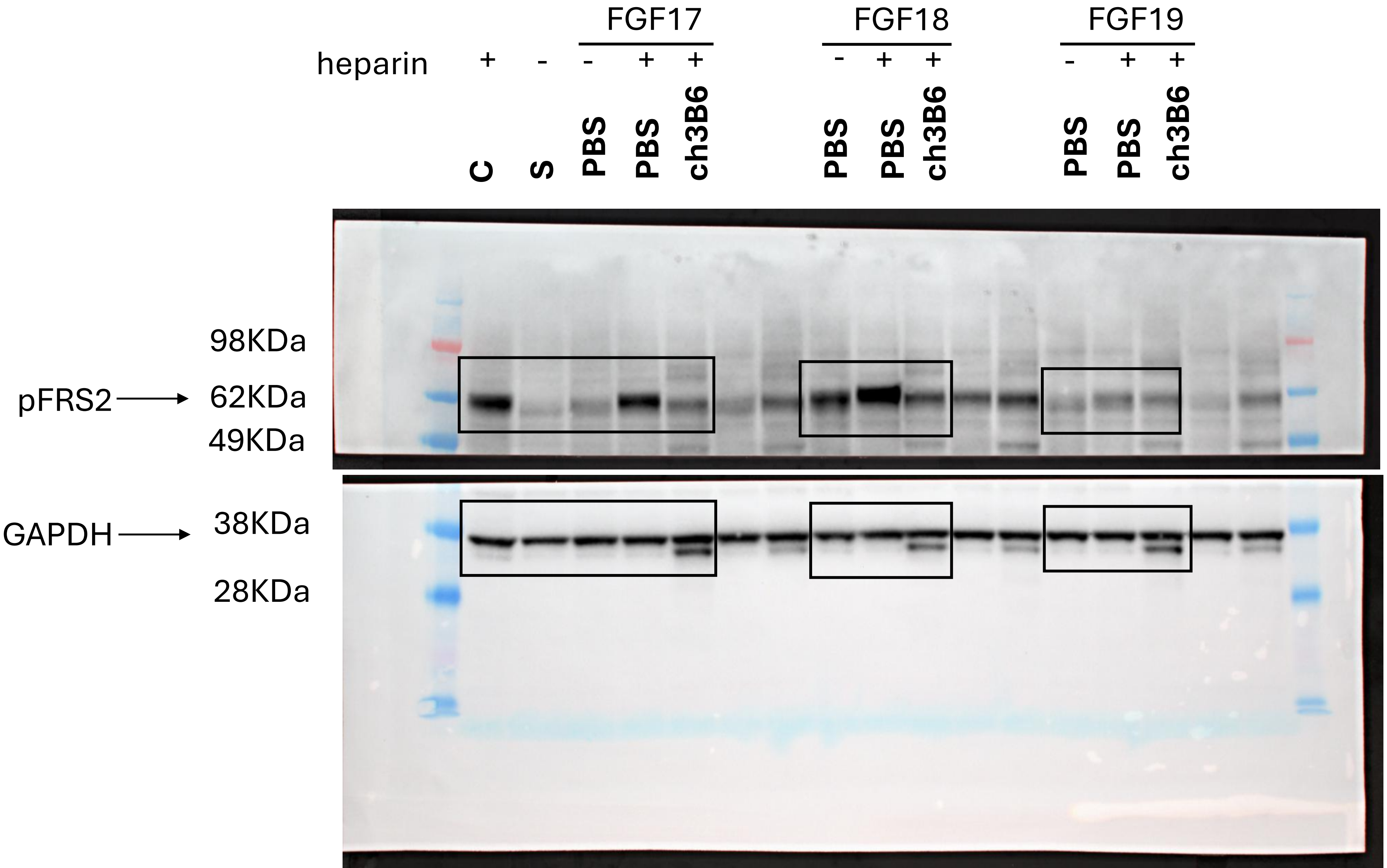

Figure 10

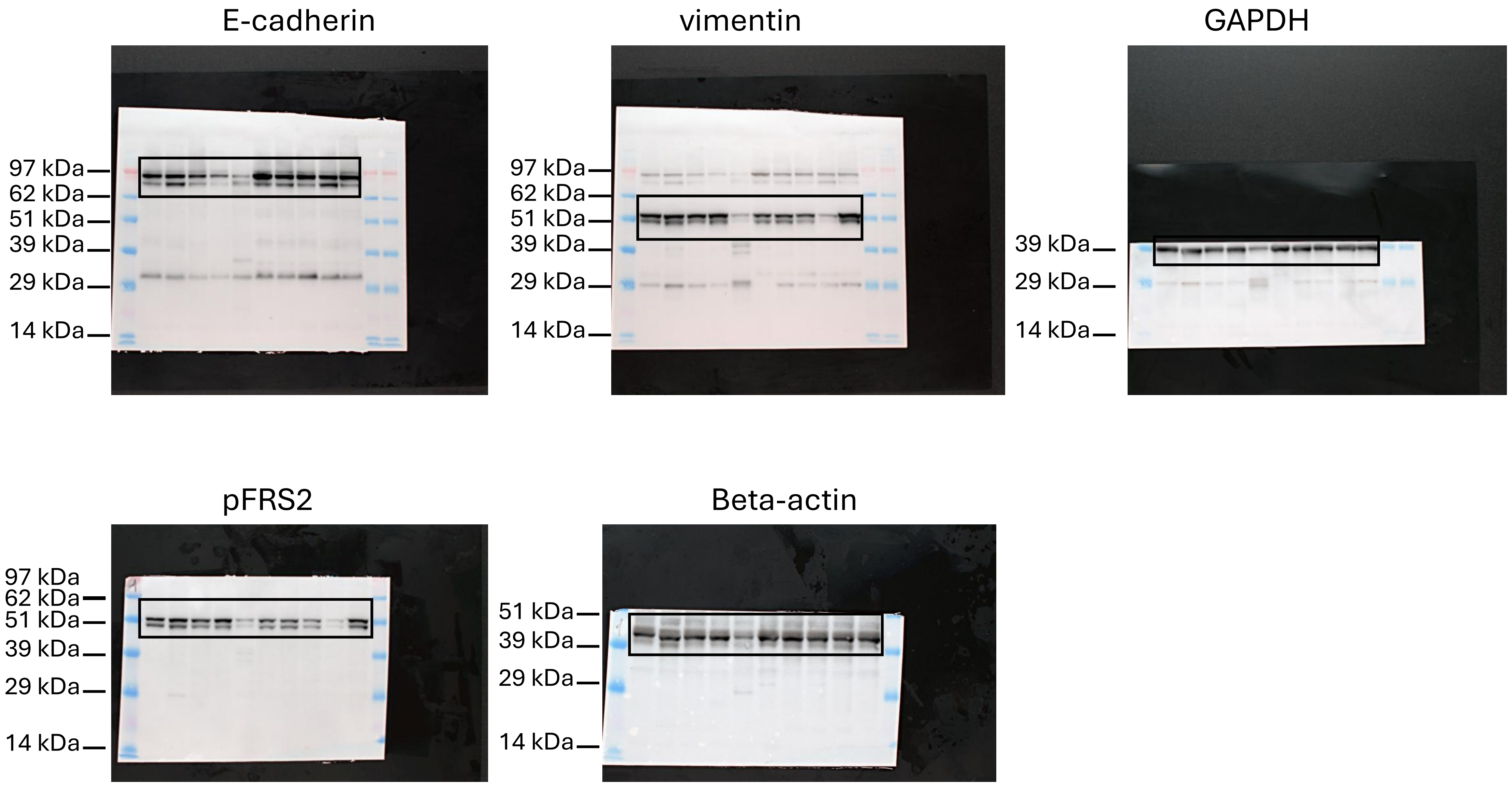
E-cadherin
97 kDa
62 kDa
51 kDa
39 kDa
29 kDa
14 kDa
vimentin
97 kDa
62 kDa
51 kDa
39 kDa
29 kDa
14 kDa
GAPDH
39 kDa
29 kDa
14 kDa
pFRS2
97 kDa
62 kDa
51 kDa
39 kDa
29 kDa
14 kDa
Beta-actin
51 kDa
39 kDa
29 kDa
14 kDa

Supplementary Figure S8

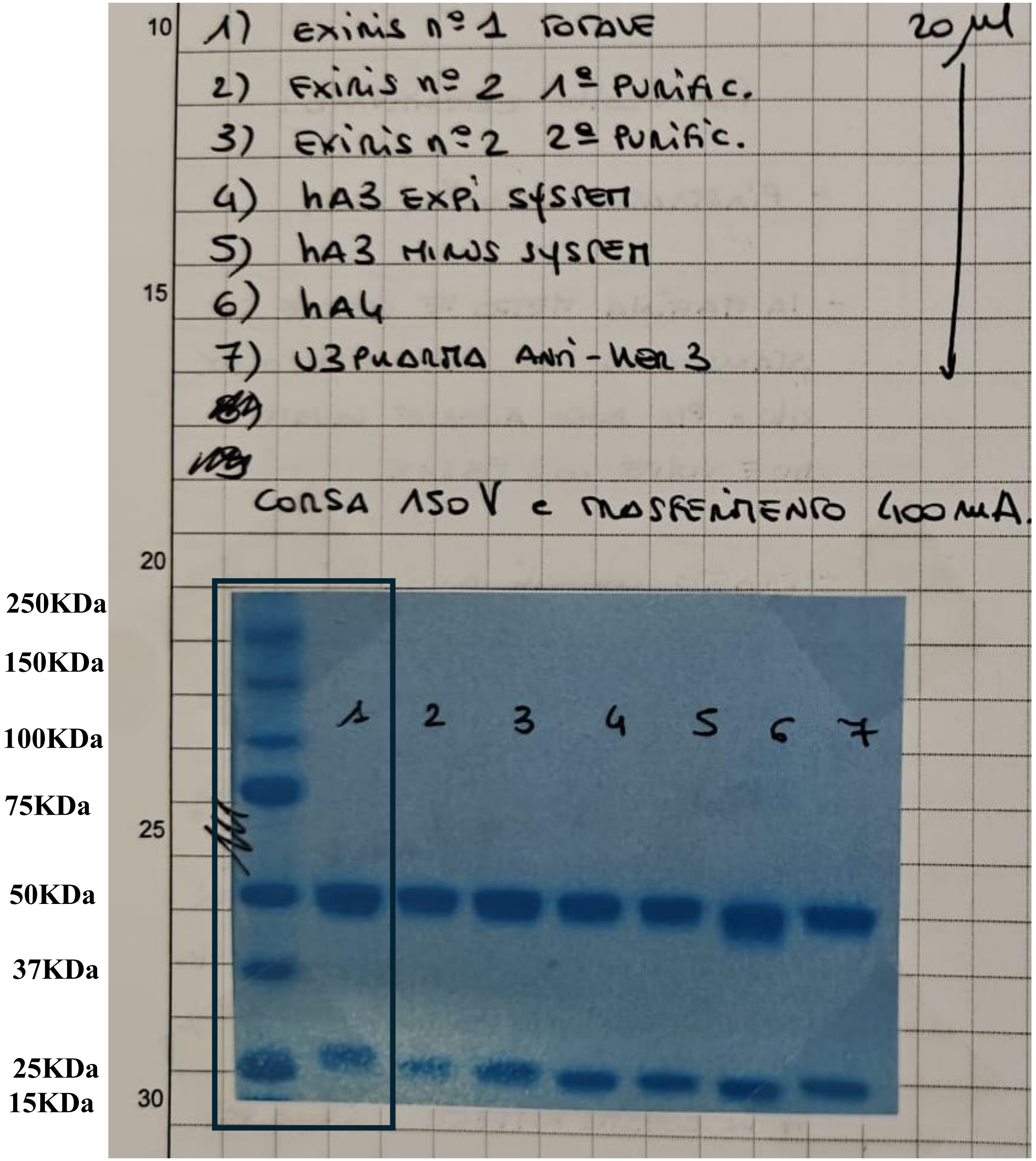

Supplement: Supplementary file 1 [file cancers-18-00418-s001.zip › File S1 uncropped gels_cancers-4061516.pdf]
