# Supplementary material for: Targeting Colorectal Cancer Stem Cells Through Inhibition of the Fibroblast Growth Factor Receptor 4 Pathway with a Novel Antibody
Source: Cancers (Basel). 2026 Jan 28;18(3):418. doi: 10.3390/cancers18030418 (PMC12896886; doi:10.3390/cancers18030418)
Supplement: Supplementary file 1 [file cancers-18-00418-s001.zip › Suppl Figures_cancer-4061516.pdf]

Supplementary Figures:

Supplementary Figure S1

GEO Accession number	Platform	Colon normal	Colon normal microdis.	Colon tumor	Colon tumor microdis.	Colon cancer cell lines	SCs	Comments
**GSE2109**	U133plus 2.0			315				
**GSE4107**	U133plus 2.0	10		12				
**GSE4183**	U133plus 2.0	8		15				
**GSE8671**	U133plus 2.0	32		32				matched pairs
**GSE9254**	U133plus 2.0	19						
**GSE9348**	U133plus 2.0	12		70				
**GSE11381**	U133plus 2.0	17						
**GSE17536**	U133plus 2.0			177				
**GSE17537**	U133plus 2.0			55				
**GSE18088**	U133plus 2.0			53				
**GSE18105**	U133plus 2.0	34			77			
**GSE20916**	U133plus 2.0	24	20	36	10			
**GSE22598**	U133plus 2.0	17		17				
**GSE23878**	U133plus 2.0	24		35				
**GSE26906**	U133plus 2.0			90				
**GSE33113**	U133plus 2.0			90				
**GSE14733**	U133plus 2.0					8		
**GSE23295**	U133plus 2.0					4		
**GSE24795**	U133plus 2.0					30		
**GSE28214**	HuGene 1.0 ST					2		HCT116 cells
**GSE34211**	U133plus 2.0					14		
**GSE35478**	U133plus 2.0					16		
**GSE35566**	U133plus 2.0					18		
**GSE36155**	U133plus 2.0					70		
**GSE17375**	U133plus 2.0						4	colon CSC
**GSE21243**	U133plus 2.0						4	Undifferentiated induced pluripotent SC
**GSE21244**	HuGene 1.0 ST						7	Various human SC
**GSE31255**	U133plus 2.0						10	Colonic SC (EPHB2-purified)
**GSE33112**	U133plus 2.0						12	colon CSC
**GSE34053**	U133plus 2.0						9	CD133 ± colon CSC

Figure S1. Datasets within the public NCBI GEO database included in the analysis

Supplementary Figure S2

A

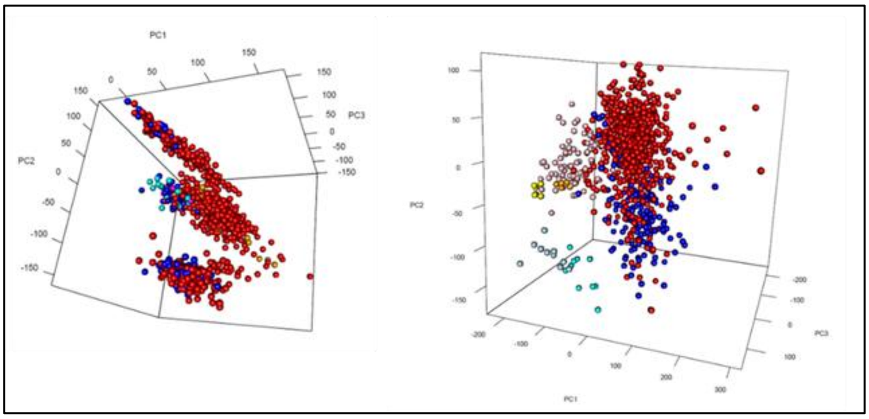

B

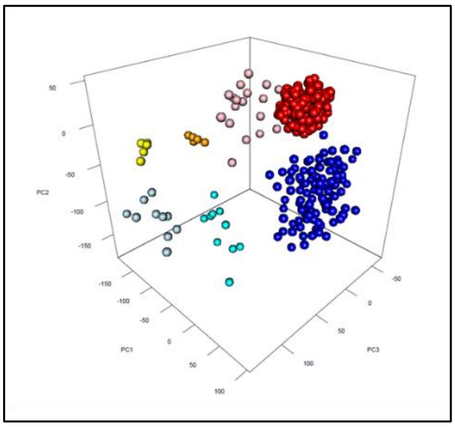

C

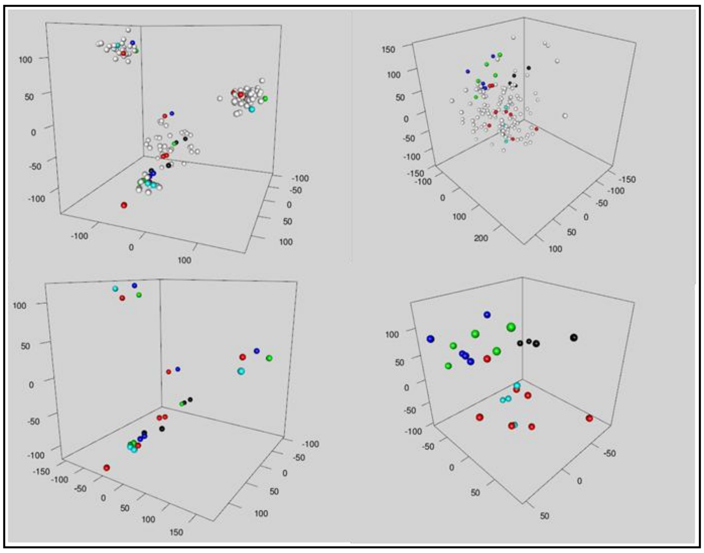

Figure S2. ***Principal component analysis (PCA) of the data in the normal and tumor colon datasets.*** *(A) PCA of normal (blue, light blue, turquoise) and tumor (red, pink, orange, yellow) colon samples, before (left) and after (right) the application of ComBat. Macrodissected normal and tumor specimens are represented in blue and red, respectively. Two different datasets of samples microdissected from normal colon are represented in light blue and turquoise, while three different datasets of samples microdissected from tumors are represented in pink, orange and yellow. (B) PCA clustering of samples from normal and tumor colon tissue after performing the selection procedure. Macrodissected normal and tumor specimens are represented in blue and red, respectively. Two different datasets of samples microdissected from normal colon are represented in light blue and turquoise, while three different datasets of samples microdissected from tumors are represented in pink, orange and yellow. (C) PCA of colon cancer cell line samples, before (left) and after (right) the application of ComBat. The top panels represent the total data set, while the bottom panels only contain cell lines represented in more than one dataset. Cell lines present in more than one dataset have the same color, while those present only once are represented in white. After correction with ComBat, the same cell lines present in multiple datasets form more compact clusters.*

Supplementary Figure S3

A

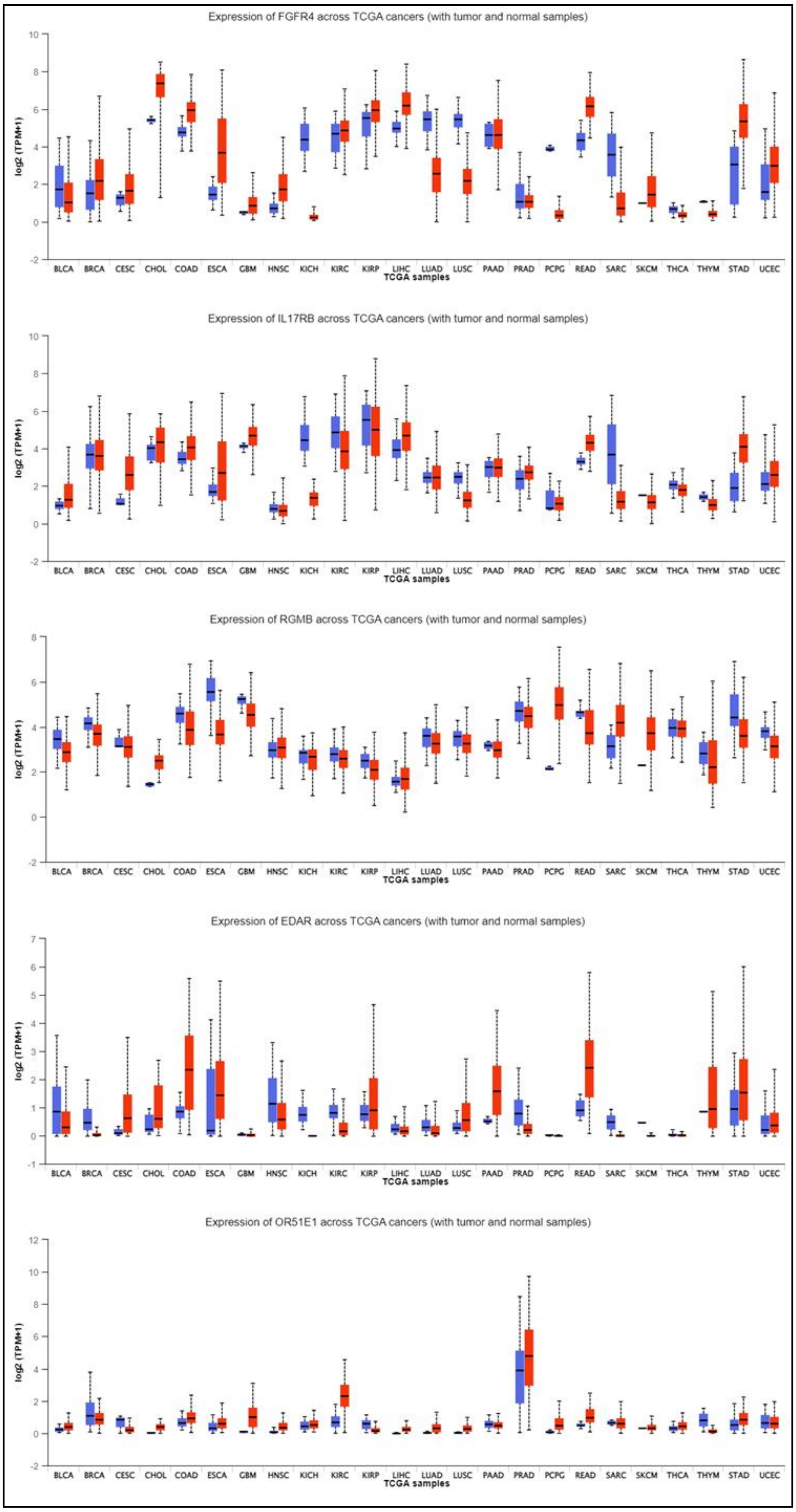

B

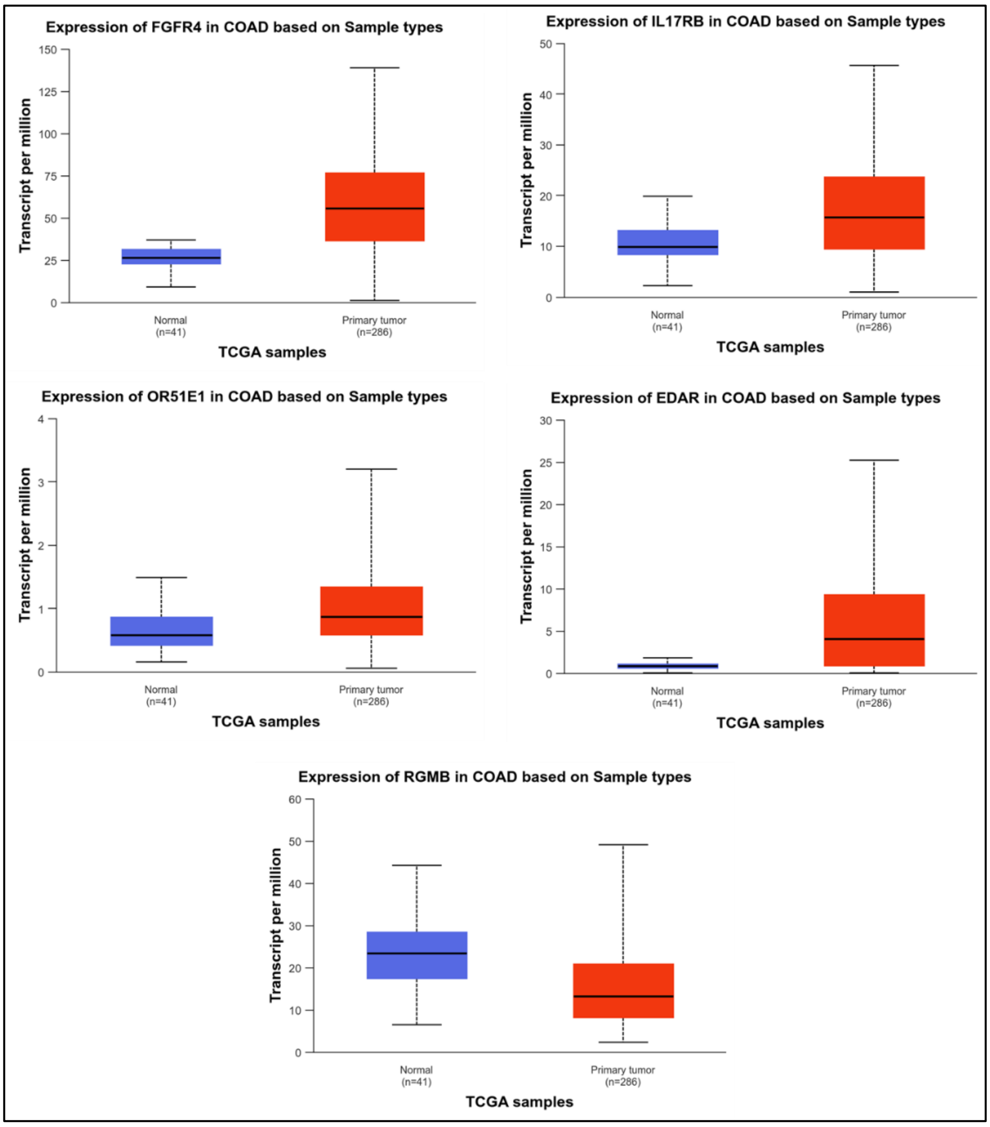

Figure S3. Expression of the five selected target genes in cancer patient samples. *(A) FGFR4, IL17RB, RGMB, EDAR, and OR51E1 expression across the TCGA pan cancer transcriptomic dataset and (B) in colon adenocarcinoma (COAD) samples. Compared with the other four selected genes, FGFR4 expression is the highest and the most tumor-specific within both the pan cancer and the COAD TCGA transcriptomic datasets.*

Supplementary Figure S4

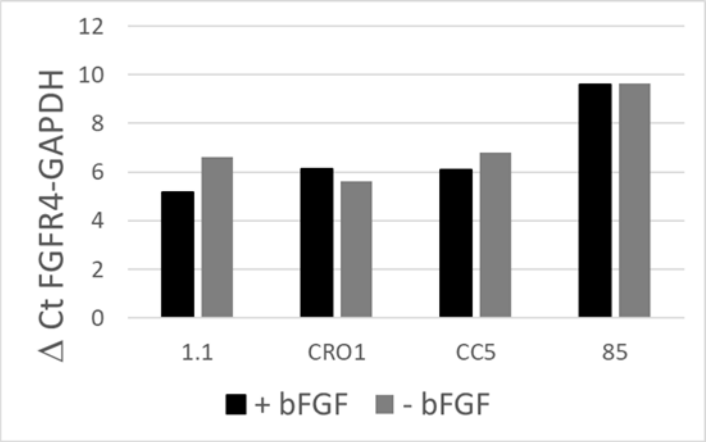

*Figure S4. **FGFR4 expression relative to GAPDH expression measured by RTqPCR in different CRC CSC lines cultured with or without bFGF in the standard CSC medium.** After around one duplication cycle, total RNA was purified, quantified and quality checked. RT-qPCR for FGFR4 and GAPDH were measured and DCt between them compared in the two culture conditions.*

Supplementary Figure S5

A

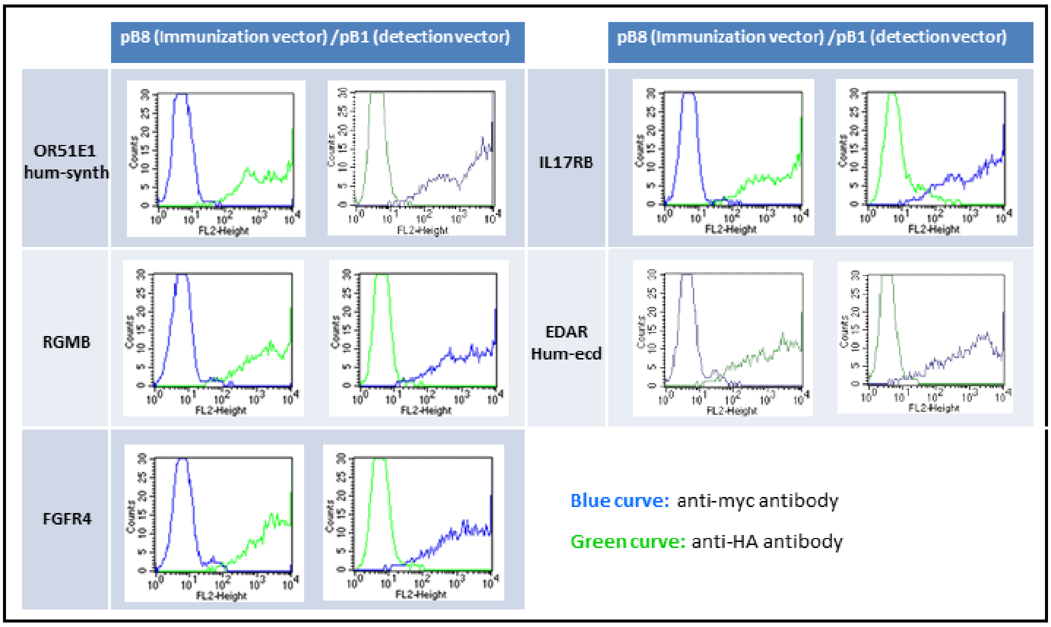

B

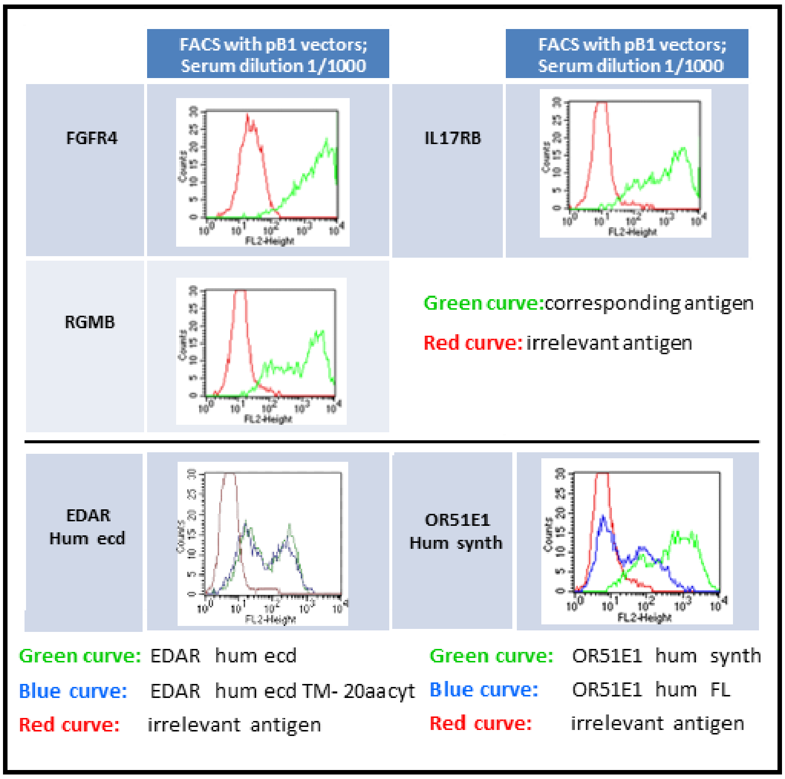

Figure S5. FACS analysis of BOSC23 overexpressing the five final targets and immunoreactivity of rat sera. *(A) Intensity of the immunogenic vector expression in BOSC23 cells by FACS analysis. Immunization vector pB8-HA and detection vector pB1-myc, shown in green and blue respectively, were cotransfected to check correct plasma membrane expression of all five antigens. (B) Specific reactivity of the immune sera of all cohorts of rats immunized with the different antigens by FACS analysis.*

Supplementary Figure S6

A

Binding parental sups to CSC cells by FACS										
	1.1		1.2		85		CRO		TOTAL RANKING	18
Parental sup	FC	RANK	FC	RANK	FC	RANK	FC	RANK		FC
1B9	14,2	**7**	5,3	**10**	7,7	**19**	12,8	**11**	**47**	1,8
1D2	2,0	**16**	3,8	**11**	12,9	**15**	2,7	**18**	**60**	1,3
1 E 7	13,7	**7**	9,5	**6**	24,4	**10**	17,1	**7**	**30**	2,3
1F9	13,6	**7**	5,0	**10**	7,6	**20**	11,9	**11**	**48**	1,9
1H10	53,6	**1**	29,2	**1**	15,3	**13**	42,1	**1**	**16**	**28,8**
2A8	7,7	**12**	7,4	**8**	15,2	**13**	11,2	**12**	**45**	1,5
2A11	2,1	**16**	2,5	**12**	2,2	**23**	3,5	**17**	**68**	1,1
2E6	4,7	**13**	2,0	**13**	8,7	**18**	7,5	**14**	**58**	1,4
2E10	3,0	**15**	4,2	**11**	14,1	**14**	11,3	**12**	**52**	2,2
2G2	10,1	**10**	6,8	**8**	11,8	**16**	15,1	**9**	**43**	1,6
2H11	1,3	**17**	1,4	**13**	1,3	**24**	1,5	**19**	**73**	1,3
3A9	4,6	**14**	2,5	**12**	2,7	**23**	6,0	**15**	**64**	1,1
3A10	1,3	**17**	1,3	**13**	1,2	**24**	1,1	**19**	**73**	1,3
3B6	11,5	**9**	7,5	**8**	13,6	**14**	16,5	**8**	**39**	2,1
3D1	3,2	**15**	2,3	**13**	1,3	**24**	3,5	**17**	**69**	2,5
3D8	17,8	**4**	7,5	**8**	9,2	**18**	20,3	**5**	**35**	2,4
3F3	1,2	**17**	3,4	**12**	3,1	**23**	5,0	**16**	**68**	1,5
3F5	2,0	**16**	3,7	**11**	11,1	**16**	3,3	**17**	**60**	1,4
3G9	1,6	**16**	3,2	**12**	2,4	**23**	4,8	**16**	**67**	1,3
4A5	1,4	**16**	2,2	**13**	2,6	**23**	3,5	**17**	**69**	1,5
4A11	9,6	**11**	12,9	**4**	9,7	**18**	16,4	**8**	**41**	**11,4**
4B1	10,3	**10**	6,8	**8**	12,7	**15**	14,8	**9**	**42**	1,9
4D7	2,7	**15**	4,8	**10**	4,1	**22**	13,8	**10**	**57**	1,2
4E10	3,2	**15**	4,5	**11**	7,3	**20**	5,7	**15**	**61**	1,3
4F8	2,3	**16**	2,5	**12**	2,1	**23**	3,3	**17**	**68**	1,1
5A5	1,8	**16**	1,7	**13**	1,6	**24**	2,3	**18**	**71**	1,2
5B5	7,2	**12**	2,1	**13**	2,7	**23**	3,8	**17**	**65**	2,0
5B6	1,6	**16**	2,1	**13**	1,6	**24**	2,5	**18**	**71**	1,2
5B9	16,9	**5**	7,2	**8**	10,4	**17**	16,7	**8**	**38**	1,9
5C9	14,5	**6**	7,1	**8**	8,2	**19**	16,3	**8**	**41**	2,0
5D3	16,9	**5**	6,9	**8**	7,5	**20**	16,2	**8**	**41**	2,2
5D6	3,5	**15**	7,5	**8**	11,8	**16**	16,0	**8**	**47**	2,2
5E1	1,3	**17**	3,7	**11**	11,1	**16**	1,8	**18**	**62**	1,3
5H6	2,1	**16**	3,8	**11**	11,9	**15**	2,5	**18**	**60**	1,3
5H9	25,3	**2**	8,2	**7**	26,2	**9**	15,5	**9**	**27**	2,1
6A11	9,8	**10**	6,0	**9**	6,5	**21**	15,8	**8**	**48**	1,7
6B5	13,2	**8**	6,6	**9**	12,3	**15**	14,7	**9**	**41**	2,0
6C9	18,0	**4**	7,6	**8**	10,8	**17**	19,0	**6**	**35**	2,4
6C11	16,9	**5**	11,4	**5**	20,9	**11**	27,6	**2**	**23**	2,9
6D11	18,7	**3**	12,2	**4**	49,2	**7**	20,6	**5**	**19**	2,1
6E2	11,1	**9**	4,3	**11**	6,7	**20**	11,1	**12**	**52**	1,8
6E7	3,4	**15**	11,3	**5**	69,3	**4**	2,2	**18**	**42**	1,5
6F4	2,5	**15**	1,8	**13**	1,9	**24**	2,6	**18**	**70**	1,5
6G5	12,6	**8**	17,1	**3**	87,5	**2**	9,6	**13**	**26**	1,5
6H3	13,0	**8**	5,4	**10**	7,7	**19**	14,4	**10**	**47**	1,7
6H5	11,8	**9**	4,4	**11**	10,4	**17**	12,8	**11**	**48**	1,8
7B5	10,2	**10**	10,6	**5**	55,2	**6**	8,8	**13**	**34**	1,7
7B7	16,3	**5**	7,1	**8**	8,8	**18**	20,1	**5**	**36**	2,2
7B11	3,9	**14**	6,7	**8**	8,8	**18**	22,9	**4**	**44**	1,6
7C1	4,6	**14**	8,3	**7**	55,6	**6**	3,9	**17**	**44**	1,6
7E4	7,4	**12**	4,5	**11**	15,6	**13**	5,7	**15**	**51**	1,6
7H1	4,3	**14**	10,2	**6**	61,5	**5**	3,2	**17**	**42**	1,4
8A4	3,4	**15**	5,2	**10**	28,0	**8**	2,1	**18**	**51**	1,3
8D4	10,9	**9**	7,2	**8**	9,7	**18**	23,9	**4**	**39**	1,8
8E3	19,1	**3**	8,4	**7**	11,3	**16**	25,3	**3**	**29**	2,3
8F10	16,1	**5**	22,4	**2**	107,2	**1**	18,9	**6**	**14**	1,9
8H5	5,6	**13**	5,6	**10**	8,2	**19**	17,2	**7**	**49**	2,0
8H9	2,3	**16**	1,5	**13**	1,7	**24**	2,0	**18**	**71**	1,3
8H10	15,4	**6**	6,2	**9**	10,9	**16**	16,7	**8**	**39**	1,9
9B1	13,0	**8**	5,6	**10**	8,7	**19**	16,6	**8**	**45**	1,8
9C5	8,1	**12**	3,8	**11**	13,7	**14**	7,0	**14**	**51**	1,3
9C6	10,5	**10**	10,3	**6**	84,5	**3**	4,5	**16**	**35**	1,8
9F7	9,4	**11**	5,5	**10**	19,2	**12**	7,6	**14**	**47**	1,4
9H4	4,1	**14**	3,1	**12**	3,7	**22**	7,1	**14**	**62**	1,4
9H5	3,6	**14**	2,6	**12**	2,6	**23**	6,3	**15**	**64**	1,4
10A11	2,2	**16**	1,6	**13**	1,6	**24**	2,4	**18**	**71**	1,4
10D4	8,1	**12**	3,3	**12**	4,3	**22**	6,4	**15**	**61**	1,5
10D9	6,2	**13**	4,9	**10**	20,4	**11**	4,9	**16**	**50**	1,4
10G2	10,7	**10**	4,2	**11**	5,6	**21**	11,7	**12**	**54**	1,4
10G5	13,6	**7**	5,7	**9**	9,5	**18**	17,9	**7**	**41**	2,1
10G11	13,1	**8**	8,9	**7**	49,2	**7**	14,4	**10**	**32**	1,7
Anti-FGFR4	18,0		8,0		10,0		22,0			3,0

B

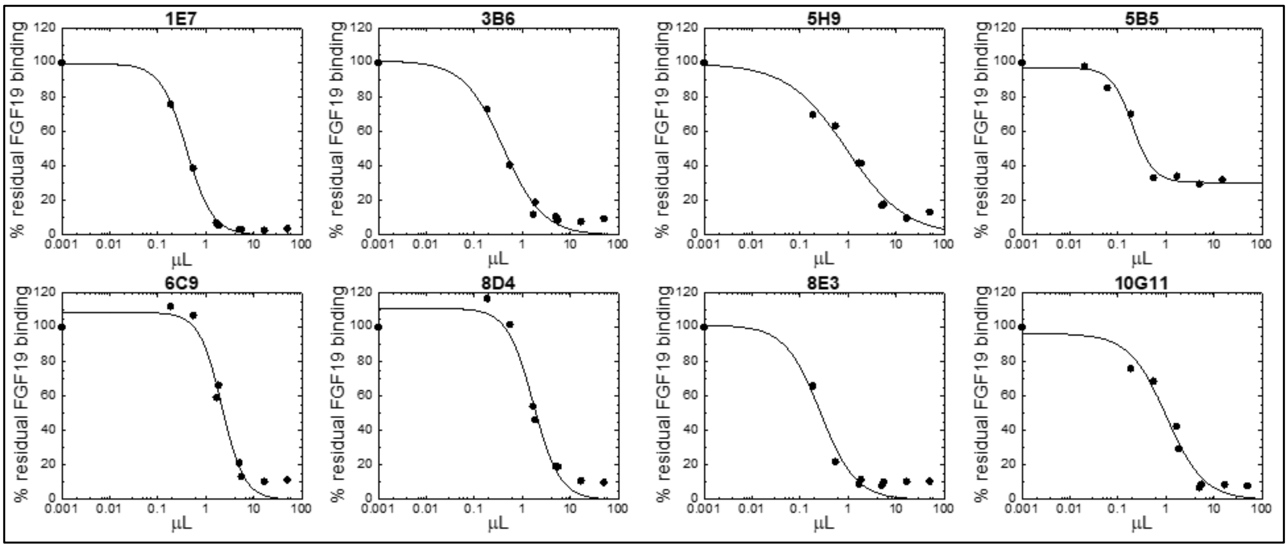

Figure S6. Summary of FACS data and FGF19 ELISA competion assays obtained with the 70 supernatants on 5 different CSC lines. *(**A**) FACS analysis on different cells. Fold change (FC) is the ratio of the mean fluorescence intensity (MFI) measured with each supernatant to the negative control (myeloma cells conditioned medium). A score was assigned based on FC values on each FGFR4-positive CSC line and the final rank was calculated by adding the individual scores (total ranking). Supernatants that did not show cross-reactivity with the murine receptor are blue-coloured and underlined. (**B**) Inhibition of FGF19 binding to FGFR4 in a solid-phase assay by antibody-containing supernatants.*

Supplementary Figure S7

Variable region comparison of heavy chains from 8 anti-FGFR4 monoclonal antibodies

	CDR1	CDR2	CDR3	ISOTYPE
BMK-1E7-C4	GFTFSNY	NPSGTR	LGEGGFFDY	IgG2b
BMK-3B6-E4	GFTFSNY	NPSGTR	LYNNYAFDY	IgG2b
BFG-5B5-G7	GFSLTSY	WAGGH	GWFTGIFDY	IgG1/IgG2a
BMK-5H9-D1	GYTFTDY	NTYTGK	YLWVPGFNVYYAMDA	IgG2a
BMK-6C9-C11	GFSLTSY	WAGGH	GWFTGIFDY	IgG1/IgG2a
BMK-8D4-E2	GFTFSTF	STSGDR	HLGYGGMDA	IgG2b
BMK-8E3-E4	GFSLIKY	WNDGD	GSDYGYNSYWYFDF	IgG2a
BMK-10G11-F3	GYNIRNS	DPASGN	TVVIRGSDAMDA	IgG2b

Variable region comparison of light chains from 8 anti-FGFR4 monoclonal antibodies

	CDR1	CDR2	CDR3	ISOTYPE
BMK-1E7-C4	RASESVGTLMH	GASNLES	QQSWNDPPT	kappa
BMK-3B6-E4	RASESVSTLMH	GTSNLES	QQSWNDPPT	kappa
BFG-5B5-G7	LPSEDIFTDLA	NANSLQN	QQYNNYPLT	kappa
BMK-5H9-D1	KASQNIDKFLD	NTHSLHT	LQHNSGYT	kappa
BMK-6C9-C11	LPSEDIFSDLA	NANSLQN	QQYNNYPLT	kappa
BMK-8D4-E2	LASEDIYDILA	DASSLHP	QNGFSAPFT	kappa
BMK-8E3-E4	RASEDIYSNLA	DANRLAD	QQYNDYPNT	kappa
BMK-10G11-F3	LASEDIYNDLA	FASSLQD	LQDSEYPLT	kappa

Figure S7. Characterization of the eight selected anti-FGFR4 rat antibodies. *Amino acid sequences of the VH and VL CDRs and isotypes. 1E7-C4 and 3B6-E4 (blue) have identical VH CDR1 and CDR2, differing instead in the VH CDR3. These two antibodies are equally very similar in the VL CDRs, with a single amino acid difference in both the CDR1 and CDR2 regions. Likewise, the sequences of the CDRs of 5B5-G7 were almost identical to those of 6C9-C11 (purple), with a single amino acid difference in the VL CDR1. The other five antibodies instead present significant differences in all CDRs.*

Supplementary Figure S8

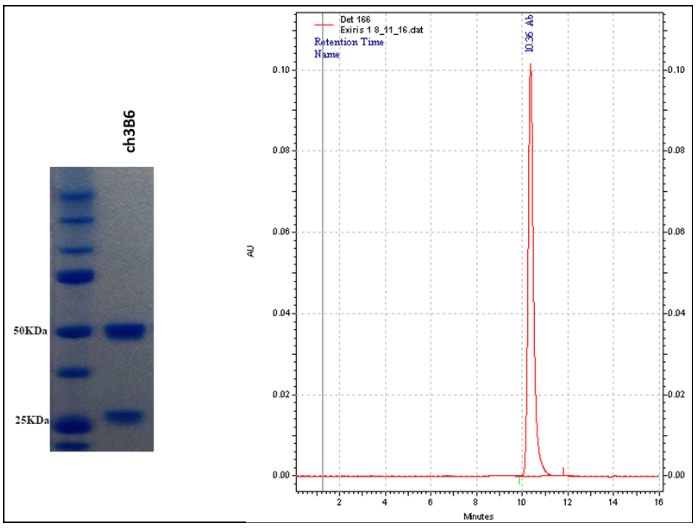

Figure S8. Quality controls of the antibody ch3B6 purification*. Left: SDS-PAGE under reducing conditions; right: HPLC-SEC. Similar results were obtained for the antibodies ch5B5 and ch6C9.*

Supplementary Figure S9

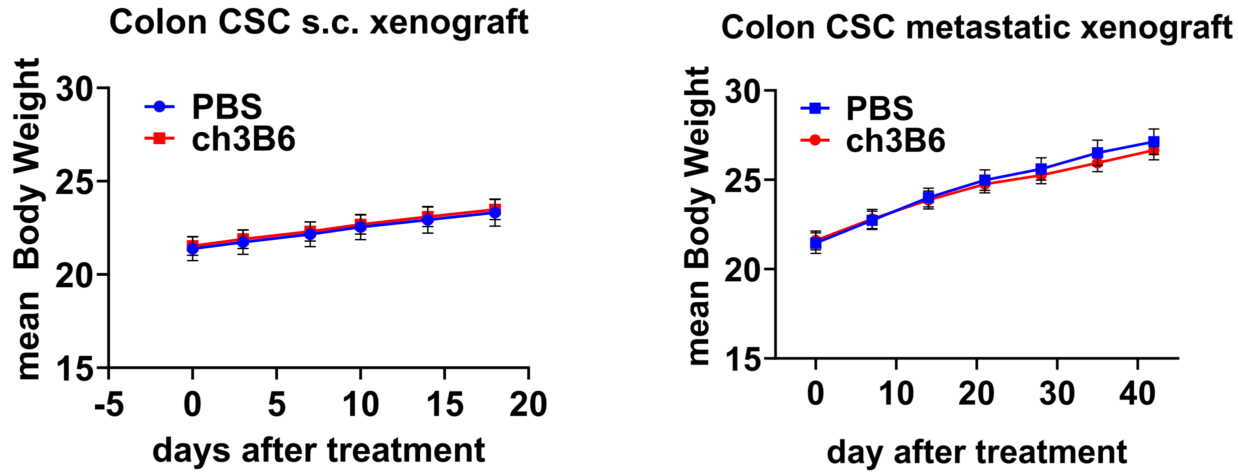

Figure S9. Body weight changes during in vivo efficacy studies. *Body weight was measured during time after treatments with ch3B6 antibody in the subcutaneous (s.c.) xenograft, on the left hand side of the Figure, and in the liver metastatic model, on the right hand side of the Figure.*
