# Supplementary material for: Targeting Colorectal Cancer Stem Cells Through Inhibition of the Fibroblast Growth Factor Receptor 4 Pathway with a Novel Antibody
Source: Cancers (Basel). 2026 Jan 28;18(3):418. doi: 10.3390/cancers18030418 (PMC12896886; doi:10.3390/cancers18030418)
Supplement: Supplementary file 1 [file cancers-18-00418-s001.zip › Table S1 Bionformatic ID of CSC-enriched genes_cancers-4061516.pdf]

Table S1: Bionformatic ID of CSC-enriched genes

ComBat Datasets List

	Sample	Dataset	ComBat batch	ComBat Covariant1
1	ctsc11_stem	This manuscript	Human Gene 1.0 ST	CSC
2	ctsc12_stem	This manuscript	Human Gene 1.0 ST	CSC
3	ctsc18_stem	This manuscript	Human Gene 1.0 ST	CSC
4	ctsc85_stem	This manuscript	Human Gene 1.0 ST	CSC
5	ctscCro1_stem	This manuscript	Human Gene 1.0 ST	CSC
6	CC1S	This manuscript	Human Gene 1.0 ST	CSC
7	CC2S	This manuscript	Human Gene 1.0 ST	CSC
8	CC5S	This manuscript	Human Gene 1.0 ST	CSC
9	ctsc11_diff	This manuscript	Human Gene 1.0 ST	CSC
10	ctsc12_diff	This manuscript	Human Gene 1.0 ST	CSC
11	ctsc18_diff	This manuscript	Human Gene 1.0 ST	CSC
12	ctsc85_diff	This manuscript	Human Gene 1.0 ST	CSC
13	ctscCro1_diff	This manuscript	Human Gene 1.0 ST	CSC
14	CC1D	This manuscript	Human Gene 1.0 ST	CSC
15	CC2SD	This manuscript	Human Gene 1.0 ST	CSC
16	CC5SD	This manuscript	Human Gene 1.0 ST	CSC
17	GSM93939.CEL	GSE4107	U133_plus2	normal
18	GSM93952.CEL	GSE4107	U133_plus2	normal
19	GSM95473.CEL	GSE4107	U133_plus2	normal
20	GSM95475.CEL	GSE4107	U133_plus2	normal
21	GSM95476.CEL	GSE4107	U133_plus2	normal
22	GSM95480.CEL	GSE4107	U133_plus2	normal
23	GSM215052.CEL	GSE8671	U133_plus2	normal
24	GSM215053.CEL	GSE8671	U133_plus2	normal
25	GSM215054.CEL	GSE8671	U133_plus2	normal
26	GSM215055.CEL	GSE8671	U133_plus2	normal
27	GSM215057.CEL	GSE8671	U133_plus2	normal
28	GSM215058.CEL	GSE8671	U133_plus2	normal
29	GSM215059.CEL	GSE8671	U133_plus2	normal
30	GSM215060.CEL	GSE8671	U133_plus2	normal
31	GSM215064.CEL	GSE8671	U133_plus2	normal
32	GSM215066.CEL	GSE8671	U133_plus2	normal
33	GSM215068.CEL	GSE8671	U133_plus2	normal
34	GSM215070.CEL	GSE8671	U133_plus2	normal
35	GSM215071.CEL	GSE8671	U133_plus2	normal
36	GSM215072.CEL	GSE8671	U133_plus2	normal
37	GSM215073.CEL	GSE8671	U133_plus2	normal
38	GSM215074.CEL	GSE8671	U133_plus2	normal
39	GSM215075.CEL	GSE8671	U133_plus2	normal
40	GSM215076.CEL	GSE8671	U133_plus2	normal
41	GSM215077.CEL	GSE8671	U133_plus2	normal
42	GSM215078.CEL	GSE8671	U133_plus2	normal
43	GSM215079.CEL	GSE8671	U133_plus2	normal
44	GSM215080.CEL	GSE8671	U133_plus2	normal
45	GSM215081.CEL	GSE8671	U133_plus2	normal
46	GSM215082.CEL	GSE8671	U133_plus2	normal
47	GSM234912.CEL	GSE9254	U133_plus2	normal
48	GSM234914.CEL	GSE9254	U133_plus2	normal
49	GSM234915.CEL	GSE9254	U133_plus2	normal
50	GSM234916.CEL	GSE9254	U133_plus2	normal
51	GSM234918.CEL	GSE9254	U133_plus2	normal
52	GSM234920.CEL	GSE9254	U133_plus2	normal
53	GSM234922.CEL	GSE9254	U133_plus2	normal
54	GSM234923.CEL	GSE9254	U133_plus2	normal
55	GSM234925.CEL	GSE9254	U133_plus2	normal
56	GSM234927.CEL	GSE9254	U133_plus2	normal
57	GSM237984.CEL	GSE9254	U133_plus2	normal
58	GSM237985.CEL	GSE9254	U133_plus2	normal
59	GSM237986.CEL	GSE9254	U133_plus2	normal
60	GSM237988.CEL	GSE9254	U133_plus2	normal
61	GSM237993.CEL	GSE9254	U133_plus2	normal
62	GSM237994.CEL	GSE9254	U133_plus2	normal
63	GSM237995.CEL	GSE9254	U133_plus2	normal
64	GSM298776_C31KN3.CEL	GSE11831	U133_plus2	normal
65	GSM298778_C36KN3.CEL	GSE11831	U133_plus2	normal
66	GSM298782_C37KN1.CEL	GSE11831	U133_plus2	normal

67	GSM298789_C45KN5.CEL	GSE11831	U133_plus2	normal
68	GSM298950_C73KN2.CEL	GSE11831	U133_plus2	normal
69	GSM298951_C79KN3.CEL	GSE11831	U133_plus2	normal
70	GSM298952_C80KN2.CEL	GSE11831	U133_plus2	normal
71	GSM298953_C82KN2.CEL	GSE11831	U133_plus2	normal
72	GSM298956_C86KN2.CEL	GSE11831	U133_plus2	normal
73	GSM298958_C87KN1.CEL	GSE11831	U133_plus2	normal
74	GSM298962_C92KN2.CEL	GSE11831	U133_plus2	normal
75	GSM298964_C97KN1.CEL	GSE11831	U133_plus2	normal
76	GSM452634.CEL	GSE18105	U133_plus2	normal
77	GSM452635.CEL	GSE18105	U133_plus2	normal
78	GSM452638.CEL	GSE18105	U133_plus2	normal
79	GSM452640.CEL	GSE18105	U133_plus2	normal
80	GSM452642.CEL	GSE18105	U133_plus2	normal
81	GSM452643.CEL	GSE18105	U133_plus2	normal
82	GSM452644.CEL	GSE18105	U133_plus2	normal
83	GSM452645.CEL	GSE18105	U133_plus2	normal
84	GSM523290_ColonCa_Seria02_1K.CEL	GSE20916	U133_plus2	normal
85	GSM523291_ColonCa_Seria02_27A.CEL	GSE20916	U133_plus2	normal
86	GSM523304_ColonCa_Seria04_33A.CEL	GSE20916	U133_plus2	normal
87	GSM523311_ColonCa_Seria05_41A.CEL	GSE20916	U133_plus2	normal
88	GSM523314_ColonCa_Seria05_5K.CEL	GSE20916	U133_plus2	normal
89	GSM523330_ColonCa_Seria08_31A_2.CEL	GSE20916	U133_plus2	normal
90	GSM523338_ColonCa_Seria09_9A.CEL	GSE20916	U133_plus2	normal
91	GSM523349_ColonCa_Seria11_16A.CEL	GSE20916	U133_plus2	normal
92	GSM523360_ColonCa_Seria12_9K.CEL	GSE20916	U133_plus2	normal
93	GSM523364_ColonCa_Seria13_12A.CEL	GSE20916	U133_plus2	normal
94	GSM523365_ColonCa_Seria13_25B1N.CEL	GSE20916	U133_plus2	normal
95	GSM523369_ColonCa_Seria13_4K.CEL	GSE20916	U133_plus2	normal
96	GSM523377_ColonCa_Seria14_48A.CEL	GSE20916	U133_plus2	normal
97	GSM523382_ColonCa_Seria15_8A.CEL	GSE20916	U133_plus2	normal
98	GSM452630.CEL.gz	GSE22598	U133_plus2	normal
99	GSM452632.CEL.gz	GSE22598	U133_plus2	normal
100	GSM452633.CEL.gz	GSE22598	U133_plus2	normal
101	GSM452634.CEL.gz	GSE22598	U133_plus2	normal
102	GSM452635.CEL.gz	GSE22598	U133_plus2	normal
103	GSM452638.CEL.gz	GSE22598	U133_plus2	normal
104	GSM452640.CEL.gz	GSE22598	U133_plus2	normal
105	GSM452642.CEL.gz	GSE22598	U133_plus2	normal
106	GSM452643.CEL.gz	GSE22598	U133_plus2	normal
107	GSM452644.CEL.gz	GSE22598	U133_plus2	normal
108	GSM452645.CEL.gz	GSE22598	U133_plus2	normal
109	GSM588863.CEL.gz	GSE22598	U133_plus2	normal
110	GSM588864.CEL.gz	GSE22598	U133_plus2	normal
111	GSM588865.CEL.gz	GSE22598	U133_plus2	normal
112	GSM588866.CEL.gz	GSE22598	U133_plus2	normal
113	GSM588869.CEL.gz	GSE22598	U133_plus2	normal
114	GSM588873.CEL.gz	GSE22598	U133_plus2	normal
115	GSM588876.CEL.gz	GSE22598	U133_plus2	normal
116	GSM588877.CEL.gz	GSE22598	U133_plus2	normal
117	GSM588878.CEL.gz	GSE22598	U133_plus2	normal
118	GSM588882.CEL.gz	GSE22598	U133_plus2	normal
119	GSM588884.CEL.gz	GSE22598	U133_plus2	normal
120	GSM588885.CEL.gz	GSE22598	U133_plus2	normal
121	GSM588886.CEL.gz	GSE22598	U133_plus2	normal
122	GSM38077	GSE2109	U133_plus2	tumor
123	GSM38105	GSE2109	U133_plus2	tumor
124	GSM46823	GSE2109	U133_plus2	tumor
125	GSM46901	GSE2109	U133_plus2	tumor
126	GSM46930	GSE2109	U133_plus2	tumor
127	GSM46956	GSE2109	U133_plus2	tumor
128	GSM53070	GSE2109	U133_plus2	tumor
129	GSM53073	GSE2109	U133_plus2	tumor
130	GSM53083	GSE2109	U133_plus2	tumor
131	GSM53106	GSE2109	U133_plus2	tumor
132	GSM53142	GSE2109	U133_plus2	tumor
133	GSM76490	GSE2109	U133_plus2	tumor
134	GSM76524	GSE2109	U133_plus2	tumor
135	GSM76530	GSE2109	U133_plus2	tumor
136	GSM76555	GSE2109	U133_plus2	tumor
137	GSM76576	GSE2109	U133_plus2	tumor

138	GSM76583	GSE2109	U133_plus2	tumor
139	GSM76608	GSE2109	U133_plus2	tumor
140	GSM76617	GSE2109	U133_plus2	tumor
141	GSM88963	GSE2109	U133_plus2	tumor
142	GSM88968	GSE2109	U133_plus2	tumor
143	GSM88994	GSE2109	U133_plus2	tumor
144	GSM89040	GSE2109	U133_plus2	tumor
145	GSM89044	GSE2109	U133_plus2	tumor
146	GSM89047	GSE2109	U133_plus2	tumor
147	GSM89049	GSE2109	U133_plus2	tumor
148	GSM89053	GSE2109	U133_plus2	tumor
149	GSM89061	GSE2109	U133_plus2	tumor
150	GSM89062	GSE2109	U133_plus2	tumor
151	GSM89090	GSE2109	U133_plus2	tumor
152	GSM89103	GSE2109	U133_plus2	tumor
153	GSM102429	GSE2109	U133_plus2	tumor
154	GSM102431	GSE2109	U133_plus2	tumor
155	GSM102442	GSE2109	U133_plus2	tumor
156	GSM102460	GSE2109	U133_plus2	tumor
157	GSM102472	GSE2109	U133_plus2	tumor
158	GSM102497	GSE2109	U133_plus2	tumor
159	GSM102501	GSE2109	U133_plus2	tumor
160	GSM102516	GSE2109	U133_plus2	tumor
161	GSM102519	GSE2109	U133_plus2	tumor
162	GSM102533	GSE2109	U133_plus2	tumor
163	GSM102540	GSE2109	U133_plus2	tumor
164	GSM102549	GSE2109	U133_plus2	tumor
165	GSM102551	GSE2109	U133_plus2	tumor
166	GSM102579	GSE2109	U133_plus2	tumor
167	GSM117642	GSE2109	U133_plus2	tumor
168	GSM117662	GSE2109	U133_plus2	tumor
169	GSM117673	GSE2109	U133_plus2	tumor
170	GSM117678	GSE2109	U133_plus2	tumor
171	GSM117681	GSE2109	U133_plus2	tumor
172	GSM117709	GSE2109	U133_plus2	tumor
173	GSM117746	GSE2109	U133_plus2	tumor
174	GSM117760	GSE2109	U133_plus2	tumor
175	GSM117775	GSE2109	U133_plus2	tumor
176	GSM137929	GSE2109	U133_plus2	tumor
177	GSM137947	GSE2109	U133_plus2	tumor
178	GSM137964	GSE2109	U133_plus2	tumor
179	GSM137967	GSE2109	U133_plus2	tumor
180	GSM138018	GSE2109	U133_plus2	tumor
181	GSM138042	GSE2109	U133_plus2	tumor
182	GSM138044	GSE2109	U133_plus2	tumor
183	GSM138048	GSE2109	U133_plus2	tumor
184	GSM152573	GSE2109	U133_plus2	tumor
185	GSM152579	GSE2109	U133_plus2	tumor
186	GSM152582	GSE2109	U133_plus2	tumor
187	GSM152692	GSE2109	U133_plus2	tumor
188	GSM152720	GSE2109	U133_plus2	tumor
189	GSM152790	GSE2109	U133_plus2	tumor
190	GSM152799	GSE2109	U133_plus2	tumor
191	GSM179795	GSE2109	U133_plus2	tumor
192	GSM179803	GSE2109	U133_plus2	tumor
193	GSM179867	GSE2109	U133_plus2	tumor
194	GSM179882	GSE2109	U133_plus2	tumor
195	GSM179887	GSE2109	U133_plus2	tumor
196	GSM179888	GSE2109	U133_plus2	tumor
197	GSM179908	GSE2109	U133_plus2	tumor
198	GSM179925	GSE2109	U133_plus2	tumor
199	GSM179930	GSE2109	U133_plus2	tumor
200	GSM203653	GSE2109	U133_plus2	tumor
201	GSM203691	GSE2109	U133_plus2	tumor
202	GSM203743	GSE2109	U133_plus2	tumor
203	GSM231928	GSE2109	U133_plus2	tumor
204	GSM231958	GSE2109	U133_plus2	tumor
205	GSM231959	GSE2109	U133_plus2	tumor
206	GSM231961	GSE2109	U133_plus2	tumor
207	GSM231964	GSE2109	U133_plus2	tumor
208	GSM277690	GSE2109	U133_plus2	tumor

209	GSM325816	GSE2109	U133_plus2	tumor
210	GSM325818	GSE2109	U133_plus2	tumor
211	GSM353934	GSE2109	U133_plus2	tumor
212	GSM215084.CEL	GSE8671	U133_plus2	tumor
213	GSM215089.CEL	GSE8671	U133_plus2	tumor
214	GSM215091.CEL	GSE8671	U133_plus2	tumor
215	GSM215097.CEL	GSE8671	U133_plus2	tumor
216	GSM215098.CEL	GSE8671	U133_plus2	tumor
217	GSM215101.CEL	GSE8671	U133_plus2	tumor
218	GSM215104.CEL	GSE8671	U133_plus2	tumor
219	GSM215114.CEL	GSE8671	U133_plus2	tumor
220	GSM237931.CEL	GSE8671	U133_plus2	tumor
221	GSM237932.CEL	GSE8671	U133_plus2	tumor
222	GSM237940.CEL	GSE8671	U133_plus2	tumor
223	GSM237948.CEL	GSE8671	U133_plus2	tumor
224	GSM237953.CEL	GSE8671	U133_plus2	tumor
225	GSM237960.CEL	GSE8671	U133_plus2	tumor
226	GSM237962.CEL	GSE8671	U133_plus2	tumor
227	GSM437093.CEL.gz	GSE17536	U133_plus2	tumor
228	GSM437096.CEL.gz	GSE17536	U133_plus2	tumor
229	GSM437098.CEL.gz	GSE17536	U133_plus2	tumor
230	GSM437103.CEL.gz	GSE17536	U133_plus2	tumor
231	GSM437116.CEL.gz	GSE17536	U133_plus2	tumor
232	GSM437118.CEL.gz	GSE17536	U133_plus2	tumor
233	GSM437121.CEL.gz	GSE17536	U133_plus2	tumor
234	GSM437124.CEL.gz	GSE17536	U133_plus2	tumor
235	GSM437126.CEL.gz	GSE17536	U133_plus2	tumor
236	GSM437138.CEL.gz	GSE17536	U133_plus2	tumor
237	GSM437144.CEL.gz	GSE17536	U133_plus2	tumor
238	GSM437164.CEL.gz	GSE17536	U133_plus2	tumor
239	GSM437170.CEL.gz	GSE17536	U133_plus2	tumor
240	GSM437188.CEL.gz	GSE17536	U133_plus2	tumor
241	GSM437189.CEL.gz	GSE17536	U133_plus2	tumor
242	GSM437192.CEL.gz	GSE17536	U133_plus2	tumor
243	GSM437195.CEL.gz	GSE17536	U133_plus2	tumor
244	GSM437212.CEL.gz	GSE17536	U133_plus2	tumor
245	GSM437218.CEL.gz	GSE17536	U133_plus2	tumor
246	GSM437219.CEL.gz	GSE17536	U133_plus2	tumor
247	GSM437220.CEL.gz	GSE17536	U133_plus2	tumor
248	GSM437229.CEL.gz	GSE17536	U133_plus2	tumor
249	GSM437238.CEL.gz	GSE17536	U133_plus2	tumor
250	GSM437243.CEL.gz	GSE17536	U133_plus2	tumor
251	GSM437245.CEL.gz	GSE17536	U133_plus2	tumor
252	GSM437258.CEL.gz	GSE17536	U133_plus2	tumor
253	GSM437260.CEL.gz	GSE17536	U133_plus2	tumor
254	GSM437261.CEL.gz	GSE17536	U133_plus2	tumor
255	GSM437262.CEL.gz	GSE17536	U133_plus2	tumor
256	GSM437267.CEL.gz	GSE17536	U133_plus2	tumor
257	GSM437279.CEL.gz	GSE17536	U133_plus2	tumor
258	GSM437280.CEL.gz	GSE17536	U133_plus2	tumor
259	GSM437293.CEL.gz	GSE17536	U133_plus2	tumor
260	GSM437308.CEL.gz	GSE17536	U133_plus2	tumor
261	GSM437323.CEL.gz	GSE17536	U133_plus2	tumor
262	GSM452149.CEL.gz	GSE18088	U133_plus2	tumor
263	GSM452150.CEL.gz	GSE18088	U133_plus2	tumor
264	GSM452151.CEL.gz	GSE18088	U133_plus2	tumor
265	GSM452160.CEL.gz	GSE18088	U133_plus2	tumor
266	GSM452168.CEL.gz	GSE18088	U133_plus2	tumor
267	GSM452173.CEL.gz	GSE18088	U133_plus2	tumor
268	GSM452175.CEL.gz	GSE18088	U133_plus2	tumor
269	GSM452184.CEL.gz	GSE18088	U133_plus2	tumor
270	GSM452185.CEL.gz	GSE18088	U133_plus2	tumor
271	GSM452187.CEL.gz	GSE18088	U133_plus2	tumor
272	GSM452188.CEL.gz	GSE18088	U133_plus2	tumor
273	GSM452193.CEL.gz	GSE18088	U133_plus2	tumor
274	GSM452194.CEL.gz	GSE18088	U133_plus2	tumor
275	GSM523345_ColonCa_Seria10_433B1.CEL	GSE20916	U133_plus2	tumor
276	GSM523352_ColonCa_Seria11_31IIB1.CEL	GSE20916	U133_plus2	tumor
277	GSM523379_ColonCa_Seria15_2B2D.CEL	GSE20916	U133_plus2	tumor
278	GSM452652.CEL.gz	GSE20916	U133_plus2	tumor
279	GSM452657.CEL.gz	GSE20916	U133_plus2	tumor

280	GSM452660.CEL.gz	GSE20916	U133_plus2	tumor
281	GSM588859.CEL.gz	GSE20916	U133_plus2	tumor
282	GSM588862.CEL.gz	GSE20916	U133_plus2	tumor
283	GSM662639.CEL.gz	GSE20916	U133_plus2	tumor
284	GSM662689.CEL.gz	GSE20916	U133_plus2	tumor
285	GSM662717.CEL.gz	GSE20916	U133_plus2	tumor
286	GSM820048.CEL.gz	GSE33113	U133_plus2	tumor
287	GSM820051.CEL.gz	GSE33113	U133_plus2	tumor
288	GSM820057.CEL.gz	GSE33113	U133_plus2	tumor
289	GSM820064.CEL.gz	GSE33113	U133_plus2	tumor
290	GSM820065.CEL.gz	GSE33113	U133_plus2	tumor
291	GSM820074.CEL.gz	GSE33113	U133_plus2	tumor
292	GSM820080.CEL.gz	GSE33113	U133_plus2	tumor
293	GSM820098.CEL.gz	GSE33113	U133_plus2	tumor
294	GSM820110.CEL.gz	GSE33113	U133_plus2	tumor
295	GSM820124.CEL.gz	GSE33113	U133_plus2	tumor
296	GSM820125.CEL.gz	GSE33113	U133_plus2	tumor
297	GSM820129.CEL.gz	GSE33113	U133_plus2	tumor
298	GSM452557.CEL	GSE18105	U133_plus2	tumor LCM
299	GSM452559.CEL	GSE18105	U133_plus2	tumor LCM
300	GSM452561.CEL	GSE18105	U133_plus2	tumor LCM
301	GSM452563.CEL	GSE18105	U133_plus2	tumor LCM
302	GSM452564.CEL	GSE18105	U133_plus2	tumor LCM
303	GSM452569.CEL	GSE18105	U133_plus2	tumor LCM
304	GSM452570.CEL	GSE18105	U133_plus2	tumor LCM
305	GSM452582.CEL	GSE18105	U133_plus2	tumor LCM
306	GSM452583.CEL	GSE18105	U133_plus2	tumor LCM
307	GSM452585.CEL	GSE18105	U133_plus2	tumor LCM
308	GSM452586.CEL	GSE18105	U133_plus2	tumor LCM
309	GSM452587.CEL	GSE18105	U133_plus2	tumor LCM
310	GSM452592.CEL	GSE18105	U133_plus2	tumor LCM
311	GSM452607.CEL	GSE18105	U133_plus2	tumor LCM
312	GSM452608.CEL	GSE18105	U133_plus2	tumor LCM
313	GSM452609.CEL	GSE18105	U133_plus2	tumor LCM
314	GSM452614.CEL	GSE18105	U133_plus2	tumor LCM
315	GSM452620.CEL	GSE18105	U133_plus2	tumor LCM
316	GSM452622.CEL	GSE18105	U133_plus2	tumor LCM
317	GSM523242_Microdys_11AN.CEL	GSE20916	U133_plus2	normal microdissected
318	GSM523243_Microdys_13AN.CEL	GSE20916	U133_plus2	normal microdissected
319	GSM523246_Microdys_20AN.CEL	GSE20916	U133_plus2	normal microdissected
320	GSM523268_Microdys_54ANN.CEL	GSE20916	U133_plus2	normal microdissected
321	GSM523269_Microdys_55ANN.CEL	GSE20916	U133_plus2	normal microdissected
322	GSM523270_Microdys_56ANN.CEL	GSE20916	U133_plus2	normal microdissected
323	GSM523271_Microdys_58ANN.CEL	GSE20916	U133_plus2	normal microdissected
324	GSM523273_Microdys_9AN.CEL	GSE20916	U133_plus2	normal microdissected
325	GSM523274_Microdys_23AN.CEL	GSE20916	U133_plus2	normal microdissected
326	GSM523280_Microdys_59ANN.CEL	GSE20916	U133_plus2	normal microdissected
327	GSM523245_Microdys_1EN.CEL	GSE20916	U133_plus2	normal microdissected crypt
328	GSM523255_Microdys_32EN.CEL	GSE20916	U133_plus2	normal microdissected crypt
329	GSM523260_Microdys_37EN.CEL	GSE20916	U133_plus2	normal microdissected crypt
330	GSM523264_Microdys_49ENN.CEL	GSE20916	U133_plus2	normal microdissected crypt
331	GSM523265_Microdys_50ENN.CEL	GSE20916	U133_plus2	normal microdissected crypt
332	GSM523266_Microdys_51ENN.CEL	GSE20916	U133_plus2	normal microdissected crypt
333	GSM523267_Microdys_52ENN.CEL	GSE20916	U133_plus2	normal microdissected crypt
334	GSM523272_Microdys_6EN.CEL	GSE20916	U133_plus2	normal microdissected crypt
335	GSM523276_Microdys_40EN.CEL	GSE20916	U133_plus2	normal microdissected crypt
336	GSM523279_Microdys_53ENN.CEL	GSE20916	U133_plus2	normal microdissected crypt
337	GSM523248_Microdys_22AC.CEL	GSE20916	U133_plus2	tumor microdissected
338	GSM523249_Microdys_24AC.CEL	GSE20916	U133_plus2	tumor microdissected
339	GSM523250_Microdys_26AC.CEL	GSE20916	U133_plus2	tumor microdissected
340	GSM523253_Microdys_30AC.CEL	GSE20916	U133_plus2	tumor microdissected
341	GSM523275_Microdys_28AC.CEL	GSE20916	U133_plus2	tumor microdissected
342	GSM523257_Microdys_34EC.CEL	GSE20916	U133_plus2	tumor microdissected crypt
343	GSM523258_Microdys_35EC.CEL	GSE20916	U133_plus2	tumor microdissected crypt
344	GSM523262_Microdys_39EC.CEL	GSE20916	U133_plus2	tumor microdissected crypt
345	GSM523263_Microdys_42EC.CEL	GSE20916	U133_plus2	tumor microdissected crypt
346	GSM523278_Microdys_43EC.CEL	GSE20916	U133_plus2	tumor microdissected crypt
347	GSM368860.CEL	GSE14773	U133_plus2	Cells
348	GSM368863.CEL	GSE14773	U133_plus2	Cells
349	GSM368864.CEL	GSE14773	U133_plus2	Cells
350	GSM368865.CEL	GSE14773	U133_plus2	Cells

351	GSM368866.CEL	GSE14773	U133_plus2	Cells
352	GSM368867.CEL	GSE14773	U133_plus2	Cells
353	GSM368868.CEL	GSE14773	U133_plus2	Cells
354	GSM368869.CEL	GSE14773	U133_plus2	Cells
355	GSM571781.CEL.gz	GSE23295	U133_plus2	Cells
356	GSM571782.CEL.gz	GSE23295	U133_plus2	Cells
357	GSM571783.CEL.gz	GSE23295	U133_plus2	Cells
358	GSM571784.CEL.gz	GSE23295	U133_plus2	Cells
359	GSM610544.CEL	GSE24795	U133_plus2	Cells
360	GSM610545.CEL	GSE24795	U133_plus2	Cells
361	GSM610546.CEL	GSE24795	U133_plus2	Cells
362	GSM610547.CEL	GSE24795	U133_plus2	Cells
363	GSM610548.CEL	GSE24795	U133_plus2	Cells
364	GSM610549.CEL	GSE24795	U133_plus2	Cells
365	GSM610550.CEL	GSE24795	U133_plus2	Cells
366	GSM610551.CEL	GSE24795	U133_plus2	Cells
367	GSM610552.CEL	GSE24795	U133_plus2	Cells
368	GSM610553.CEL	GSE24795	U133_plus2	Cells
369	GSM610554.CEL	GSE24795	U133_plus2	Cells
370	GSM610555.CEL	GSE24795	U133_plus2	Cells
371	GSM610556.CEL	GSE24795	U133_plus2	Cells
372	GSM610557.CEL	GSE24795	U133_plus2	Cells
373	GSM610558.CEL	GSE24795	U133_plus2	Cells
374	GSM610559.CEL	GSE24795	U133_plus2	Cells
375	GSM610560.CEL	GSE24795	U133_plus2	Cells
376	GSM610561.CEL	GSE24795	U133_plus2	Cells
377	GSM610562.CEL	GSE24795	U133_plus2	Cells
378	GSM610563.CEL	GSE24795	U133_plus2	Cells
379	GSM610564.CEL	GSE24795	U133_plus2	Cells
380	GSM610565.CEL	GSE24795	U133_plus2	Cells
381	GSM610566.CEL	GSE24795	U133_plus2	Cells
382	GSM610567.CEL	GSE24795	U133_plus2	Cells
383	GSM610568.CEL	GSE24795	U133_plus2	Cells
384	GSM610569.CEL	GSE24795	U133_plus2	Cells
385	GSM610570.CEL	GSE24795	U133_plus2	Cells
386	GSM610571.CEL	GSE24795	U133_plus2	Cells
387	GSM610572.CEL	GSE24795	U133_plus2	Cells
388	GSM610573.CEL	GSE24795	U133_plus2	Cells
389	GSM843481_CACO2_1_PFIZER.CEL	GSE34211	U133_plus2	Cells
390	GSM843482_CACO2_3_PFIZER.CEL	GSE34211	U133_plus2	Cells
391	GSM843490_COLO205_1_Good_NCI50_WYETH.CEL	GSE34211	U133_plus2	Cells
392	GSM843491_COLO320HSR_1_PFIZER.CEL	GSE34211	U133_plus2	Cells
393	GSM844550_HCC2998_1_Good_NCI50_WYETH.CEL	GSE34211	U133_plus2	Cells
394	GSM844551_HCC2998_2_Good_NCI50_WYETH.CEL	GSE34211	U133_plus2	Cells
395	GSM844554_HCT15_1_PFIZER.CEL	GSE34211	U133_plus2	Cells
396	GSM844576_KM12_1_Good_NCI50_WYETH.CEL	GSE34211	U133_plus2	Cells
397	GSM844577_KM12_2_Good_NCI50_WYETH.CEL	GSE34211	U133_plus2	Cells
398	GSM844580_LS123_1_PFIZER.CEL	GSE34211	U133_plus2	Cells
399	GSM844581_LS153_1_PFIZER.CEL	GSE34211	U133_plus2	Cells
400	GSM844676_RKO_1_PFIZER.CEL	GSE34211	U133_plus2	Cells
401	GSM844704_SNUC1_1_PFIZER.CEL	GSE34211	U133_plus2	Cells
402	GSM844713_SW48_1_PFIZER.CEL	GSE34211	U133_plus2	Cells
403	GSM869232.cel.gz	GSE35478	U133_plus2	Cells
404	GSM869233.CEL.gz	GSE35478	U133_plus2	Cells
405	GSM869234.cel.gz	GSE35478	U133_plus2	Cells
406	GSM869235.CEL.gz	GSE35478	U133_plus2	Cells
407	GSM869236.CEL.gz	GSE35478	U133_plus2	Cells
408	GSM869237.CEL.gz	GSE35478	U133_plus2	Cells
409	GSM869238.cel.gz	GSE35478	U133_plus2	Cells
410	GSM869239.CEL.gz	GSE35478	U133_plus2	Cells
411	GSM869240.cel.gz	GSE35478	U133_plus2	Cells
412	GSM869241.CEL.gz	GSE35478	U133_plus2	Cells
413	GSM869242.CEL.gz	GSE35478	U133_plus2	Cells
414	GSM869243.CEL.gz	GSE35478	U133_plus2	Cells
415	GSM869244.CEL.gz	GSE35478	U133_plus2	Cells
416	GSM869245.CEL.gz	GSE35478	U133_plus2	Cells
417	GSM869246.CEL.gz	GSE35478	U133_plus2	Cells
418	GSM870721_JMM.HG_U133_Plus_2.5212_RKO_E1C.CEL	GSE35566	U133_plus2	Cells
419	GSM870722_JMM.HG_U133_Plus_2.5214_RKO_E2C.CEL	GSE35566	U133_plus2	Cells
420	GSM870723_JMM.HG_U133_Plus_2.5216_SW48_E2C.CEL	GSE35566	U133_plus2	Cells
421	GSM870724_JMM.HG_U133_Plus_2.5218_HT29_E2C.CEL	GSE35566	U133_plus2	Cells

422	GSM870725_JMM.HG_U133_Plus_2.5220_T84_E1C.CEL	GSE35566	U133_plus2	Cells
423	GSM870726_JMM.HG_U133_Plus_2.5222_CACO_E2C.CEL	GSE35566	U133_plus2	Cells
424	GSM870727_JMM.HG_U133_Plus_2.5418_HCT116_E2C.CEL	GSE35566	U133_plus2	Cells
425	GSM870728_JMM.HG_U133_Plus_2.5946_SKE_E1C.CEL	GSE35566	U133_plus2	Cells
426	GSM870729_JMM.HG_U133_Plus_2.5948_SKE_E2C.CEL	GSE35566	U133_plus2	Cells
427	GSM870730_JMM.HG_U133_Plus_2.5950_LME_E1C.CEL	GSE35566	U133_plus2	Cells
428	GSM870731_JMM.HG_U133_Plus_2.5952_LME_E2C.CEL	GSE35566	U133_plus2	Cells
429	GSM870732_JMM.HG_U133_Plus_2.5958_HCE_E1C.CEL	GSE35566	U133_plus2	Cells
430	GSM870733_JMM.HG_U133_Plus_2.5960_HCE_E2C.CEL	GSE35566	U133_plus2	Cells
431	GSM870734_JMM.HG_U133_Plus_2.5962_LSE_E1C.CEL	GSE35566	U133_plus2	Cells
432	GSM870735_JMM.HG_U133_Plus_2.5964_LSE_E2C.CEL	GSE35566	U133_plus2	Cells
433	GSM870736_LA.HG_U133_Plus_2.4906_CACO_E1C.CEL	GSE35566	U133_plus2	Cells
434	GSM870737_LA.HG_U133_Plus_2.4908_HT29_E1C.CEL	GSE35566	U133_plus2	Cells
435	GSM870738_LA.HG_U133_Plus_2.4910_HCT116_E1C.CEL	GSE35566	U133_plus2	Cells
436	GSM870739_LA.HG_U133_Plus_2.4912_SW48_E1C.CEL	GSE35566	U133_plus2	Cells
437	GSM886897.CEL	GSE36133	U133_plus2	Cells
438	GSM886924.CEL	GSE36133	U133_plus2	Cells
439	GSM886933.CEL	GSE36133	U133_plus2	Cells
440	GSM886934.CEL	GSE36133	U133_plus2	Cells
441	GSM886935.CEL	GSE36133	U133_plus2	Cells
442	GSM886936.CEL	GSE36133	U133_plus2	Cells
443	GSM886940.CEL	GSE36133	U133_plus2	Cells
444	GSM886941.CEL	GSE36133	U133_plus2	Cells
445	GSM886943.CEL	GSE36133	U133_plus2	Cells
446	GSM886968.CEL	GSE36133	U133_plus2	Cells
447	GSM886979.CEL	GSE36133	U133_plus2	Cells
448	GSM887027.CEL	GSE36133	U133_plus2	Cells
449	GSM887057.CEL	GSE36133	U133_plus2	Cells
450	GSM887062.CEL	GSE36133	U133_plus2	Cells
451	GSM887063.CEL	GSE36133	U133_plus2	Cells
452	GSM887105.CEL	GSE36133	U133_plus2	Cells
453	GSM887109.CEL	GSE36133	U133_plus2	Cells
454	GSM887137.CEL	GSE36133	U133_plus2	Cells
455	GSM887141.CEL	GSE36133	U133_plus2	Cells
456	GSM887142.CEL	GSE36133	U133_plus2	Cells
457	GSM887212.CEL	GSE36133	U133_plus2	Cells
458	GSM887274.CEL	GSE36133	U133_plus2	Cells
459	GSM887277.CEL	GSE36133	U133_plus2	Cells
460	GSM887278.CEL	GSE36133	U133_plus2	Cells
461	GSM887279.CEL	GSE36133	U133_plus2	Cells
462	GSM887280.CEL	GSE36133	U133_plus2	Cells
463	GSM887281.CEL	GSE36133	U133_plus2	Cells
464	GSM887303.CEL	GSE36133	U133_plus2	Cells
465	GSM887431.CEL	GSE36133	U133_plus2	Cells
466	GSM887443.CEL	GSE36133	U133_plus2	Cells
467	GSM887445.CEL	GSE36133	U133_plus2	Cells
468	GSM887479.CEL	GSE36133	U133_plus2	Cells
469	GSM887526.CEL	GSE36133	U133_plus2	Cells
470	GSM887541.CEL	GSE36133	U133_plus2	Cells
471	GSM887576.CEL	GSE36133	U133_plus2	Cells
472	GSM887602.CEL	GSE36133	U133_plus2	Cells
473	GSM887612.CEL	GSE36133	U133_plus2	Cells
474	GSM887619.CEL	GSE36133	U133_plus2	Cells
475	GSM887624.CEL	GSE36133	U133_plus2	Cells
476	GSM887632.CEL	GSE36133	U133_plus2	Cells
477	GSM887635.CEL	GSE36133	U133_plus2	Cells
478	GSM887643.CEL	GSE36133	U133_plus2	Cells
479	GSM887644.CEL	GSE36133	U133_plus2	Cells
480	GSM887645.CEL	GSE36133	U133_plus2	Cells
481	GSM887646.CEL	GSE36133	U133_plus2	Cells
482	GSM887664.CEL	GSE36133	U133_plus2	Cells
483	GSM887667.CEL	GSE36133	U133_plus2	Cells
484	GSM887668.CEL	GSE36133	U133_plus2	Cells
485	GSM887673.CEL	GSE36133	U133_plus2	Cells
486	GSM887674.CEL	GSE36133	U133_plus2	Cells
487	GSM887675.CEL	GSE36133	U133_plus2	Cells
488	GSM887677.CEL	GSE36133	U133_plus2	Cells
489	GSM887679.CEL	GSE36133	U133_plus2	Cells
490	GSM887681.CEL	GSE36133	U133_plus2	Cells
491	GSM887686.CEL	GSE36133	U133_plus2	Cells
492	GSM434140.CEL.gz	GSE17375	U133_plus2	CSC

493	GSM434141.CEL.gz	GSE17375	U133_plus2	CSC
494	GSM434142.CEL.gz	GSE17375	U133_plus2	CSC
495	GSM434143.CEL.gz	GSE17375	U133_plus2	CSC
496	GSM820036.CEL.gz	GSE33112	U133_plus2	CSC
497	GSM820037.CEL.gz	GSE33112	U133_plus2	CSC
498	GSM820038.CEL.gz	GSE33112	U133_plus2	CSC
499	GSM820039.CEL.gz	GSE33112	U133_plus2	CSC
500	GSM820040.CEL.gz	GSE33112	U133_plus2	CSC
501	GSM820041.CEL.gz	GSE33112	U133_plus2	CSC
502	GSM820042.CEL.gz	GSE33112	U133_plus2	CSC
503	GSM820043.CEL.gz	GSE33112	U133_plus2	CSC
504	GSM820044.CEL.gz	GSE33112	U133_plus2	CSC
505	GSM820045.CEL.gz	GSE33112	U133_plus2	CSC
506	GSM820046.CEL.gz	GSE33112	U133_plus2	CSC
507	GSM820047.CEL.gz	GSE33112	U133_plus2	CSC

250 selected genes List

Candidate genes (predicted transmembrane or GPI-anchored) differentially upregulated in Stem vs Differentiated CSCs

FC vs Differentiated	Global RankProd average FC between CSCs and differentiated counterparts
FC vs Normal Colon<br>FC vs Normal Colon Microdissected<br>FC vs Normal Colon Microdissected Cryp	Global RankProd average FC between CSCs and bulk normal or microdissected normal colon tissue
FC vs Tumor<br>FC vs Tumor Microdissected LCM<br>FC vs Tumor Microdissected<br>FC vs Tumor Microdissected Crypt	Global RankProdaverage FC between CSCs and bulk or microdissected colon tumor tissue
FC vs Colon Cancer Cell lines	Global RankProd average FC between CSCs and colon cancer cell lines
	In all cases a blank white space indicates that the global average RankProd FC was not considered statistically signficant

			dCSC	NORMAL			TUMOR				CELLS	
HuGene probe	NCBI Gene	NCBI GeneID	FC vs Differentiated CSCs	FC vs Normal Colon	FC vs Normal Colon Microdissected	FC vs Normal Colon Microdissected Crypt	FC vs Tumor	FC vs Tumor LCM	FC vs Tumor Microdissected	FC vs Tumor Microdissected Crypt	FC vs Colon Cancer Cell lines	Description
8140984	HEPACAM2	253012	3,94	-2,46	-3,79	-4,79	4,9	3,85	37,14	11,15	22,99	HEPACAM family member 2
7957140	LGR5	8549	3,29	7,86	9,24	11,63	2,65	3,42	15,59	14,31	5,1	leucine-rich repeat-containing G protein-coupled receptor 5
7903592	KIAA1324	57535	3,22			-3,98	2,44	1,65	2,39		14,6	KIAA1324
8056363	SLC38A11	151258	3,02									solute carrier family 38, member 11
8107100	RGMB	285704	3,01	4,26	4,25		5,58	3,98	4,16	3,16	8,71	RGM domain family, member B
8123446	SMOC2	64094	2,98	13,83	45,32	14,08	13,44	11,93	7,17	3,22	105,39	SPARC related modular calcium binding 2
8073633	PNPLA3	80339	2,62	19,25	15,22	15,05	16,1	10,21	16,02	13,41	6,05	patatin-like phospholipase domain containing 3
8111490	PRLR	5618	2,62	2,2	2,74	2,71	2,12	2,23	2,17	2,53	2,62	prolactin receptor
8020827	MEP1B	4225	2,55				2,61	2,8	2,43	2,76	2,74	meprin A, beta
8123137	ACAT2	39	2,54	4,82	5,71	5,67	4,465	5,05	6,67	5,12	3,93	acetyl-CoA acetyltransferase 2
8084524	EPHB3	2049	2,53	11,78	31,02	26,33	6,33	6,92	28,19	31,73	14,31	EPH receptor B3
7937952	OR51E1	143503	2,46	77,47	68,04	59,68	57,73	25,34	13,14	9,91	92,08	olfactory receptor, family 51, subfamily E, member 1
8059525	TM4SF20	79853	2,42	2,61		2,49	2,27	1,17	-4,06	-3,94	6,7	transmembrane 4 L six family member 20
8162059	SLC28A3	64078	2,38	3,81	3,52	2,68	3,05	2,14	4,48	3,83	4,11	solute carrier family 28 (sodium-coupled nucleoside transporter), member 3
8169836	XPNPEP2	7512	2,38		2,22	2,36	1,695	2,45	2,27	2,69	3,02	X-prolyl aminopeptidase (aminopeptidase P) 2, membrane-bound
8104570	FAM105A	54491	2,3	2,63			3,225	2,39	2,26		10,92	family with sequence similarity 105, member A
8166784	TSPAN7	7102	2,3	-7,18	-10,44	-9,46					3,25	tetraspanin 7
8160504	LRRC19	64922	2,29	-5,81	-7,73	-5,06		1,63			6,35	leucine rich repeat containing 19
8166632	GK	2710	2,1		2,76	4,27			2,55	2,5	2,19	glycerol kinase
8100760	UGT2A3	79799	2,08	-15,77	-28,49	-27,4	-1,56	-1,79			1,5	UDP glucuronosyltransferase 2 family, polypeptide A3
8080511	CACNA1D	776	2,05	1,97	2,44	2,7	1,735	2,02	2,74	2,84	2	calcium channel, voltage-dependent, L type, alpha 1D subunit
7954143	PTPRO	5800	2,04									protein tyrosine phosphatase, receptor type, O
8045795	KCNJ3	3760	2	2,89	3,42	3,19	2,875	2,72	3,3	2,99	2,54	potassium inwardly-rectifying channel, subfamily J, member 3
8099476	PROM1	8842	2	2,79	3,02	2,29	3,31	3,12	2,72	2,18	21,67	prominin 1
7953252	GALNT8	26290	1,99						2,27	2,29	2,38	UDP-N-acetyl-alpha-D-galactosamine:polypeptide N-acetylgalactosaminyltransferase 8 (GalNAc-T8)
7971731	ATP7B	540	1,98	3,42			2,6				3,64	ATPase, Cu++ transporting, beta polypeptide
7961900	ITPR2	3709	1,98	2,2		2,17	2,365	2,29	2,4			inositol 1,4,5-triphosphate receptor, type 2
8103951	ACSL1	2180	1,96	4,19	3,31	2,43	2,635	1,81			2,57	acyl-CoA synthetase long-chain family member 1
7961455	GUCY2C	2984	1,96	2,04	2,96		2,78	2,98			27,82	guanylate cyclase 2C (heat stable enterotoxin receptor)
8057959	PGAP1	80055	1,94	9,86	4,84	3,99	9,175	4,4	3,81	3,87	6,75	post-GPI attachment to proteins 1
8167305	EBP	10682	1,94	3,92	3,2	2,28	3,23	3,16	3,3	2,25	3,02	emopamil binding protein (sterol isomerase)
7950990	SLC36A4	120103	1,93	3,84	2,4		3,985	3,31	4,46	4,6	2,77	solute carrier family 36 (proton/amino acid symporter), member 4
7982757	CASC5	57082	1,92									cancer susceptibility candidate 5
8123739	NRN1	51299	1,88	2,78	2,78	7,63	1,985	3,32	5,31	7,7	3,27	neuritin 1
7947744	LRP4	4038	1,86	1,86			1,925	1,29	4,26	4,79	5,71	low density lipoprotein receptor-related protein 4
7919314	FMO5	2330	1,86	-4,44	-6,93	-4,38			-3,11		2,62	flavin containing monooxygenase 5
8123246	SLC22A3	6581	1,84	11,93	12,95	14,25	3,075	2,15	4,85	6,17	4,51	solute carrier family 22 (extraneuronal monoamine transporter), member 3
7906904	HSD17B7	51478	1,82	3,12	4,66	4,24	2,58	3,81	4,53	3,77	3,1	hydroxysteroid (17-beta) dehydrogenase 7
8056151	PLA2R1	22925	1,82	2,3	2,35		2,215	1,82			2,2	phospholipase A2 receptor 1, 180kDa
8166925	MAOA	4128	1,82	-2,54		-2,54					5,45	monoamine oxidase A
8117900	DDR1	780	1,82	-3,41			-3				-2,25	discoidin domain receptor tyrosine kinase 1
7961279	TAS2R14	50840	1,8	2,31	2,4	2,63	2,445	2,15	2,27	2,41	2,14	taste receptor, type 2, member 14
8175492	ATP11C	286410	1,8	2			1,815	1,77				ATPase, class VI, type 11C
7940323	MS4A8B	83661	1,8				2,345				5,03	membrane-spanning 4-domains, subfamily A, member 8B
7973724	NYNRIN	57523	1,8									NYNRIN
8100026	ATP8A1	10396	1,79	-1,81								ATPase, aminophospholipid transporter (APLT), class I, type 8A, member 1
8175666	GABRE	2564	1,78	2,8	2,93	2,91	1,965	1,47	3,56	3,04	3,26	gamma-aminobutyric acid (GABA) A receptor, epsilon
8121277	AIM1	202	1,78				1,925	1,62			5,59	absent in melanoma 1
8093053	TFRC	7037	1,78							-2,93		transferrin receptor (p90, CD71)
8062041	ACSS2	55902	1,78								3,68	acyl-CoA synthetase short-chain family member 2
8078014	SLC6A6	6533	1,77	4,58	3,49	3,3	2,27	2,37	4,87	5,7		solute carrier family 6 (neurotransmitter transporter, taurine), member 6
7986383	IGF1R	3480	1,77			-3,38						insulin-like growth factor 1 receptor
8178977	TAPBP	6892	1,76			2,19				2,4		TAP binding protein (tapasin)
7897774	CLCN6	1185	1,76	2,01			2,28	1,93				chloride channel 6
8142407	TMEM168	64418	1,75			-2,62					2,11	transmembrane protein 168
8157727	GPR21	2844	1,74								1,67	G protein-coupled receptor 21
8135048	MUC17	140453	1,74	-1,93			-1,49	-2,48			1,36	mucin 17, cell surface associated
7950067	DHCR7	1717	1,73	7,8	5,22	4,29	4,395	4,28			3,17	7-dehydrocholesterol reductase
7917728	FAM69A	388650	1,73					-2,04			1,69	family with sequence similarity 69, member A
7953218	RAD51AP1	10635	1,72	6,46	2,83	2,07	2,88	1,58				RAD51 associated protein 1
8140579	CACNA2D1	781	1,72									calcium channel, voltage-dependent, alpha 2/delta subunit 1
8123006	SYNJ2	8871	1,71	3,68	7,27	3,84	3,26	3,15	4,58	4,42	3,64	synaptojanin 2
8152355	SYBU	55638	1,71	3,51			2,24				3,57	syntabulin (syntaxin-interacting)
8110265	FGFR4	2264	1,7	8,93	11,99	5,76	5,875	7	4,01	5,32	8,15	fibroblast growth factor receptor 4
8090898	STAG1	10274	1,7								1,78	stromal antigen 1
7938629	PDE3B	5140	1,7									phosphodiesterase 3B, cGMP-inhibited
8117140	CDKAL1	54901	1,69	2,75	4,37	3,76	2,155	2,99	3,16	3,58		CDK5 regulatory subunit associated protein 1-like 1
8147548	POP1	10940	1,69	3,93	2,92		2,305	1,77				processing of precursor 1, ribonuclease P/MRP subunit (S. cerevisiae)
8089372	KIAA1524	57650	1,69	2,36							-1,53	KIAA1524

7916432	**DHCR24**	1718	1,69				1,825	1,86				24-dehydrocholesterol reductase
8000346	**ERN2**	10595	1,69								1,84	endoplasmic reticulum to nucleus signaling 2
7973727	**NYNRIN**	57523	1,69									NYN domain and retroviral integrase containing
7915612	**PTCH2**	8643	1,69									PTCH2
8169174	**RNF128**	79589	1,69				1,78				5,79	ring finger protein 128
8101992	**SLC39A8**	64116	1,69			-3,14					2,69	solute carrier family 39 (zinc transporter), member 8
8141708	**CLDN15**	24146	1,68	3,07	4,59	3,94	3,295	2,73	3,08	2,18	2,12	claudin 15
7981798	**TUBGCP5**	114791	1,68	3,73	3,38		3,42	2,73	2,62			tubulin, gamma complex associated protein 5
8141107	**SLC25A13**	10165	1,68	3,43	3,05	3,04	3,545	3,53	3,78	3,75	2,89	solute carrier family 25, member 13 (citrin)
7996772	**SLC7A6**	9057	1,68	2,51			1,785				1,63	solute carrier family 7 (cationic amino acid transporter, y+ system), member 6
7944656	**SC5DL**	6309	1,68	1,91			2,315	1,82			2,11	sterol-C5-desaturase (ERG3 delta-5-desaturase homolog, S. cerevisiae)-like
8039453	**TMEM150B**	284417	1,68									TMEM150B
8081710	**SIDT1**	54847	1,68	-3,51	-5,56	-6,7					2,72	SID1 transmembrane family, member 1
7997962	**DPEP1**	1800	1,67	23,05	30,47	34,52	3,515	6,17	24,86	6,39	10,18	dipeptidase 1 (renal)
7950307	**UCP2**	7351	1,67	1,95	5,43	7,12	1,87	2,78	6,81	6,71	2,82	uncoupling protein 2 (mitochondrial, proton carrier)
7901272	**CYP4X1**	260293	1,67	3,95	4,83	2,1	-1,3	-1,53		-7,17	2,48	cytochrome P450, family 4, subfamily X, polypeptide 1
8106170	**TMEM171**	134285	1,67									TMEM171
8133818	**PHTF2**	57157	1,66	3,19	2,55	2,75	2,32					putative homeodomain transcription factor 2
7999387	**EMP2**	2013	1,66		2,26		2	2,4	4,79	4,3	2,78	epithelial membrane protein 2
8111533	**LMBRD2**	92255	1,66									LMBR1 domain containing 2
8091723	**RARRES1**	5918	1,66	-1,81					3,02	4,13	2,15	retinoic acid receptor responder (tazarotene induced) 1
8049349	**UGT1A1**	54658	1,65	4,88	12,39	15,12	17,76	23,9	79,87	76,86	8,73	UDP glucuronosyltransferase 1 family, polypeptide A1
7940565	**FADS2**	9415	1,65	6,92	8,15	10,98	5,965	6,46	7,12	11,39	5,05	fatty acid desaturase 2
7961285	**TAS2R20**	259295	1,65									TAS2R20
7909628	**FLVCR1**	28982	1,64	7,54	4,41	3,18	4,375	4,24			2,59	feline leukemia virus subgroup C cellular receptor 1
7967736	**POLE**	5426	1,64	1,84			1,925	2,02				polymerase (DNA directed), epsilon
7913566	**HTR1D**	3352	1,64								-1,35	5-hydroxytryptamine (serotonin) receptor 1D
7986359	**IGF1R**	3480	1,64									IGF1R
8148280	**SQLE**	6713	1,63	5,18	2,42	2,24	2,27	1,81			2,38	squalene epoxidase
7964602	**LRIG3**	121227	1,63			-4,53					1,86	leucine-rich repeats and immunoglobulin-like domains 3
8137526	**INSIG1**	3638	1,62	3,94	2,5	2,79	3,195	2,79			4,17	insulin induced gene 1
7997593	**ATP2C2**	9914	1,62				2,045				5,63	ATPase, Ca++ transporting, type 2C, member 2
7961281	**TAS2R50**	259296	1,62									taste receptor, type 2, member 50
7965964	**SLC41A2**	84102	1,62	-2,04	-6,21	-3,84						solute carrier family 41, member 2
8000205	**NPIPL3**	100132247	1,61									NPIPL3
8161865	**PRUNE2**	158471	1,61								12,01	prune homolog 2 (Drosophila)
7943605	**ACAT1**	38	1,61									acetyl-CoA acetyltransferase 1
7961291	**TAS2R31**	259290	1,61									TAS2R31
8166723	**XK**	7504	1,61	-1,86	-4,12	-3,82					2,6	X-linked Kx blood group (McLeod syndrome)
8156770	**GALNT12**	79695	1,61	-2,91	-5,66	-4,55					2,06	UDP-N-acetyl-alpha-D-galactosamine:polypeptide N-acetylgalactosaminyltransferase 12 (GalNAc-T12)
8089015	**PROS1**	5627	1,61	-10,83	-30,58	-32,05	-6,525	-9,58		-3,88	-3,38	protein S (alpha)
8056102	**CD302**	9936	1,6				1,85				6,38	CD302 molecule
8110620	**BTNL3**	10917	1,6	-3,36	-3,45	-2,3						butyrophilin-like 3
8056572	**SPC25**	57405	1,59	14,65	15,2	7,85	4,68	3,91	4,97	3,34	2,15	SPC25, NDC80 kinetochore complex component, homolog (S. cerevisiae)
7954985	**TMEM117**	84216	1,59									transmembrane protein 117
7918323	**SORT1**	6272	1,59									sortilin 1
7932243	**FAM171A1**	221061	1,59				1,9	2,1			4,24	family with sequence similarity 171, member A1
7903144	**SLC44A3**	126969	1,59			-3,07			-3,18	-2,99	2,01	solute carrier family 44, member 3
8113491	**STARD4**	134429	1,58	3,8			4,17	2,85	2,3		3,11	StAR-related lipid transfer (START) domain containing 4
7924636	**TMEM63A**	9725	1,58								2,07	transmembrane protein 63A
8002629	**PKD1L3**	342372	1,58									PKD1L3, polycystin protein family.
8098084	**ETFDH**	2110	1,58				2,02				2,38	electron-transferring-flavoprotein dehydrogenase
8108737	**PCDHB13**	56123	1,58									protocadherin beta 13
7961287	**TAS2R19**	259294	1,58									TAS2R19
8094938	**NIPAL1**	152519	1,58	-1,89								NIPA-like domain containing 1
8113369	**SLCO4C1**	353189	1,58		-4,92	-6,18						solute carrier organic anion transporter family, member 4C1
8091811	**SI**	6476	1,58	-29,76	-71,94	-69,93	-2,215	-3,59			1,83	sucrase-isomaltase (alpha-glucosidase)
7972217	**SPRY2**	10253	1,57	2,19			2,28	2,4	2,59	2,56	4,39	sprouty homolog 2 (Drosophila)
7955441	**METTL7A**	25840	1,57	-2,17				1,83			2,22	methyltransferase like 7A
7948612	**FADS1**	3992	1,56	11,11	11,44	18,5	6,105	5,85	2,94	2,84	4,78	fatty acid desaturase 1
8130720	**SFT2D1**	113402	1,56	2,69	3,05	2,55	2,775	2,1	2,93	2,41	1,87	SFT2 domain containing 1
7920472	**TPM3**	7170	1,56	2,79	2,64		2,54	1,89				tropomyosin 3
8088642	**LRIG1**	26018	1,56		2,5		1,97				4,37	leucine-rich repeats and immunoglobulin-like domains 1
7951717	**TMPRSS5**	80975	1,56	2,35		2,33	2,215	2,02			2,13	transmembrane protease, serine 5
8135661	**CFTR**	1080	1,56	1,78			2,095	2,27		-3,31	16,98	cystic fibrosis transmembrane conductance regulator (ATP-binding cassette sub-family C, member 7)
7917052	**SLC44A5**	204962	1,56					1,74				solute carrier family 44, member 5
8009476	**MAP2K6**	5608	1,56				1,98	1,88			4,43	mitogen-activated protein kinase kinase 6
7930148	**SFXN2**	118980	1,56								1,72	sideroflexin 2
8045768	**ARL6IP6**	151188	1,55	3,08			2,645	1,88			2	ADP-ribosylation-like factor 6 interacting protein 6
8135341	**CDHR3**	222256	1,55	2,41	2,6		2,475	2,7	3,13	3,02	2,54	cadherin-related family member 3
8107769	**SLC12A2**	6558	1,54	3,07	2,77						2,66	solute carrier family 12 (sodium/potassium/chloride transporters), member 2
7950796	**CREBZF**	58487	1,54	2,67			2,09					CREB/ATF bZIP transcription factor
8080562	**IL17RB**	55540	1,54	1,75	2,48	2,03					1,93	interleukin 17 receptor B
8052979	**SFXN5**	94097	1,54				2,175	2,42			2,3	sideroflexin 5
8059538	**SLC19A3**	80704	1,54						-5,66		1,47	solute carrier family 19, member 3
7937016	**CLRN3**	119467	1,54				2,13	1,85			12,85	clarin 3
8087224	**SLC25A20**	788	1,54	-2,9								solute carrier family 25 (carnitine/acylcarnitine translocase), member 20
8152522	**ENPP2**	5168	1,54	-6,14	-3,77		-3,175	-2			1,84	ectonucleotide pyrophosphatase/phosphodiesterase 2
8131143	**LFNG**	3955	1,53	2,82	3,31	4	2,71	2,89	3,64	3,8	2,62	LFNG O-fucosylpeptide 3-beta-N-acetylglucosaminyltransferase
7938683	**OR7E14P**	10819	1,53				2,75	2,24			2,13	olfactory receptor, family 7, subfamily E, member 14 pseudogene
7909866	**02-mar**	54996	1,53									MOCO sulphurase C-terminal domain containing 2
7957167	**TMEM19**	55266	1,53		-2,9	-2,41						transmembrane protein 19
8093166	**PIGZ**	80235	1,53	-3,47	-3,95	-4,1					2,81	phosphatidylinositol glycan anchor biosynthesis, class Z
8095402	**UGT2A3**	#N/D	1,52									UGT2A3
8140814	**SLC25A40**	55972	1,52									solute carrier family 25, member 40
8078784	**XYLB**	9942	1,52								-1,66	xylulokinase homolog (H. influenzae)
7954631	**FAR2**	55711	1,52						2,53		2,86	fatty acyl CoA reductase 2
8077731	**FANCD2**	2177	1,52								-1,37	Fanconi anemia, complementation group D2
8060813	**MCM8**	84515	1,52									minichromosome maintenance complex component 8
7974920	**SYNE2**	23224	1,52	-1,91		-3,12					1,67	spectrin repeat containing, nuclear envelope 2
7924603	**LBR**	3930	1,51	1,99			1,73				2,17	lamin B receptor
7901316	**SLC5A9**	200010	1,51						-8,59	-7,16		solute carrier family 5 (sodium/glucose cotransporter), member 9
8106280	**HMGCR**	3156	1,51				2,31				2,74	3-hydroxy-3-methylglutaryl-CoA reductase
8120967	**NT5E**	4907	1,51								1,63	5'-nucleotidase, ecto (CD73)
7961275	**TAS2R13**	50838	1,51									taste receptor, type 2, member 13
7984704	**NEO1**	4756	1,51	-2,09		-2,37					1,75	neogenin 1
8129837	**IL20RA**	53832	1,51			-2,87		-1,82				interleukin 20 receptor, alpha
7913869	**STMN1**	3925	1,5	2,25								stathmin 1
7964872	**PTPRB**	5787	1,5							2,71	2,64	protein tyrosine phosphatase, receptor type, B
7984524	**PAQR5**	54852	1,5				1,95	1,94				progestin and adipoQ receptor family member V
8081548	**PVRL3**	25945	1,5	-4,34	-5,86	-7,04	-1,995	-2,52	-5,32	-4,83	-1,47	poliovirus receptor-related 3
7916282	**LRP8**	7804	1,49	7,24	8,15	8,66	3,03	3,13	4,8	4,26	2,1	low density lipoprotein receptor-related protein 8, apolipoprotein e receptor
8013989	**SLC6A4**	6532	1,49									solute carrier family 6 (neurotransmitter transporter, serotonin), member 4
8172813	**FAM156A**	727866	1,49									FAM156A
8083839	**GPR160**	26996	1,49								3,01	G protein-coupled receptor 160
7961295	**TAS2R43**	259289	1,49									TAS2R43
7912112	**DNAJC11**	55735	1,48		3,03	3,59		1,8	2,77	3,46		DnaJ (Hsp40) homolog, subfamily C, member 11
7929344	**GPR120**	338557	1,48									GPR120
7901993	**CACHD1**	57685	1,47	3,75		4,7	3,135	2,27		3,82	2,32	cache domain containing 1
7898809	**EPHB2**	2048	1,46	5,34	14,28	7,51	3,26	6,3	5,19	7,52	7,05	EPH receptor B2
7928695	**FAM213A**	84293	1,46		6,17	5,05		3,1	4,26	3,09	2,44	chromosome 10 open reading frame 58
8172425	**SLC38A5**	92745	1,46	2,94	3,5	3	1,3	2,02			1,78	solute carrier family 38, member 5
8066407	**FITM2**	128486	1,46	2,36	2,65	2,31	1,785	1,96				fat storage-inducing transmembrane protein 2

7968678	**FREM2**	341640	1,46									FREM2
8173941	**TSPAN6**	7105	1,46				-1,815					tetraspanin 6
8155083	**CA9**	768	1,45	4,59	4,58	5,06	3,195	1,71	6,25	6,9	3,64	carbonic anhydrase IX
8082846	**EPHB1**	2047	1,45	3,83	4,6	4,81	2,94	3,52	4,94	5,16	2,92	EPH receptor B1
8083978	**NAALADL2**	254827	1,45								1,63	N-acetylated alpha-linked acidic dipeptidase-like 2
7932407	**ST8SIA6**	338596	1,45									ST8SIA6
8167973	**HEPH**	9843	1,45	-5,68	-8,79	-10,35	-3,79	-3,19	-5,59	-6,26	3,42	hephaestin
7949619	**SLC29A2**	3177	1,44	2,82	3,18	2,92	2,34	2,67	2,52	3,01	2,05	solute carrier family 29 (nucleoside transporters), member 2
8151223	**SLCO5A1**	81796	1,44	2,64	2,68		2,39	2,27		2,58	2,36	solute carrier organic anion transporter family, member 5A1
7907702	**SOAT1**	6646	1,44								1,69	sterol O-acyltransferase 1
8151952	**NIPAL2**	79815	1,44									NIPA-like domain containing 2
7997491	**HSD17B2**	3294	1,43	-16,58	-14,58	-4,73	-1,905				1,58	hydroxysteroid (17-beta) dehydrogenase 2
7982938	**TYRO3**	7301	1,42	2,32			1,865	2,1	2,42			TYRO3 protein tyrosine kinase
8108744	**PCDHB14**	56122	1,42	1,89				-2,59	-6,5	-6,35	1,51	protocadherin beta 14
7917954	**FRRS1**	391059	1,42									FRRS1
8006415	**TMEM98**	26022	1,42	-2,04						2,28	2,42	transmembrane protein 98
8081488	**HHLA2**	11148	1,41	-12,24	-10,96	-3,58	-2,81	-2,13	-4,13			HERV-H LTR-associating 2
8126153	**KCNK5**	8645	1,4								2,02	potassium channel, subfamily K, member 5
8132118	**AQP1**	358	1,4	-13,53	-7,11	-11,61	-6,955	-4,04	-3,42			aquaporin 1 (Colton blood group)
8068383	**CLIC6**	54102	1,39									chloride intracellular channel 6
8129254	**MAN1A1**	4121	1,39	-3,13	-5,88	-3,64	-1,875					mannosidase, alpha, class 1A, member 1
8089011	**PROS1**	5627	1,39	-11,95	-33,78	-35,34	-7,19	-10,57		-4,28	-3,74	protein S (alpha)
7951703	**DRD2**	1813	1,38	3,88	4,48	4,46	3,88	3,56	4,7	5,55	4,71	dopamine receptor D2
8151559	**SLC10A5**	347051	1,38									SLC10A5
8078196	**KCNH8**	131096	1,37	6,75	6,33	4,83	4,315	2,57	6,94	5,18	5,73	potassium voltage-gated channel, subfamily H (eag-related), member 8
8092241	**KCNMB3**	27094	1,37	1,85		2,52	1,81					potassium large conductance calcium-activated channel, subfamily M beta member 3
8148220	**FER1L6**	654463	1,36									FER1L6
7972428	**OXGR1**	27199	1,35	6,95	9,78	8,95	4,085	4,47	8,43	11,23	6,38	oxoglutarate (alpha-ketoglutarate) receptor 1
8041582	**PKDCC**	91461	1,35				1,365				3,43	protein kinase domain containing, cytoplasmic homolog (mouse)
8022692	**DSC3**	1825	1,34	1,65	1,89	1,95	-1,32		-7,42	-4,77	-1,03	desmocollin 3
8042310	**SLC1A4**	6509	1,34	2,55	4,2	4,42	2,2	2,03	3,33	3,95	2,02	solute carrier family 1 (glutamate/neutral amino acid transporter), member 4
8042310	**SLC1A4**	6509	1,34	2,55	4,2	4,42	2,2	2,03	3,33	3,95	2,02	solute carrier family 1 (glutamate/neutral amino acid transporter), member 4
8110932	**SEMA5A**	9037	1,33				-1,72					sema domain, seven thrombospondin repeats (type 1 and type 1-like), (semaphorin) 5A
8116537	**BTNL8**	79908	1,33									BTNL8
7909877	**01-mar**	64757	1,33									MOCO sulphurase C-terminal domain containing 1
7947590	**SYT13**	57586	1,32				2,55	1,46			10,23	synaptotagmin XIII
8022118	**EPB41L3**	23136	1,32	-12,12	-6,96	-4,17	-2,76	-2,13			1,49	erythrocyte membrane protein band 4.1-like 3
7989501	**CA12**	771	1,32	-9,23	-6,77	-8,12	-2,415				2,42	carbonic anhydrase XII
8149592	**SLC18A1**	6570	1,31	5,52	6,75	4,04	6,21	6,44	9,78	7,76	8,78	solute carrier family 18 (vesicular monoamine), member 1
8131666	**ITGB8**	3696	1,31	2,56	2,49	2,33	2,115				1,93	integrin, beta 8
8144786	**SLC7A2**	6542	1,31									solute carrier family 7 (cationic amino acid transporter, y+ system), member 2
8009075	**TANC2**	26115	1,31									tetratricopeptide repeat, ankyrin repeat and coiled-coil containing 2
8110183	**CDHR2**	54825	1,31	-4,81	-3,01						1,52	cadherin-related family member 2
7997504	**CDH13**	1012	1,3			2,08					2,3	cadherin 13, H-cadherin (heart)
8114572	**HBEGF**	1839	1,3								2,27	heparin-binding EGF-like growth factor
8123388	**UNC93A**	54346	1,3									UNC93A
8117304	**SLC17A4**	10050	1,3	-4,96	-7,98	-2,87					1,93	solute carrier family 17 (sodium phosphate), member 4
8072587	**SLC5A1**	6523	1,28	2,84			1,585				5,99	solute carrier family 5 (sodium/glucose cotransporter), member 1
7914180	**SPCS2**	9789	1,28									SPCS2
8054451	**EDAR**	10913	1,27	2,32		2,23	1,54		2,4		1,76	ectodysplasin A receptor
7934898	**ANKRD22**	118932	1,27								1,98	ankyrin repeat domain 22
8136557	**TBXAS1**	6916	1,26	2,9	3,62	5,2	1,77	1,76	2,36	3,5	7,91	thromboxane A synthase 1 (platelet)
8163383	**SUSD1**	64420	1,21	2,23			2,005				2,45	sushi domain containing 1
8135015	**MUC3A**	4584	1,21	-2,37			-1,87				1,99	mucin 3A, cell surface associated
8150722	**ST18**	9705	1,19	10,27	8,79	4,57	11,98	8,51	11,15	12,43	12,34	suppression of tumorigenicity 18 (breast carcinoma) (zinc finger protein)
7970793	**SLC46A3**	283537	1,19									SLC46A3
8127145	**ELOVL5**	60481	1,18	4,31	2,96	5,73	2	1,68			4,56	ELOVL family member 5, elongation of long chain fatty acids (FEN1/Elo2, SUR4/Elo3-like, yeast)
8045336	**GPR39**	2863	1,18		3,46	3,72	1,67		3,11	2,62		G protein-coupled receptor 39
8098204	**CPE**	1363	1,17	-2		-2,82				2,2	5,05	carboxypeptidase E
7922029	**GPA33**	10223	1,17	-8,73	-13,99	-9,82	-3,515	-2,57	-9,63	-8,8	1,74	glycoprotein A33 (transmembrane)
8047174	**SLC39A10**	57181	1,15	2,85				-2,06	-4,36	-4,48		solute carrier family 39 (zinc transporter), member 10
7920642	**MUC1**	4582	1,15	-6,14	-5,07	-5,73	-3,185	-2,94			1,89	mucin 1, cell surface associated
8120088	**MEP1A**	4224	1,14	-6,39	-6,95	-4,73	-1,77	1,15	-7,87	-6,29	11,58	meprin A, alpha (PABA peptide hydrolase)
7954330	**SLCO1B3**	28234	1,11	2	2,35	2,32	-2,46	-2,89	-10,49	-12,9	-5,76	solute carrier organic anion transporter family, member 1B3
7983478	**C15orf48**	84419	1,09	-25,32	-32,15	-26,95	-10,69	-7,95	-12,76	-10,06	1,73	chromosome 15 open reading frame 48
8083494	**MME**	4311	1,02	1,73		3,42	1,32	1,4		2,52	1,49	membrane metallo-endopeptidase
7972297	**ABCC4**	10257	1,02								-2,39	ATP-binding cassette, sub-family C (CFTR/MRP), member 4
8108693	**PCDHB4**	56131	1,01	2,37	2,55	2,87	2,475	2,49	2,47	2,51	2,39	protocadherin beta 4
8108693	**PCDHB4**	56131	1,01	2,37	2,55	2,87	2,475	2,49	2,47	2,51	2,39	protocadherin beta 4

103 selected genes List

1st selection	103 candidates (predicted transmembrane or GPI-anchored) were selected as the top genes differentially upregulated in Stem vs Differentiated CSCs and upregulated in CSCs vs normal colon tissues (bulk and/or microdissected)

FC vs Differentiated	Global RankProd average FC between CSCs and differentiated counterparts
FC vs Normal Colon<br>FC vs Normal Colon Microdissected<br>FC vs Normal Colon Microdissected Crypt	Global RankProd average FC between CSCs and bulk normal or microdissected normal colon tissue
FC vs Tumor<br>FC vs Tumor Microdissected LCM<br>FC vs Tumor Microdissected<br>FC vs Tumor Microdissected Crypt	Global RankProdaverage FC between CSCs and bulk or microdissected colon tumor tissue
FC vs Colon Cancer Cell lines	Global RankProd average FC between CSCs and colon cancer cell lines
	In all cases a blank white space indicates that the global average RankProd FC was not considered statistically signficant

				dCSC	NORMAL			TUMOR				CELLS	
Ranking	HuGene probe	NCBI Gene	NCBI Gene ID	FC vs Differentiated	FC vs Normal Colon	FC vs Normal Colon Microdissected	FC vs Normal Colon Microdissected Crypt	FC vs Tumor	FC vs Tumor LCM	FC vs Tumor Microdissected	FC vs Tumor Microdissected Crypt	FC vs Colon Cancer Cell lines	Description
C	7969288	OLFM4	10562	4,3	8,09			4,50	5,09	25,51	4,78	411,95	olfactomedin 4
C	7957140	LGR5	8549	3,29	7,86	9,24	11,63	2,65	3,42	15,59	14,31	5,1	leucine-rich repeat-containing G protein-coupled receptor 5
1	8107100	RGMB	285704	3,01	4,26	4,25		5,58	3,98	4,16	3,16	8,71	RGM domain family, member B
2	8123446	SMOC2	64094	2,98	13,83	45,32	14,08	13,44	11,93	7,17	3,22	105,39	SPARC related modular calcium binding 2
3	8073633	PNPLA3	80339	2,62	19,25	15,22	15,05	16,10	10,21	16,02	13,41	6,05	patatin-like phospholipase domain containing 3
4	8111490	PRLR	5618	2,62	2,2	2,74	2,71	2,12	2,23	2,17	2,53	2,62	prolactin receptor
5	8123137	ACAT2	39	2,54	4,82	5,71	5,67	4,47	5,05	6,67	5,12	3,93	acetyl-CoA acetyltransferase 2
C	8084524	EPHB3	2049	2,53	11,78	31,02	26,33	6,33	6,92	28,19	31,73	14,31	EPH receptor B3
6	7937952	OR51E1	143503	2,46	77,47	68,04	59,68	57,73	25,34	13,14	9,91	92,08	olfactory receptor, family 51, subfamily E, member 1
7	8059525	TM4SF20	79853	2,42	2,61		2,49	2,27	1,17	-4,06	-3,94	6,7	transmembrane 4 L six family member 20
8	8162059	SLC28A3	64078	2,38	3,81	3,52	2,68	3,05	2,14	4,48	3,83	4,11	solute carrier family 28 (sodium-coupled nucleoside transporter), member 3
9	8169836	XPNPEP2	7512	2,38		2,22	2,36	1,70	2,45	2,27	2,69	3,02	X-prolyl aminopeptidase (aminopeptidase P) 2, membrane-bound
10	8104570	FAM105A	54491	2,3	2,63			3,23	2,39	2,26		10,92	family with sequence similarity 105, member A

11	8166632	GK	2710	2,1		2,76	4,27			2,55	2,5	2,19	glycerol kinase
12	8080511	CACNA1D	776	2,05	1,97	2,44	2,7	1,74	2,02	2,74	2,84	2	calcium channel, voltage-dependent, L type, alpha 1D subunit
13	8045795	KCNJ3	3760	2	2,89	3,42	3,19	2,88	2,72	3,3	2,99	2,54	potassium inwardly-rectifying channel, subfamily J, member 3
C	8099476	PROM1	8842	2	2,79	3,02	2,29	3,31	3,12	2,72	2,18	21,67	prominin 1
14	7971731	ATP7B	540	1,98	3,42			2,60				3,64	ATPase, Cu++ transporting, beta polypeptide
15	7961900	ITPR2	3709	1,98	2,2		2,17	2,37	2,29	2,4			inositol 1,4,5-triphosphate receptor, type 2
16	8103951	ACSL1	2180	1,96	4,19	3,31	2,43	2,64	1,81			2,57	acyl-CoA synthetase long-chain family member 1
17	7961455	GUCY2C	2984	1,96	2,04	2,96		2,78	2,98			27,82	guanylate cyclase 2C (heat stable enterotoxin receptor)
18	8057959	PGAP1	80055	1,94	9,86	4,84	3,99	9,18	4,4	3,81	3,87	6,75	post-GPI attachment to proteins 1
19	8167305	EBP	10682	1,94	3,92	3,2	2,28	3,23	3,16	3,3	2,25	3,02	emopamil binding protein (sterol isomerase)
20	7950990	SLC36A4	120103	1,93	3,84	2,4		3,99	3,31	4,46	4,6	2,77	solute carrier family 36 (proton/amino acid symporter), member 4
21	8123739	NRN1	51299	1,88	2,78	2,78	7,63	1,99	3,32	5,31	7,7	3,27	neuritin 1
22	7947744	LRP4	4038	1,86	1,86			1,93	1,29	4,26	4,79	5,71	low density lipoprotein receptor-related protein 4
23	8123246	SLC22A3	6581	1,84	11,93	12,95	14,25	3,08	2,15	4,85	6,17	4,51	solute carrier family 22 (extraneuronal monoamine transporter), member 3
24	7906904	HSD17B7	51478	1,82	3,12	4,66	4,24	2,58	3,81	4,53	3,77	3,1	hydroxysteroid (17-beta) dehydrogenase 7
25	8056151	PLA2R1	22925	1,82	2,3	2,35		2,22	1,82			2,2	phospholipase A2 receptor 1, 180kDa
26	7961279	TAS2R14	50840	1,8	2,31	2,4	2,63	2,45	2,15	2,27	2,41	2,14	taste receptor, type 2, member 14
27	8175492	ATP11C	286410	1,8	2			1,82	1,77				ATPase, class VI, type 11C
28	8175666	GABRE	2564	1,78	2,8	2,93	2,91	1,97	1,47	3,56	3,04	3,26	gamma-aminobutyric acid (GABA) A receptor, epsilon
29	8078014	SLC6A6	6533	1,77	4,58	3,49	3,3	2,27	2,37	4,87	5,7		solute carrier family 6 (neurotransmitter transporter, taurine), member 6
30	8178977	TAPBP	6892	1,76			2,19				2,4		TAP binding protein (tapasin)
31	7897774	CLCN6	1185	1,76	2,01			2,28	1,93				chloride channel 6
32	7950067	DHCR7	1717	1,73	7,8	5,22	4,29	4,40	4,28			3,17	7-dehydrocholesterol reductase
33	7953218	RAD51AP1	10635	1,72	6,46	2,83	2,07	2,88	1,58				RAD51 associated protein 1
34	8123006	SYNJ2	8871	1,71	3,68	7,27	3,84	3,26	3,15	4,58	4,42	3,64	synaptojanin 2
35	8152355	SYBU	55638	1,71	3,51			2,24				3,57	syntabulin (syntaxin-interacting)
36	8110265	FGFR4	2264	1,7	8,93	11,99	5,76	5,88	7	4,01	5,32	8,15	fibroblast growth factor receptor 4
37	8117140	CDKAL1	54901	1,69	2,75	4,37	3,76	2,16	2,99	3,16	3,58		CDK5 regulatory subunit associated protein 1-like 1
38	8147548	POP1	10940	1,69	3,93	2,92		2,31	1,77				processing of precursor 1, ribonuclease P/MRP subunit (S. cerevisiae)
39	8089372	KIAA1524	57650	1,69	2,36							-1,53	KIAA1524
40	8141708	CLDN15	24146	1,68	3,07	4,59	3,94	3,30	2,73	3,08	2,18	2,12	claudin 15
41	7981798	TUBGCP5	114791	1,68	3,73	3,38		3,42	2,73	2,62			tubulin, gamma complex associated protein 5
42	8141107	SLC25A13	10165	1,68	3,43	3,05	3,04	3,55	3,53	3,78	3,75	2,89	solute carrier family 25, member 13 (citrin)
43	7996772	SLC7A6	9057	1,68	2,51			1,79				1,63	solute carrier family 7 (cationic amino acid transporter, y+ system), member 6
44	7944656	SC5DL	6309	1,68	1,91			2,32	1,82			2,11	sterol-C5-desaturase (ERG3 delta-5-desaturase homolog, S. cerevisiae)-like
45	7997962	DPEP1	1800	1,67	23,05	30,47	34,52	3,52	6,17	24,86	6,39	10,18	dipeptidase 1 (renal)
46	7950307	UCP2	7351	1,67	1,95	5,43	7,12	1,87	2,78	6,81	6,71	2,82	uncoupling protein 2 (mitochondrial, proton carrier)
47	7901272	CYP4X1	260293	1,67	3,95	4,83	2,1	-1,30	-1,53		-7,17	2,48	cytochrome P450, family 4, subfamily X, polypeptide 1
48	8133818	PHTF2	57157	1,66	3,19	2,55	2,75	2,32					putative homeodomain transcription factor 2
49	7999387	EMP2	2013	1,66		2,26		2,00	2,4	4,79	4,3	2,78	epithelial membrane protein 2
50	8049349	UGT1A1	54658	1,65	4,88	12,39	15,12	17,76	23,9	79,87	76,86	8,73	UDP glucuronosyltransferase 1 family, polypeptide A1
51	7940565	FADS2	9415	1,65	6,92	8,15	10,98	5,97	6,46	7,12	11,39	5,05	fatty acid desaturase 2
52	7909628	FLVCR1	28982	1,64	7,54	4,41	3,18	4,38	4,24			2,59	feline leukemia virus subgroup C cellular receptor 1
53	7967736	POLE	5426	1,64	1,84			1,93	2,02				polymerase (DNA directed), epsilon
54	8148280	SQLE	6713	1,63	5,18	2,42	2,24	2,27	1,81			2,38	squalene epoxidase
55	8137526	INSIG1	3638	1,62	3,94	2,5	2,79	3,20	2,79			4,17	insulin induced gene 1
56	8056572	SPC25	57405	1,59	14,65	15,2	7,85	4,68	3,91	4,97	3,34	2,15	SPC25, NDC80 kinetochore complex component, homolog (S. cerevisiae)
57	8113491	STARD4	134429	1,58	3,8			4,17	2,85	2,3		3,11	StAR-related lipid transfer (START) domain containing 4
58	7972217	SPRY2	10253	1,57	2,19			2,28	2,4	2,59	2,56	4,39	sprouty homolog 2 (Drosophila)
59	7948612	FADS1	3992	1,56	11,11	11,44	18,5	6,11	5,85	2,94	2,84	4,78	fatty acid desaturase 1
60	8130720	SFT2D1	113402	1,56	2,69	3,05	2,55	2,78	2,1	2,93	2,41	1,87	SFT2 domain containing 1
61	7920472	TPM3	7170	1,56	2,79	2,64		2,54	1,89				tropomyosin 3
62	8088642	LRIG1	26018	1,56		2,5		1,97				4,37	leucine-rich repeats and immunoglobulin-like domains 1
63	7951717	TMPRSS5	80975	1,56	2,35		2,33	2,22	2,02			2,13	transmembrane protease, serine 5
64	8135661	CFTR	1080	1,56	1,78			2,10	2,27		-3,31	16,98	cystic fibrosis transmembrane conductance regulator (ATP-binding cassette sub-family C, member 7)
65	8045768	ARL6IP6	151188	1,55	3,08			2,65	1,88			2	ADP-ribosylation-like factor 6 interacting protein 6
66	8135341	CDHR3	222256	1,55	2,41	2,6		2,48	2,7	3,13	3,02	2,54	cadherin-related family member 3
67	8107769	SLC12A2	6558	1,54	3,07	2,77						2,66	solute carrier family 12 (sodium/potassium/chloride transporters), member 2
68	7950796	CREBZF	58487	1,54	2,67			2,09					CREB/ATF bZIP transcription factor
69	8080562	IL17RB	55540	1,54	1,75	2,48	2,03					1,93	interleukin 17 receptor B
70	8131143	LFNG	3955	1,53	2,82	3,31	4	2,71	2,89	3,64	3,8	2,62	LFNG O-fucosylpeptide 3-beta-N-acetylglucosaminyltransferase
71	7924603	LBR	3930	1,51	1,99			1,73				2,17	lamin B receptor
72	7913869	STMN1	3925	1,5	2,25								stathmin 1
73	7916282	LRP8	7804	1,49	7,24	8,15	8,66	3,03	3,13	4,8	4,26	2,1	low density lipoprotein receptor-related protein 8, apolipoprotein e receptor
74	7912112	DNAJC11	55735	1,48		3,03	3,59		1,8	2,77	3,46		DnaJ (Hsp40) homolog, subfamily C, member 11

75	7901993	CACHD1	57685	1,47	3,75		4,7	3,14	2,27		3,82	2,32	cache domain containing 1
C	7898809	EPHB2	2048	1,46	5,34	14,28	7,51	3,26	6,3	5,19	7,52	7,05	EPH receptor B2
76	7928695	FAM213A	84293	1,46		6,17	5,05		3,1	4,26	3,09	2,44	chromosome 10 open reading frame 58
77	8172425	SLC38A5	92745	1,46	2,94	3,5	3	1,30	2,02			1,78	solute carrier family 38, member 5
78	8066407	FITM2	128486	1,46	2,36	2,65	2,31	1,79	1,96				fat storage-inducing transmembrane protein 2
79	8155083	CA9	768	1,45	4,59	4,58	5,06	3,20	1,71	6,25	6,9	3,64	carbonic anhydrase IX
C	8082846	EPHB1	2047	1,45	3,83	4,6	4,81	2,94	3,52	4,94	5,16	2,92	EPH receptor B1
80	7949619	SLC29A2	3177	1,44	2,82	3,18	2,92	2,34	2,67	2,52	3,01	2,05	solute carrier family 29 (nucleoside transporters), member 2
81	8151223	SLCO5A1	81796	1,44	2,64	2,68		2,39	2,27		2,58	2,36	solute carrier organic anion transporter family, member 5A1
82	7982938	TYRO3	7301	1,42	2,32			1,87	2,1	2,42			TYRO3 protein tyrosine kinase
83	8108744	PCDHB14	56122	1,42	1,89				-2,59	-6,5	-6,35	1,51	protocadherin beta 14
84	7951703	DRD2	1813	1,38	3,88	4,48	4,46	3,88	3,56	4,7	5,55	4,71	dopamine receptor D2
85	8078196	KCNH8	131096	1,37	6,75	6,33	4,83	4,32	2,57	6,94	5,18	5,73	potassium voltage-gated channel, subfamily H (eag-related), member 8
86	8092241	KCNMB3	27094	1,37	1,85		2,52	1,81					potassium large conductance calcium-activated channel, subfamily M beta member 3
87	7972428	OXGR1	27199	1,35	6,95	9,78	8,95	4,09	4,47	8,43	11,23	6,38	oxoglutarate (alpha-ketoglutarate) receptor 1
88	8022692	DSC3	1825	1,34	1,65	1,89	1,95	-1,32		-7,42	-4,77	-1,03	desmocollin 3
89	8042310	SLC1A4	6509	1,34	2,55	4,2	4,42	2,20	2,03	3,33	3,95	2,02	solute carrier family 1 (glutamate/neutral amino acid transporter), member 4
90	8149592	SLC18A1	6570	1,31	5,52	6,75	4,04	6,21	6,44	9,78	7,76	8,78	solute carrier family 18 (vesicular monoamine), member 1
91	8131666	ITGB8	3696	1,31	2,56	2,49	2,33	2,12				1,93	integrin, beta 8
92	7997504	CDH13	1012	1,3			2,08					2,3	cadherin 13, H-cadherin (heart)
93	8072587	SLC5A1	6523	1,28	2,84			1,59				5,99	solute carrier family 5 (sodium/glucose cotransporter), member 1
94	8054451	EDAR	10913	1,27	2,32		2,23	1,54		2,4		1,76	ectodysplasin A receptor
95	8136557	TBXAS1	6916	1,26	2,9	3,62	5,2	1,77	1,76	2,36	3,5	7,91	thromboxane A synthase 1 (platelet)
96	8163383	SUSD1	64420	1,21	2,23			2,01				2,45	sushi domain containing 1
97	8150722	ST18	9705	1,19	10,27	8,79	4,57	11,98	8,51	11,15	12,43	12,34	suppression of tumorigenicity 18 (breast carcinoma) (zinc finger protein)
98	8127145	ELOVL5	60481	1,18	4,31	2,96	5,73	2,00	1,68			4,56	ELOVL family member 5, elongation of long chain fatty acids (FEN1/Elo2, SUR4/Elo3-like, yeast)
99	8045336	GPR39	2863	1,18		3,46	3,72	1,67		3,11	2,62		G protein-coupled receptor 39
100	8047174	SLC39A10	57181	1,15	2,85				-2,06	-4,36	-4,48		solute carrier family 39 (zinc transporter), member 10
101	7954330	SLCO1B3	28234	1,11	2	2,35	2,32	-2,46	-2,89	-10,49	-12,9	-5,76	solute carrier organic anion transporter family, member 1B3
102	8083494	MME	4311	1,02	1,73		3,42	1,32	1,4		2,52	1,49	membrane metallo-endopeptidase
103	8108693	PCDHB4	56131	1,01	2,37	2,55	2,87	2,48	2,49	2,47	2,51	2,39	protocadherin beta 4

56 CSC targets Score List

1st selection	103 candidates were selected as the top genes differentially upregulated in CSCs vs normal colon tissues, colon cancer tissues and cell lines; 47 were discarded for their probable intracellular localization; 56 were further examined.
CSC specificity	According to Affymetrix data, genes were scored as follows: (+) indicates a minimum 2-fold higher expression compared to at least two of three normal tissues datasets, at least two of five cancer tissue datasets and around 100 colon cancer cell lines.
Expression in normal adult tissues (RNA)	Two mRNA expression databases were examined; the Human Protein Atlas was also examined for protein expression, but turned out to be unreliable in most cases.
Selected by EXIRIS	18 out of 56 genes were identified with "YES" = accepted on the basis of a positive score, taking all the above criteria into consideration.<br>20 genes were identified with "NO" = excluded based on the same criteria.<br>18 genes were identified with "MAYBE" = intermediate evaluation, most of the time due to poor information on the biological function.<br>(B = biomarker) indicates genes significantly expressed in vital organs and therefore suitable as biomarkers, but not as therapeutic targets.

Gene	Description	CSC specificity	Expression in normal adult tissues (RNA level)		Biological function	Expression and role in cancer	Location (other than PM)	Expression and role in cancer	Location (other than PM)	Selected by EXIRIS
			BioGPS	Body Atlas (GEO GSE14938)						
1	8107100	RGMB	RGM domain family, member B	++	GI-tract, cerebellum	low in all	repulsive guidance molecule B (predicted protein) patterning of the developing nervous system, and maintenance of iron homeostasis; may act as a BMP coreceptor that potentiates BMP signaling (recombinant protein)	YES: negative regulator (KD exp. in prostate and breast cancer cell lines); expressed in breast cancer (RNA level): lower in breast cancer with local recurrence and distant metastasis, but relatively higher in the patients who died from the disease	?	YES (B)
4	8111490	PRLR	prolactin receptor	++	low in all	low in all	cell survival/proliferation/migration, PRL- and GH-dependent, through JAK/STAT and MAPK activation; CRC cell lines: PRL increases sphere formation and migration (Proceedings AACR Meeting 2012)	YES: ovarian and breast cancer cell lines; CRC primary+metastasis	soluble splice variant	YES
6	7937952	OR51E1	olfactory receptor, family 51, subfamily E, member 1	+++	prostate	low in all	putative GPCR (Dresden, D GPCR); 3-methyl-valeric acid and 4-methyl-valeric acid (odorants) identified as agonists using a cAMP/CRE-reporter assay on a recombinant rhodopsin-tagged receptor	YES (RNA level): prostate cancer; lung and colon cancers (unpublished); small intestin carcinoids (neuroendocrine midgut tumors). Candidate tumorigenic genetic/somatic mutations in CRC and breast cancer (<5%), and maybe in pancreatic cancer (J. Hopkins)	?	YES
7	8059525	TM4SF20	transmembrane 4 L six family member 20	+	small intestine	low in all	patent applications (WO/2010/073694; EP 2 385 114 A1) claim: mAb by genetic immunization that shows ADCC and CDCC on A549 cells and IHC PM positivity on gastric tumor samples	YES: (Patent applications WO/2010/073694; EP2385114A1) mRNA expression in gastric, colon, pancreatic and lung cancers	?	YES
8	8162059	SLC28A3	solute carrier family 28 (sodium-coupled nucleoside transporter), member 3	++	low in all	low in all	sodium-dependent, pyrimidine- and purine-selective transporter; gemcitabine, AZT, ribavirin and 3-deazauridine transporter; up-regulated by PMA in HL-60 cells	YES in CLL: linked to poor prognosis, fludarabine resistance; frequently expressed in ovarian carcinoma effusions	ER splice variant	YES
9	8169836	XPNPEP2	X-prolyl aminopeptidase (aminopeptidase P) 2, membrane-bound	+	kidney and small intestine (also at protein level and literature)	kidney	metalloprotease specific for N-terminal imido bonds present in collagen degradation products, neuro-and vasoactive peptides, cytokines. May play a role in the inflammatory process, in the response to injury or infection and in the metabolism of the vasodilator bradykinin; member of the 'pita bread fold' family; specifically expressed in breast vasculature	YES (RNA level, patent WO2004104575A2): esophagous and gastric tumors, breast, ovary, kidney cancers	soluble form (in brain and leukocytes, in one case from a separate gene)	YES (B)
10	8104570	FAM105A	family with sequence similarity 105, member A	+	low in all	CD4+ and CD8+ T-cells, bone marrow, lung, spleen	(transcript level); homologous to Dbl-homology guanine nucleotide exchange factors (DH-GEFs) that regulate actin cytoskeletal reorganization, cell adhesion, and gene transcription via activation of Rho GTPases	?	?	MAYBE

**12**	8080511	**CACNA1D**	calcium channel, voltage-dependent, L type, alpha 1D subunit	**+**	**low in all**	**low in all**	**voltage-sensitive alpha-1 subunit of long-lasting (L-type) calcium channel. Prolactin increases trans-cellular intestinal calcium absorption by enhancing apical calcium uptake through voltage-dependent L-type calcium channel. CACNA1D expression may confer resistance to several drugs, including deoxydoxorubicin**	**YES (RNA level): breast cancer, prostate cancer, lymphoma samples; ERG target gene, overexpressed in TMPRSS2-ERG-positive prostate cancers**	**-**	**MAYBE**
**13**	8045795	**KCNJ3**	potassium inwardly-rectifying channel, subfamily J, member 3	**++**	**heart, brain**	**cerebellum**	**potassium channel controlled by G-proteins (Girk1); Growth induction of breast cancer cells in synergy with the β-adrenergic receptor pathway and of SCLC cells in antagonism with the β-adrenergic receptor pathway**	**YES: breast cancer and NSCLC tissue specimens, expression associated with a more aggressive clinical behavior and lymph node metastasis, respectively. Expressed also in some SCLC cell lines**	**?**	**YES**

1st selectio	Probe	Gene	Description	CSC specificity	Expression in normal adult tissues (RNA level)		Biological function	Expression and role in cancer	Location (other than PM)	Selected by EXIRIS
					BioGPS	Body Atlas (GEO GSE14938)				
15	7961900	ITPR2	inositol 1,4,5-triphosphate receptor, type 2	±	CD33* myeloid and CD56+ NK cells	low in all	downregulated by hypoxia in 293 cells, upregulated in the oxidative stress response in 293 cells. Cell survival. EGFR, FGFR, NGFR signaling pathways. Cell response to cAMP	?	also in ER	NO
17	7961455	GUCY2C	guanylate cyclase 2C (heat stable enterotoxin receptor)	+	low in all; literature: intestine	low in all	receptor for the paracrine hormones guanylin and uroguanylin; cell growth inhibitor, genomic stability maintenance	YES: overexpressed in intestinal cancer (biomarker assays in place), but signaling is inactivated by ligand silencing (funtional tumor suppressor)	-	YES
20	7950990	SLC36A4	solute carrier family 36 (proton/amino acid symporter), member 4	++	low in all	low in all	SLC36A4 /hPAT4 member of SLC36 family of amino acid transporters based on sequence similarity	?	?	MAYBE
21	8123739	NRN1	neuritin 1	++	brain, dorsal root ganglion	brain	growth-promoting protein known to mediate neurite outgrowth; hypoxia-induced mRNA in human microendothelial cells (HMEC-1); KD inhibits Kaposi sarcoma virus transformation of dermal microendothelial cells; ectopic expression in NIH 3T3 cells results in morphological changes, anchorage-independent growth and tumor formation in mice	YES: overexpression in astrocytoma, positively correlated with malignancy as reflected by changes in proliferation, apoptosis, and angiogenesis markers	-	MAYBE (B)
22	7947744	LRP4	low density lipoprotein receptor-related protein 4	+	brain	weak brain, skleletal muscle, spinal cord and skin	negatively regulates Wnt signaling; also binds to the Wise BMP antagonist integrating Wnt and BMB pathways during teeth development	?	?	MAYBE (B)
23	8123246	SLC22A3	solute carrier family 22 (extraneuronal monoamine transporter), member 3	++	prostate	salivary gland, skleletal muscle, prostate	polyspecific organic cation transporter; increases chemosensitivity	downregulated in aggressive prostate and liver cancers; increases chemosensitivity in CR and renal cancer cells	-	MAYBE(B)
25	8056151	PLA2R1	phospholipase A2 receptor 1, 180kDa	+	low in all; literature: kidney, pancreas	thyroid, kidney	receptor for secretory phospholipase A2 (sPLA2); activates MAPK cascade to induce cell proliferation; stimulates extracellular matrix invasion by normal and cancer cells; involved in inducible nitric oxide synthase expression via Akt	sPLA2 overexpressed and oncogenic in prostate cancer; receptor expression unknown	soluble form (shedded)	MAYBE (B)
26	7961279	TAS2R14	taste receptor, type 2, member 14	++	low in all	low in all	member of the taste receptor family, GPCR superfamily; detoxification, cell protection in the GI	?	-	MAYBE
27	8175492	ATP11C	ATPase, class VI, type 11C	±	low in all	low in all	probable phospholipid-transporting ATPase thought to serve as flippase concentrating aminophospholipids in the cytoplasmic leaflet of cell membranes; phosphorylated upon DNA damage, probably by ATM/ATR; mutated mice develop hepatocellular carcinoma and disruption of B cell development and antibody production	?	also in ER	NO
28	8175666	GABRE	gamma-aminobutyric acid (GABA) A receptor, epsilon	+	placenta; literature: brain, testis	placenta, adipose	epsilon subunit of the (GABA)A receptor, a ligand-gated Cl^- channel; its activation promotes tumor cell line proliferation and induces osmotic swelling, cell cycle activation and EGFR expression of neonatal CD133+ neural precursors; (GABA)A receptor subtypes containing the ε subunit exhibit spontaneous, agonist-independent, channel activity	YES (GABAA-R): pancreatic and prostate cancer	-	YES

1st selectio	Probe	Gene	Description	CSC specificity	Expression in normal adult tissues (RNA level)		Biological function	Expression and role in cancer	Location (other than PM)	Selected by EXIRIS
					BioGPS	Body Atlas (GEO GSE14938)				
29	8078014	SLC6A6	solute carrier family 6 (neurotransmitter transporter, taurine), member 6	+	low in all; protein level: colon	retina, trachea, adrenal gland, spleen, bone marrow	(/TauT) Na-/Cl-dependent, high-affinity, low-capacity transporter of taurine; high taurine uptake associates with high cell proliferation and cell survival; TNF-alpha stimulates taurine uptake and SLC6A6 expression in Caco-2 cells and taurine represses the damage to Caco-2 cells caused by TNF-alpha; taurine may be a protective substance against intestinal inflammation; expression of SLC6A6 decreases after activation of p53 by doxorubicin in 293 and NRK-52E renal cells; functionally expressed in sphere-forming neural precursor cells; induced by oncogenes, stress, hypertonicity	?	?	YES (B)
31	7897774	CLCN6	chloride channel 6	±	brain	retina, brain	NO: intracellular, endosomal ONLY	?	ONLY ENDOSOMAL	NO
36	8110265	FGFR4	fibroblast growth factor receptor 4	+++	liver, lung, kidney, pancreas	liver, weak kidney, ovary, lung	mitogenic and angiogenic activity; expression induced by HNF1	YES: breast, ovarian, prostate cancer	soluble splice variant	MAYBE
39	8089372	KIAA1524	KIAA1524	±; non CSC but also tumor specific	low in all	CD4+ and CD8+ Tcells	encodes CIP2A oncoprotein that inhibits PP2A and stabilizes MYC; promotes anchorage-independent cell growth	YES: tumor promoter and poor prognosis biomarker in several solid tumors; anti-CIP2A Abs in patient sera	?	YES
40	8141708	CLDN15	claudin 15	++	intestin, liver, CD71+ erythroid cells, T lymphocytes	low all	member of the claudin family, components of tight junction strands that also play critical roles in maintaining cell polarity and signal transductions; claudin-15 upregulated in colitis-associated cancer in animal models	claudin-1 and -2 are overexpressed in CRC; claudin-1 expression has prognostic value in colon cancer, claudin-18 in gastric cancer, and claudin-10 in hepatocellular carcinoma; claudin 15 unknown	?	YES
43	7996772	SLC7A6	solute carrier family 7 (cationic amino acid transporter, y+ system), member 6	±; non CSC but also tumor specific	lymphocytes and dendritic cells, CD34+ cells, adypocytes	thymus, lymphnode, adipose	uptake of essential amino acids; activates the mTORC1 signaling that requires aa such as leucine for its function in prostate cancer cell lines	YES: expressed in prostate cancer and different carcinoma cell lines; SNPs associated with etoposide sensitivity	-	NO
45	7997962	DPEP1	dipeptidase 1 (renal)	+++	kidney and pancreas; protein level: GI-tract	kidney	hydrolyzes a variety of dipeptides and is implicated in renal metabolism of glutathione and its conjugates	YES: highly expressed in colorectal adenoma and cancer specimens; negatively correlated with parameters of pathological aggressiveness and poor prognosis. Expressed in the early stages of colon carcinogenesis and negatively affects cancer cell invasiveness	-	YES (B)
49	7999387	EMP2	epithelial membrane protein 2	+	small intestine, colon, liver, lung, prostate, skin and other tissues; undiff. embryonic stem cells	lung, placenta, adipose, skin, cervix, heart, ileum, jejenum, liver, mammary gland	cell proliferation and survival; angiogenesis via HIF1alpha/VEGF induction	YES: tumor promoter, poor prognosis biomarker and diabodies/mAb target in breast, ovary, endometrial cancers	-	MAYBE (B)
52	7909628	FLVCR1	feline leukemia virus subgroup C cellular receptor 1	++	low in all; literature: hematopoietic tissues, PBMCs (RNA level)	CD4+ and CD8+ Tcells, bone marrow	heme transporter that exports cytoplasmic heme, coproporphyrin and protoporphyrin IX, both intermediate products in the heme biosynthetic pathway; does not export bilirubin; may provide protection from heme or ferrous iron toxicities in developing erythroid cells, liver and brain	YES: CACO2 and HepG2 cell lines	-	MAYBE
62	8088642	LRIG1	leucine-rich repeats and immunoglobulin-like domains 1	±	brain, GI-tract, T lymphocytes; literature: undiff. embryonic stem cells and normal quiescent colon SC (Lgr5-) and skin SC	mammary gland, placenta, brain, GI-tract, adipose, heart, spinal cord, skeletal muscle, prostate	binds & suppresses EGFR and MET by inducing their ubiq./degr. and RET by blunting GDNF-med. activation; repression of proliferation and response to oxidative damage signature in colon quiescent SC; regulator of stem cell quiescence in skin	tumor suppressor in intestinal cancer	soluble ectodomain	YES
63	7951717	TMPRSS5	transmembrane protease, serine 5	+	low in all; literature: CNS	low in all	(/Spinesin) belongs to the transmembrane serine protease family and is a potential single-pass type II membrane protein; may play a role in hearing	YES (RNA level): stomach, ileum, liver, uterus tumors (Patent Application WO/2004/098487A2)	?	NO
66	8135341	CDHR3	cadherin-related family member 3	++	low in all	trachea, lung, jejunum, ileum, colon, epididymis	predicted protein: cadherin-related	?	?	NO

1st selecti	Probe	Gene	Description	CSC specificity	Expression in normal adult tissues (RNA level)		Biological function	Expression and role in cancer	Location (other than PM)	Selected by EXIRIS
					BioGPS	Body Atlas (GEO GSE14938)				
67	8107769	SLC12A2	solute carrier family 12 (sodium/potassium/chloride transporters), member 2	+; non CSC but also tumor specific	colon, trachea	trachea, duodenum, jejunum, ileum, skin, spinal cord	phosphorylated upon DNA damage (probably by ATM/ATR), induced by Wnt in CRC cells	YES: overexpressed in CRC (protein level); in patent WO2006110593 is included in a series of biological targets for diagnosis, treatment and prevention of cancer	-	MAYBE (B)
69	8080562	IL17RB	interleukin 17 receptor B	+; non CSC but also tumor specific	kidney, brain areas; protein level: kidney, liver, testis, adrenal, parathyroids	kidney	binds the proinflammatory cytokines IL17B and IL17E; upregulated in intestinal inflammation; expression on macrophages induced by IL4 and enhanced by TGFβ	Low levels, good prognosis in breast cancer	soluble isoform	MAYBE
73	7916282	LRP8	low density lipoprotein receptor-related protein 8, apolipoprotein e receptor	++	testis, pineal gland	CD8+ T-cells, Retina	high-affinity binding of APOE-containing lipoproteins, such as reelin; positive factor of canonical Wnt pathway in osteoprogenitor cells	YES: gene amplification and overexpression in squamous cell lung carcinoma	soluble form (shedded)	YES
75	7901993	CACHD1	cache domain containing 1	+	low in all	ovary, thalamus	(transcript level); potential single-pass type I membrane protein; belongs to the calcium channel subunit alpha-2/delta family and may regulate voltage-dependent Ca channels (by similarity)	downregulated in different solid cancers (Nextbio database)	?	NO
77	8172425	SLC38A5	solute carrier family 38, member 5	+; non CSC but also tumor specific	erythroid precursors, endothelial cells, pancreas, bronchus	CD4+ and CD8+ Tcells, bone marrow, salivary gland	Na-dependent neutral amino acid transporter which countertransport protons; mediates glutamine absorption in crypt cells; mediates resistance to drugs; downregulated, together with claudin5 (other target) in Lrp5 KO mice retina with defects in blood vessel development (Wnt target gene, angiogenesis)	?	?	YES (B)
79	8155083	CA9	carbonic anhydrase IX	++	low in all; protein level: GI-tract, bile ducts, gall bladder	stomach	cell proliferation, survival, transformation, invasion, hypoxia resistance	YES, pharmacological tumor target	-	NO
80	7949619	SLC29A2	solute carrier family 29 (nucleoside transporters), member 2	+	skeletal muscle and heart	low in all	(/ENT2) mediates equilibrative transport of purine, pyrimidine nucleosides and hypoxanthine; mediates hypersensitization to nucleoside analogs (eg. fludarabine) in CLL; induced by PDGF and FGF; repression by HIF-1alpha or pharmacological inhibition attenuates hypoxia-associated inflammation of the intestine	?	-	NO
81	8151223	SLCO5A1	solute carrier organic anion transporter family, member 5A1	+	low in all	low in all	chemoresistance to satraplatin	YES: breast cancer, satraplatin-resistant SCLC cell lines	also in the cytoplasm	NO
82	7982938	TYRO3	TYRO3 protein tyrosine kinase	±	brain, testis	ovary	belongs to the TAM family (Tyro3, Axl, and Mer) of tyrosine kinase receptors activated by different ligands (GAS6, TULP1, protein S); controls cell proliferation, survival, migration, differentiation; activates AKT survival pathway by interaction with PIK3R1 and enhancement of PI3-kinase activity; activates ERK1/ERK2 by unknown mechanisms	YES: AML, CML, multiple myeloma, and melanoma, endometrial cancer; coexpressed with AXL and GAS6 in CXCR4-positive tyroid cancer cells and in thyroid tumors; therapeutic target for bladder cancer (WO 2010/031828)	soluble form (shedded)	NO
83	8108744	PCDHB14	protocadherin beta 14	±; non CSC but also tumor specific	female reproductive apparatus, skin, CNS (downregulated in hematopoietic tissues, blood cells and GI tract)	low in all	(transcript level); potential calcium-dependent cell-adhesion protein	?	?	NO

1st selectio	Probe	Gene	Description	CSC specificity	Expression in normal adult tissues (RNA level)		Biological function	Expression and role in cancer	Location (other than PM)	Selected by EXIRIS
					BioGPS	Body Atlas (GEO GSE14938)				
84	7951703	DRD2	dopamine receptor D2	++	moderate in brain, pituitary, smooth muscles	pituitary gland	D2 subtype of the dopamine receptor; by binding to selective agonists, transactivates, via c-Src, EGFR that activates the PI 3-kinase/Akt-mediated cell survival response (oxidative stress-induced); dopamine exerts a protective effect for stomach and intestine against experimental carcinogenesis in animal models; ectopically expressed DRD2 inhibits canonical Wnt signalling by interacting with beta-catenin in the cytoplasm and inhibiting its entry into the nucleus	decreased dopamine content in CRC; SNPs within DRD2 associated with CRC, related to reduced levels of D2 dopamine receptor	-	YES
85	8078196	KCNH8	potassium voltage-gated channel, subfamily H (eag-related), member 8	++	low in all	low in all	pore-forming (alpha) subunit of voltage-gated potassium channel	YES: gene amplification and correlation with aggressiveness poor prognosis in prostate cancer	?	NO
86	8092241	KCNMB3	potassium large conductance calcium-activated channel, subfamily M beta member 3	±; non CSC but also tumor specific	low in all	low in all	regulatory beta subunit of the Ca-activated potassium KCNMA1 (maxiK) channel; channel inactivating function (3 out of 4 isoforms)	downregulated in thyroid cancer and upregulated in liver cancer, lymphomas and leukemias	?	NO
87	7972428	OXGR1	oxoglutarate (alpha-ketoglutarate) receptor 1	+++	low in all	low in all	receptor for alpha-ketoglutarate that is a substrate for dioxygenases (histone demethylases, prolyl hydroxylases, collagen prolyl-4-hydroxylases, TET family of 5-methlycytosine hydroxylases); belongs to the GPCR 1 family	downregulated in some tumors (liver, kidney), upregulated in others (intestin, ovary) (NextBio database)	?	YES
88	8022692	DSC3	desmocollin 3	±; non CSC but also tumor specific	skin and bronchial epithelium (also at protein level)	skin	adhesive protein of the desmosome cell-cell junction	downregulated by DNA hypermethylation in CRC	-	MAYBE
89	8042310	SLC1A4	solute carrier family 1 (glutamate/neutral amino acid transporter), member 4	++	brain, dendritic, CD33+ myeloid cells	brain, CD4+ Tcells, mammary gland, skeletal muscle, thymus	transporter for small neutral amino acids	?	?	MAYBE (B)
91	8131666	ITGB8	integrin, beta 8	±	low in all; literature: brain, kidney, ovary, uterus	brain areas, spinal cord	αvβ8 is a receptor for fibronectin and the latent domain LAP of TGF-β; binding to αvβ8 with subsequent LAP cleavage induces TGF-β activation in vivo; cell proliferation induction	YES: angiogenesis and cell invasiveness in GBM	-	NO
92	7997504	CDH13	cadherin 13, H-cadherin (heart)	±; non CSC but also tumor specific	low in all; protein level: muscles, heart, kidney	heart, brain, cervix, lung, mammary gland, skeletal muscle, skin, adipose	member of the cadherin superfamily	downregulated by DNA hypermethylation in many types of cancer; gene silencing associated with tumor aggressiveness	-	NO
93	8072587	SLC5A1	solute carrier family 5 (sodium/glucose cotransporter), member 1	±; non CSC but also tumor specific	small intestine (also at protein level)	mammary gland, heart, small intestine	member of the Na-dependent glucose transporter (SGLT) family and primary mediator of dietary glucose and galactose uptake from the intestinal lumen; upregulated by wt and oncogenic recombinant JAK2 (in X. laevis oocytes) and downregulated by JAK2 inhibitor AG490; may contribute to glucose uptake into oncogenic JAK2 expressing tumor cells	YES: tumor cell lines	also intracellular	MAYBE (B)
94	8054451	EDAR	ectodysplasin A receptor	+; non CSC but also tumor specific	pancreas	low in all	member of TNF receptor family; soluble ligand is ectodysplasin A (EDA isoform A1); required for the development of hair, teeth, and other ectodermal derivatives; activates NF-kappaB; interdependence of EDA/EDAR/NF-kappaB and Wnt pathways in initiation and maintenance of primary hair follicle placodes; putative death receptor: controversial reports; increased expression induced by ectopic expression of p53 and in p53-dependent response to genotoxic stresses caused by adriamycinin (glioblastoma cell line)	?	-	MAYBE

1st selectio	Probe	Gene	Description	CSC specificity	Expression in normal adult tissues (RNA level)		Biological function	Expression and role in cancer	Location (other than PM)	Selected by EXIRIS
					BioGPS	Body Atlas (GEO GSE14938)				
99	8045336	GPR39	G protein-coupled receptor 39	±	low in all	low in all	increases cell proliferation, tumor formation, G1/S transition via up-regulation of cyclin D1 and CDK6; enhances cell motility and invasiveness by inducing EMT and remodeling cytoskeleton	YES: esophageal squamous cell carcinomas (associated with lymph node metastasis and advanced TNM stage)	-	NO
100	8047174	SLC39A10	solute carrier family 39 (zinc transporter), member 10	±; non CSC but also tumor specific	brain, dendritic and CD34+ cells, T lymphocytes	low all	(/ZIP10) belongs to a subfamily of proteins that show structural characteristics of zinc-influx transporters	YES: upregulated in invasive and metastatic breast cancer and cell lines	?	NO
101	7954330	SLCO1B3	solute carrier organic anion transporter family, member 1B3	+; non CSC but also tumor specific	low in all; protein level: liver	kidney	Na-independent uptake of organic anions [17-beta-glucuronosyl estradiol, taurocholate, triiodothyronine (T3), leukotriene C4, dehydroepiandrosterone sulfate (DHEAS), methotrexate and sulfobromophthalein (BSP)]; involved in the clearance of bile acids and organic anions from the liver. Ectopic expression in a mammalian tumor cell line enhances sensitivity to methotrexate	YES: liver, gall bladder, colon, pancreas, breast, lung and prostate cancers; potential prognostic factor in breast and colon cancer patients; association between genetic variations and clinical outcomes in prostate cancer patients	-	NO
102	8083494	MME	membrane metallo-endopeptidase	±; non CSC but also tumor specific	whole blood, 34+ cells, adypose, small intestine, prostate, smooth muscles; protein level: GI, spleen, kidney, lung, breast, prostate	kidney, prostate, adipose, liver, lung placenta	MME gene encodes neprilysin (NEP/CALLA/Enkephalinase/ Neutral endopeptidase-NEP/Skin fibroblast elastase-SFE/ CD10) that cleaves peptides of up to 30 aa at the N-side of hydrophobic residues (particularly Phe or Tyr); activating phosphorylation of the N-term cytoplasmic domain abolishes binding to PTEN and derepresses insulin/IGF-1 stimulated activation of Akt	YES: ALL cell surface diagnostic marker; expression in tumor stroma enhances tumorigenicity of CD133+ colon cancer cells and breast and pancreatic CSCs	-	MAYBE (B)
103	8108693	PCDHB4	protocadherin beta 4	+	hyppocampus, spinal cord, breast, adipose, parotides	CD4+ and CD8+ T-cells	(transcript level); potential calcium-dependent cell-adhesion protein	?	?	NO

36 CSC targets Score List

1st selection	103 candidates (predicted transmembrane or GPI-anchored) were selected as the top genes differentially upregulated in CSCs vs normal colon tissues, colon cancer tissues and cell lines
CSC specificity	According to Affymetrix data, genes were scored as follows: (+) indicates an at least 2-fold higher expression with respect to at least two out of three normal tissues datasets AND with respect to at least two out of five cancer tissue datasets AND with respect to about 100 colon cancer cell lines.
Expression in normal adult tissues (RNA level)	Two mRNA expression databases were examined; the Human Protein Atlas was also examined for protein expression, but turned out to be unreliable in most cases.
Selected by EXI	18 out of 56 genes were identified with "YES" = accepted on the basis of a positive score, taking all the above criteria into consideration.<br>20 genes were identified with "NO" = excluded based on the same criteria.<br>18 genes were identified with "MAYBE" = intermediate evaluation, most of the time due to poor information on the biological function.<br>(B = biomarker) indicates genes significantly expressed in vital organs and therefore suitable as biomarkers, but not as therapeutic targets.

1st selection	Probe	Gene	Description	CSC specificity	Expression in normal adult tissues (RNA level)		Biological function	Expression and role in cancer	Location (other than PM)	Selected by EXIRIS
					BioGPS	Body Atlas (GEO GSE14938)				
1	8107100	RGMB	RGM domain family, member B	++	GI-tract, cerebellum	low in all	repulsive guidance molecule B (predicted protein) patterning of the developing nervous system, and maintenance of iron homeostasis; may act as a BMP coreceptor that potentiates BMP signaling (recombinant protein)	YES: negative regulator (KD exp. in prostate and breast cancer cell lines); expressed in breast cancer (RNA level): lower in breast cancer with local recurrence and distant metastasis, but relatively higher in patients who died from the disease	?	YES (B)
4	8111490	PRLR	prolactin receptor	++	low in all	low in all	cell survival/proliferation/migration, PRL- and GH-dependent, through JAK/STAT and MAPK activation; CRC cell lines: PRL increases sphere formation and migration (Proceedings AACR Meeting 2012)	YES: ovarian and breast cancer cell lines; CRC primary+metastasis	soluble splice variant	YES
6	7937952	OR51E1	olfactory receptor, family 51, subfamily E, member 1	+++	prostate	low in all	putative GPCR (Dresden, D GPCR); 3-methyl-valeric acid and 4-methyl-valeric acid (odorants) identified as agonists using a cAMP/CRE-reporter assay on a recombinant rhodopsin-tagged receptor	YES (RNA level): prostate cancer; lung and colon cancers (unpublished); small intestin carcinoids (neuroendocrine midgut tumors). Candidate tumorigenic genetic/somatic mutations in CRC and breast cancer (<5%), and maybe in pancreatic cancer (J. Hopkins)	?	YES
7	8059525	TM4SF20	transmembrane 4 L six family member 20	+	small intestine	low in all	patent applications (WO/2010/073694; EP 2 385 114 A1) claim: mAb by genetic immunization that shows ADCC and CDCC on A549 cells and IHC PM positivity on gastric tumor samples	YES: (Patent applications WO/2010/073694; EP2385114A1) mRNA expression in gastric, colon, pancreatic and lung cancers	?	YES
8	8162059	SLC28A3	solute carrier family 28 (sodium-coupled nucleoside transporter), member 3	++	low in all	low in all	sodium-dependent, pyrimidine- and purine-selective transporter; gemcitabine, AZT, ribavirin and 3-deazauridine transporter; up-regulated by PMA in HL-60 cells	YES in CLL: linked to poor prognosis, fludarabine resistance; frequently expressed in ovarian carcinoma effusions	ER splice variant	YES
9	8169836	XPNPEP2	X-prolyl aminopeptidase (aminopeptidase P) 2, membrane-bound	+	kidney and small intestine (also at protein level and literature)	kidney	metalloprotease specific for N-terminal imido bonds present in collagen degradation products, neuro-and vasoactive peptides, cytokines. May play a role in the inflammatory process, in the response to injury or infection and in the metabolism of the vasodilator bradykinin; member of the 'pita bread fold' family; specifically espressed in breast vasculature	YES (RNA level, patent WO2004104575A2): esophagous and gastric tumors, breast, ovary, kidney cancers	soluble form (in brain and leukocytes, in one case from a separate gene)	YES (B)
10	8104570	FAM105A	family with sequence similarity 105, member A	+	low in all	CD4+ and CD8+ T-cells, bone marrow, lung, spleen	(transcript level); homologous to Dbl-homology guanine nucleotide exchange factors (DH-GEFs) that regulate actin cytoskeletal reorganization, cell adhesion, and gene transcription via activation of Rho GTPases	?	?	MAYBE
12	8080511	CACNA1D	calcium channel, voltage-dependent, L type, alpha 1D subunit	+	low in all	low in all	voltage-sensitive alpha-1 subunit of long-lasting (L-type) calcium channel. Prolactin increases trans-cellular intestinal calcium absorption by enhancing apical calcium uptake through voltage-dependent L-type calcium channel. CACNA1D expression may confer resistance to several drugs, including deoxydoxorubicin.	YES (RNA level): breast cancer, prostate cancer, lymphoma samples; ERG target gene, overexpressed in TMPRSS2-ERG-positive prostate cancers	-	MAYBE
13	8045795	KCNJ3	potassium inwardly-rectifying channel, subfamily J, member 3	++	heart, brain	cerebellum	potassium channel controlled by G-proteins (Girk1); Growth induction of breast cancer cells in synergy with the β-adrenergic receptor pathway and of SCLC cells in antagonism with the β-adrenergic receptor pathway	YES: breast cancer and NSCLC tissue specimens, expression associated with a more aggressive clinical behavior and lymph node metastasis, respectively. Expressed also in some SCLC cell lines	?	YES

1st selection	Probe	Gene	Description	CSC specificity	Expression in normal adult tissues (RNA level)		Biological function	Expression and role in cancer	Location (other than PM)	Selected by EXIRIS
					BioGPS	Body Atlas (GEO GSE14938)				
17	7961455	GUCY2C	guanylate cyclase 2C (heat stable enterotoxin receptor)	+	low in all; literature: intestine	low in all	receptor for the paracrine hormones guanylin and uroguanylin; cell growth inhibitor, genomic stability maintenance	YES: overexpressed in intestinal cancer (biomarker assays in place), but signaling is inactivated by ligand silencing (funtional tumor suppressor)	-	YES
20	7950990	SLC36A4	solute carrier family 36 (proton/amino acid symporter), member 4	++	low in all	low in all	SLC36A4 /hPAT4 member of SLC36 family of amino acid transporters based on sequence similarity	?	?	MAYBE
21	8123739	NRN1	neuritin 1	++	brain, dorsal root ganglion	brain	growth-promoting protein known to mediate neurite outgrowth; hypoxia-induced mRNA in human microendothelial cells (HMEC-1); KD inhibits Kaposi sarcoma virus transformation of dermal microendothelial cells; ectopic expression in NIH 3T3 cells results in morphological changes, anchorage-independent growth and tumor formation in mice	YES: overexpression in astrocytoma, positively correlated with malignancy as reflected by changes in proliferation, apoptosis, and angiogenesis markers	-	MAYBE (B)
22	7947744	LRP4	low density lipoprotein receptor-related protein 4	+	brain	weak brain, skleletal muscle, spinal cord and skin	negatively regulates Wnt signaling; also binds to the Wise BMP antagonist integrating Wnt and BMB pathways during teeth development	?	?	MAYBE (B)
23	8123246	SLC22A3	solute carrier family 22 (extraneuronal monoamine transporter), member 3	++	prostate	salivary gland, skleletal muscle, prostate	polyspecific organic cation transporter; increases chemosensitivity	downregulated in aggressive prostate and liver cancers; increases chemosensitivity in CR and renal cancer cells	-	MAYBE (B)
25	8056151	PLA2R1	phospholipase A2 receptor 1, 180kDa	+	low in all; literature: kidney, pancreas	thyroid, kidney	receptor for secretory phospholipase A2 (sPLA2); activates MAPK cascade to induce cell proliferation; stimulates extracellular matrix invasion by normal and cancer cells; involved in inducible nitric oxide synthase expression via Akt	sPLA2 overexpressed and oncogenic in prostate cancer; receptor expression unknown	soluble form (shedded)	MAYBE (B)
26	7961279	TAS2R14	taste receptor, type 2, member 14	++	low in all	low in all	member of the taste receptor family, GPCR superfamily; detoxification, cell protection in the GI	?	-	MAYBE
28	8175666	GABRE	gamma-aminobutyric acid (GABA) A receptor, epsilon	+	placenta; literature: brain, testis	placenta, adipose	epsilon subunit of the (GABA)A receptor, a ligand-gated Cl^- channel; its activation promotes tumor cell line proliferation and induces osmotic swelling, cell cycle activation and EGFR expression of neonatal CD133+ neural precursors; (GABA)A receptor subtypes containing the ε subunit exhibit spontaneous, agonist-independent, channel activity	YES (GABAA-R): pancreatic and prostate cancer	-	YES
29	8078014	SLC6A6	solute carrier family 6 (neurotransmitter transporter, taurine), member 6	+	low in all; protein level: colon	retina, trachea, adrenal gland, spleen, bone marrow	(/TauT) Na-/Cl-dependent, high-affinity, low-capacity transporter of taurine; high taurine uptake associates with high cell proliferation and cell survival; TNF-alpha stimulates taurine uptake and SLC6A6 expression in Caco-2 cells and taurine represses the damage to Caco-2 cells caused by TNF-alpha; taurine may be a protective substance against intestinal inflammation; expression of SLC6A6 decreases after activation of p53 by doxorubicin in 293 and NRK-52E renal cells; functionally expressed in sphere-forming neural precursor cells; induced by oncogenes, stress, hypertonicity	?	?	YES (B)
36	8110265	FGFR4	fibroblast growth factor receptor 4	+++	liver, lung, kidney, pancreas	liver, weak kidney, ovary, lung	mitogenic and angiogenic activity; expression induced by HNF1	YES: breast, ovarian, prostate cancer	soluble splice variant	MAYBE

1st selection	Probe	Gene	Description	CSC specificity	Expression in normal adult tissues (RNA level)		Biological function	Expression and role in cancer	Location (other than PM)	Selected by EXIRIS
					BioGPS	Body Atlas (GEO GSE14938)				
39	8089372	KIAA1524	Protein CIP2A	±; non CSC but also tumor specific	low in all	CD4+ and CD8+ Tcells	encodes CIP2A oncoprotein that inhibits PP2A and stabilizes MYC; promotes anchorage-independent cell growth	YES: tumor promoter and poor prognosis biomarker in several solid tumors; anti-CIP2A Abs in patient sera	?	YES
40	8141708	CLDN15	claudin 15	++	intestin, liver, CD71+ erythroid cells, T lymphocytes	low all	member of the claudin family, components of tight junction strands that also play critical roles in maintaining cell polarity and signal transductions; claudin-15 upregulated in colitis-associated cancer in animal models	claudin-1 and -2 are overexpressed in CRC; claudin-1 expression has prognostic value in colon cancer, claudin-18 in gastric cancer, and claudin-10 in hepatocellular carcinoma; claudin 15 unknown	?	YES
45	7997962	DPEP1	dipeptidase 1 (renal)	+++	kidney and pancreas; protein level: GI-tract	kidney	hydrolyzes a variety of dipeptides and is implicated in renal metabolism of glutathione and its conjugates	YES: highly expressed in colorectal adenoma and cancer specimens; negatively correlated with parameters of pathological aggressiveness and poor prognosis. Expressed in the early stages of colon carcinogenesis and negatively affects cancer cell invasiveness	-	YES (B)
49	7999387	EMP2	epithelial membrane protein 2	+	small intestine, colon, liver, lung, prostate, skin and other tissues; undiff. embryonic stem cells	lung, placenta, adipose, skin, cervix, heart, ileum, jejenum, liver, mammary gland	cell proliferation and survival; angiogenesis via HIF1alpha/VEGF induction	YES: tumor promoter, poor prognosis biomarker and diabodies/mAb target in breast, ovary, endometrial cancers	-	MAYBE (B)
52	7909628	FLVCR1	feline leukemia virus subgroup C cellular receptor 1	++	low in all; literature: hematopoietic tissues, PBMCs (RNA level)	CD4+ and CD8+ Tcells, bone marrow	heme transporter that exports cytoplasmic heme, coproporphyrin and protoporphyrin IX, both intermediate products in the heme biosynthetic pathway; does not export bilirubin; may provide protection from heme or ferrous iron toxicities in developing erythroid cells, liver and brain	YES: CACO2 and HepG2 cell lines	-	MAYBE
62	8088642	LRIG1	leucine-rich repeats and immunoglobulin-like domains 1	±	brain, GI-tract, T lymphocytes; literature: undiff. embryonic stem cells and normal quiescent colon SC (Lgr5-) and skin SC	mammary gland, placenta, brain, GI-tract, adipose, heart, spinal cord, skeletal muscle, prostate	binds & suppresses EGFR and MET by inducing their ubiq./degr. and RET by blunting GDNF-med. activation; repression of proliferation and response to oxidative damage signature in colon quiescent SC; regulator of stem cell quiescence in skin	tumor suppressor in intestinal cancer	soluble ectodomain	YES
67	8107769	SLC12A2	solute carrier family 12 (sodium/potassium/chloride transporters), member 2	+; non CSC but also tumor specific	colon, trachea	trachea, duodenum, jejunum, ileum, skin, spinal cord	phosphorylated upon DNA damage (probably by ATM/ATR), induced by Wnt in CRC cells	YES: overexpressed in CRC (protein level); in patent WO2006110593 is included in a series of biological targets for diagnosis, treatment and prevention of cancer	-	MAYBE (B)
69	8080562	IL17RB	interleukin 17 receptor B	+; non CSC but also tumor specific	kidney, brain areas; protein level: kidney, liver, testis, adrenal, parathyroids	kidney	binds the proinflammatory cytokines IL17B and IL17E; upregulated in intestinal inflammation; expression on macrophages induced by IL4 and enhanced by TGFβ	low levels, good prognosis in breast cancer	soluble isoform	MAYBE
73	7916282	LRP8	low density lipoprotein receptor-related protein 8, apolipoprotein e receptor	++	testis, pineal gland	CD8+ T-cells, Retina	high-affinity binding of APOE-containing lipoproteins, such as reelin; positive factor of canonical Wnt pathway in osteoprogenitor cells	YES: gene amplification and overexpression in squamous cell lung carcinoma	soluble form (shedded)	YES
77	8172425	SLC38A5	solute carrier family 38, member 5	+; non CSC but also tumor specific	erythroid precursors, endothelial cells, pancreas, bronchus	CD4+ and CD8+ Tcells, bone marrow, salivary gland	Na-dependent neutral amino acid transporter which countertransport protons; mediates glutamine absorption in crypt cells; mediates resistance to drugs; downregulated, together with claudin5 (other target) in Lrp5 KO mice retina with defects in blood vessel development (Wnt target gene, angiogenesis)	?	?	YES (B)
84	7951703	DRD2	dopamine receptor D2	++	moderate in brain, pituitary, smooth muscles	pituitary gland	D2 subtype of the dopamine receptor; by binding to selective agonists, transactivates, via c-Src, EGFR that activates the PI 3-kinase/Akt-mediated cell survival response (oxidative stress-induced); dopamine exerts a protective effect for stomach and intestine against experimental carcinogenesis in animal models; ectopically expressed DRD2 inhibits canonical Wnt signalling by interacting with beta-catenin in the cytoplasm and inhibiting its entry into the nucleus	decreased dopamine content in CRC; SNPs within DRD2 associated with CRC, related to reduced levels of D2 dopamine receptor	-	YES
87	7972428	OXGR1	oxoglutarate (alpha-ketoglutarate) receptor 1	+++	low in all	low in all	receptor for alpha-ketoglutarate that is a substrate for dioxygenases (histone demethylases, prolyl hydroxylases, collagen prolyl-4-hydroxylases, TET family of 5-methlycytosine hydroxylases); belongs to the GPCR 1 family	downregulated in some tumors (liver, kidney), upregulated in others (intestin, ovary) (NextBio database)	?	YES

1st selection	Probe	Gene	Description	CSC specificity	Expression in normal adult tissues (RNA level)		Biological function	Expression and role in cancer	Location (other than PM)	Selected by EXIRIS
					BioGPS	Body Atlas (GEO GSE14938)				
88	8022692	DSC3	desmocollin 3	±; non CSC but also tumor specific	skin and bronchial epithelium (also at protein level)	skin	adhesive protein of the desmosome cell-cell junction	downregulated by DNA hypermethylation in CRC	-	MAYBE
89	8042310	SLC1A4	solute carrier family 1 (glutamate/neutral amino acid transporter), member 4	++	brain, dendritic, CD33+ myeloid cells	brain, CD4+ Tcells, mammary gland, skeletal muscle, thymus	transporter for small neutral amino acids	?	?	MAYBE (B)
93	8072587	SLC5A1	solute carrier family 5 (sodium/glucose cotransporter), member 1	±; non CSC but also tumor specific	small intestine (also at protein level)	mammary gland, heart, small intestine	member of the Na-dependent glucose transporter (SGLT) family and primary mediator of dietary glucose and galactose uptake from the intestinal lumen; upregulated by wt and oncogenic recombinant JAK2 (in X. laevis oocytes) and downregulated by JAK2 inhibitor AG490; may contribute to glucose uptake into oncogenic JAK2 expressing tumor cells	YES: tumor cell lines	also intracellular	MAYBE (B)
94	8054451	EDAR	ectodysplasin A receptor	+; non CSC but also tumor specific	pancreas	low in all	member of TNF receptor family; soluble ligand is ectodysplasin A (EDA isoform A1); required for the development of hair, teeth, and other ectodermal derivatives; activates NF-kappaB; interdependence of EDA/EDAR/NF-kappaB and Wnt pathways in initiation and maintenance of primary hair follicle placodes; putative death receptor: controversial reports; increased expression induced by ectopic expression of p53 and in p53-dependent response to genotoxic stresses caused by adriamycinin (glioblastoma cell line)	?	-	MAYBE
102	8083494	MME	membrane metallo-endopeptidase	±; non CSC but also tumor specific	whole blood, 34+ cells, adypose, small intestine, prostate, smooth muscles; protein level: GI, spleen, kidney, lung, breast, prostate	kidney, prostate, adipose, liver, lung placenta	MME gene encodes neprilysin (NEP/CALLA/Enkephalinase/ Neutral endopeptidase-NEP/Skin fibroblast elastase-SFE/ CD10) that cleaves peptides of up to 30 aa at the N-side of hydrophobic residues (particularly Phe or Tyr); activating phosphorylation of the N-term cytoplasmic domain abolishes binding to PTEN and derepresses insulin/IGF-1 stimulated activation of Akt	YES: ALL cell surface diagnostic marker; expression in tumor stroma enhances tumorigenicity of CD133+ colon cancer cells and breast and pancreatic CSCs	-	MAYBE (B)

36 genes relative expression List

FC vs Differentiated	Global average ratio (fold change, FC) between CSCs and differentiated
FC vs Normal Colon<br>FC vs Normal Colon Microdissected<br>FC vs Normal Colon Microdissected	Global average FC between CSCs and bulk normal colon, or microdissected normal colon tissue
FC vs Tumor (Subset 1)<br>FC vs Tumor (Subset 2)<br>FC vs Tumor Microdissected 1<br>FC vs Tumor Microdissected 2<br>FC vs Tumor Microdissected 3	Global average FC between CSCs and bulk colon tumor tissue (subset 1 or 2), or microdissected colon tumor tissue
FC vs Colon Cancer Cell lines	Global average FC between CSCs and colon cancer cell lines
In all cases a blank white space indicates that the global average FC was not considered statistically signficant	

					NORMAL			TUMOR					CELLS
1st selection	HuGene probe	NCBI Gene	Description	FC vs Differentiated	FC vs Normal Colon	FC vs Normal Colon Microdissected 1	FC vs Normal Colon Microdissected 2	FC vs Tumor (Subset 1)	FC vs Tumor (Subset 2)	FC vs Tumor Microdissected 1	FC vs Tumor Microdissected 2	FC vs Tumor Microdissected 3	FC vs Colon Cancer Cell lines
1	8107100	RGMB	RGM domain family, member B	3	4,26	4,25		5,29	5,87	3,98	4,16	3,16	8,71
4	8111490	PRLR	prolactin receptor	2,6	2,2	2,74	2,71	2,11	2,13	2,23	2,17	2,53	2,62
6	7937952	OR51E1	olfactory receptor, family 51, subfamily E, member 1	2,5	77,47	68,04	59,68	59,5	55,95	25,34	13,14	9,91	92,08
7	8059525	TM4SF20	transmembrane 4 L six family member 20	2,4	2,61		2,49	2,16	2,38	2,38	-4,06	-3,94	6,7
8	8162059	SLC28A3	solute carrier family 28 (sodium-coupled nucleoside transporter), member 3	2,4	3,81	3,52	2,68	2,98	3,12	2,14	4,48	3,83	4,11
9	8169836	XPNPEP2	X-prolyl aminopeptidase (aminopeptidase P) 2, membrane-bound	2,4		2,22	2,36	1,69	1,7	2,45	2,27	2,69	3,02
10	8104570	FAM105A	family with sequence similarity 105, member A	2,3	2,63			3,21	3,24	2,39	2,26		10,92
12	8080511	CACNA1D	calcium channel, voltage-dependent, L type, alpha 1D subunit	2,1	1,97	2,44	2,7	1,8	1,67	2,02	2,74	2,84	2
13	8045795	KCNJ3	potassium inwardly-rectifying channel, subfamily J, member 3	2	2,89	3,42	3,19	2,92	2,83	2,72	3,3	2,99	2,54
17	7961455	GUCY2C	guanylate cyclase 2C (heat stable enterotoxin receptor)	2	2,04	2,96		2,51	3,05	2,98			27,82
20	7950990	SLC36A4	solute carrier family 36 (proton/amino acid symporter), member 4	1,9	3,84	2,4		3,99	3,98	3,31	4,46	4,6	2,77
21	8123739	NRN1	neuritin 1	1,9	2,78	2,78	7,63	2,12	1,85	3,32	5,31	7,7	3,27
22	7947744	LRP4	low density lipoprotein receptor-related protein 4	1,9	1,86			1,82	2,03	1,29	4,26	4,79	5,71
23	8123246	SLC22A3	solute carrier family 22 (extraneuronal monoamine transporter), member 3	1,8	11,93	12,95	14,25	3,19	2,96	2,15	4,85	6,17	4,51
25	8056151	PLA2R1	phospholipase A2 receptor 1, 180kDa	1,8	2,3	2,35		2,21	2,22	1,82			2,2
26	7961279	TAS2R14	taste receptor, type 2, member 14	1,8	2,31	2,4	2,63	2,47	2,42	2,15	2,27	2,41	2,14
28	8175666	GABRE	gamma-aminobutyric acid (GABA) A receptor, epsilon	1,8	2,8	2,93	2,91	1,97	1,96	1,47	3,56	3,04	3,26
29	8078014	SLC6A6	solute carrier family 6 (neurotransmitter transporter, taurine), member 6	1,8	4,58	3,49	3,3	2,33	2,21	2,37	4,87	5,7	
36	8110265	FGFR4	fibroblast growth factor receptor 4	1,7	8,93	11,99	5,76	5,73	6,02	7	4,01	5,32	8,15
39	8089372	KIAA1524	Protein CIP2A	1,7	2,36								-1,53
40	8141708	CLDN15	claudin 15	1,7	3,07	4,59	3,94	3,36	3,23	2,73	3,08	2,18	2,12
45	7997962	DPEP1	dipeptidase 1 (renal)	1,7	23,05	30,47	34,52	3,43	3,6	6,17	24,86	6,39	10,18
49	7999387	EMP2	epithelial membrane protein 2	1,7		2,26		1,99	2,01	2,4	4,79	4,3	2,78
52	7909628	FLVCR1	feline leukemia virus subgroup C cellular receptor 1	1,6	7,54	4,41	3,18	4,53	4,22	4,24			2,59
62	8088642	LRIG1	leucine-rich repeats and immunoglobulin-like domains 1	1,6		2,5		1,94	2				4,37
67	8107769	SLC12A2	solute carrier family 12 (sodium/potassium/chloride transporters), member 2	1,5	3,07	2,77							2,66
69	8080562	IL17RB	interleukin 17 receptor B	1,5	1,75	2,48	2,03						1,93
73	7916282	LRP8	low density lipoprotein receptor-related protein 8, apolipoprotein e receptor	1,5	7,24	8,15	8,66	2,97	3,09	3,13	4,8	4,26	2,1
77	8172425	SLC38A5	solute carrier family 38, member 5	1,5	2,94	3,5	3	1,25	1,35	2,02			1,78
84	7951703	DRD2	dopamine receptor D2	1,4	3,88	4,48	4,46	3,79	3,97	3,56	4,7	5,55	4,71
87	7972428	OXGR1	oxoglutarate (alpha-ketoglutarate) receptor 1	1,4	6,95	9,78	8,95	3,87	4,3	4,47	8,43	11,23	6,38
88	8022692	DSC3	desmocollin 3	1,3	1,65	1,89	1,95		-1,32		-7,42	-4,77	-1,03
89	8042310	SLC1A4	solute carrier family 1 (glutamate/neutral amino acid transporter), member 4	1,3	2,55	4,2	4,42	2,22	2,18	2,03	3,33	3,95	2,02
93	8072587	SLC5A1	solute carrier family 5 (sodium/glucose cotransporter), member 1	1,3	2,84			1,54	1,63				5,99
94	8054451	EDAR	ectodysplasin A receptor	1,3	2,32		2,23	1,55	1,53		2,4		1,76
102	8083494	MME	membrane metallo-endopeptidase	1	1,73		3,42		1,32	1,4		2,52	1,49

Final 21 Targets List

1st selection	103 candidates (predicted transmembrane or GPI-anchored) were selected as the top genes differentially upregulated in CSCs vs normal colon tissues, colon cancer tissues and cell lines;<br>47 were discarded for their probable intracellular localization;<br>56 were further examined.
CSC specificity	According to Affymetrix data, the 56 genes were scored as follows: (+) indicates an at least 2-fold higher expression with respect to at least two out of three normal tissues datasets AND with respect to at least two out of five cancer tissue datasets AND with respect to about 100 colon cancer cell lines.
Expression in normal adult tissues (RNA level)	Two mRNA expression databases were examined; the Human Protein Atlas was also examined for protein expression, but turned out to be unreliable in most cases.
Selected by EXI	18 out of 56 genes were identified with "YES" = accepted on the basis of a positive score, taking all the above criteria into consideration.<br>20 genes were identified with "NO" = excluded based on the same criteria.<br>18 genes were identified with "MAYBE" = intermediate evaluation, most of the time due to poor information on the biological function.<br>(B = biomarker) indicates genes significantly expressed in vital organs and therefore suitable as biomarkers, but not as therapeutic targets.
Final 21 selected	21 out of 36 genes ("YES" + "MAYBE") were selected after discussion with Aldevron for TAQMAN analysis: 13 genes "YES" and 8 genes "MAYBE"

1st selection	Probe	Gene	Description	CSC specificity	Expression in normal adult tissues (RNA level)		Biological function	Expression and role in cancer	Location (other than PM)	Final 21 selected
					BioGPS	Body Atlas (GEO GSE14938)				
1	8107100	RGMB	RGM domain family, member B	++	GI-tract, cerebellum	low in all	repulsive guidance molecule B (predicted protein) patterning of the developing nervous system, and maintenance of iron homeostasis; may act as a BMP coreceptor that potentiates BMP signaling (recombinant protein)	YES: negative regulator (KD exp. in prostate and breast cancer cell lines); expressed in breast cancer (RNA level): lower in breast cancer with local recurrence and distant metastasis, but relatively higher in patients who died from the disease	?	YES (B)
4	8111490	PRLR	prolactin receptor	++	low in all	low in all	cell survival/proliferation/migration, PRL- and GH-dependent, through JAK/STAT and MAPK activation; CRC cell lines: PRL increases sphere formation and migration (Proceedings AACR Meeting 2012)	YES: ovarian and breast cancer cell lines; CRC primary+metastasis	soluble splice variant	YES
6	7937952	OR51E1	olfactory receptor, family 51, subfamily E, member 1	+++	prostate	low in all	putative GPCR (Dresden, D GPCR); 3-methyl-valeric acid and 4-methyl-valeric acid (odorants) identified as agonists using a cAMP/CRE-reporter assay on a recombinant rhodopsin-tagged receptor	YES (RNA level): prostate cancer; lung and colon cancers (unpublished); small intestin carcinoids (neuroendocrine midgut tumors). Candidate tumorigenic genetic/somatic mutations in CRC and breast cancer (<5%), and maybe in pancreatic cancer (J. Hopkins)	?	YES
7	8059525	TM4SF20	transmembrane 4 L six family member 20	+	small intestine	low in all	patent applications (WO/2010/073694; EP 2 385 114 A1) claim: mAb by genetic immunization that shows ADCC and CDCC on A549 cells and IHC PM positivity on gastric tumor samples	YES: (Patent applications WO/2010/073694; EP2385114A1) mRNA expression in gastric, colon, pancreatic and lung cancers	?	YES
9	8169836	XPNPEP2	X-prolyl aminopeptidase (aminopeptidase P) 2, membrane-bound	+	kidney and small intestine (also at protein level and literature)	kidney	metalloprotease specific for N-terminal imido bonds present in collagen degradation products, neuro-and vasoactive peptides, cytokines. May play a role in the inflammatory process, in the response to injury or infection and in the metabolism of the vasodilator bradykinin; member of the 'pita bread fold' family; specifically espressed in breast vasculature	YES (RNA level, patent WO2004104575A2): esophagous and gastric tumors, breast, ovary, kidney cancers	soluble form (in brain and leukocytes, in one case from a separate gene)	YES (B)
17	7961455	GUCY2C	guanylate cyclase 2C (heat stable enterotoxin receptor)	+	low in all; literature: intestine	low in all	receptor for the paracrine hormones guanylin and uroguanylin; cell growth inhibitor, genomic stability maintenance	YES: overexpressed in intestinal cancer (biomarker assays in place), but signaling is inactivated by ligand silencing (funtional tumor suppressor)	-	YES
22	7947744	LRP4	low density lipoprotein receptor-related protein 4	+	brain	weak brain, skleletal muscle, spinal cord and skin	negatively regulates Wnt signaling; also binds to the Wise BMP antagonist integrating Wnt and BMB pathways during teeth development	?	?	MAYBE (B)
25	8056151	PLA2R1	phospholipase A2 receptor 1, 180kDa	+	low in all; literature: kidney, pancreas	thyroid, kidney	receptor for secretory phospholipase A2 (sPLA2); activates MAPK cascade to induce cell proliferation; stimulates extracellular matrix invasion by normal and cancer cells; involved in inducible nitric oxide synthase expression via Akt	sPLA2 overexpressed and oncogenic in prostate cancer; receptor expression unknown	soluble form (shedded)	MAYBE (B)
28	8175666	GABRE	gamma-aminobutyric acid (GABA) A receptor, epsilon	+	placenta; literature: brain, testis	placenta, adipose	epsilon subunit of the (GABA)A receptor, a ligand-gated Cl^- channel; its activation promotes tumor cell line proliferation and induces osmotic swelling, cell cycle activation and EGFR expression of neonatal CD133+ neural precursors; (GABA)A receptor subtypes containing the ε subunit exhibit spontaneous, agonist-independent, channel activity	YES (GABAA-R): pancreatic and prostate cancer	-	YES

1st selection	Probe	Gene	Description	CSC specificity	Expression in normal adult tissues (RNA level)		Biological function	Expression and role in cancer	Location (other than PM)	Final 21 selected
					BioGPS	Body Atlas (GEO GSE14938)				
36	8110265	FGFR4	fibroblast growth factor receptor 4	+++	liver, lung, kidney, pancreas	liver, weak kidney, ovary, lung	mitogenic and angiogenic activity; expression induced by HNF1	YES: breast, ovarian, prostate cancer	soluble splice variant	MAYBE
39	8089372	KIAA1524	Protein CIP2A	±; non CSC but also tumor specific	low in all	CD4+ and CD8+ Tcells	encodes CIP2A oncoprotein that inhibits PP2A and stabilizes MYC; promotes anchorage-independent cell growth	YES: tumor promoter and poor prognosis biomarker in several solid tumors; anti-CIP2A Abs in patient sera	?	YES
40	8141708	CLDN15	claudin 15	++	intestin, liver, CD71+ erythroid cells, T lymphocytes	low all	member of the claudin family, components of tight junction strands that also play critical roles in maintaining cell polarity and signal transductions; claudin-15 upregulated in colitis-associated cancer in animal models	claudin-1 and -2 are overexpressed in CRC; claudin-1 expression has prognostic value in colon cancer, claudin-18 in gastric cancer, and claudin-10 in hepatocellular carcinoma; claudin 15 unknown	?	YES
45	7997962	DPEP1	dipeptidase 1 (renal)	+++	kidney and pancreas; protein level: GI-tract	kidney	hydrolyzes a variety of dipeptides and is implicated in renal metabolism of glutathione and its conjugates	YES: highly expressed in colorectal adenoma and cancer specimens; negatively correlated with parameters of pathological aggressiveness and poor prognosis. Expressed in the early stages of colon carcinogenesis and negatively affects cancer cell invasiveness	-	YES (B)
49	7999387	EMP2	epithelial membrane protein 2	+	small intestine, colon, liver, lung, prostate, skin and other tissues; undiff. embryonic stem cells	lung, placenta, adipose, skin, cervix, heart, ileum, jejenum, liver, mammary gland	cell proliferation and survival; angiogenesis via HIF1alpha/VEGF induction	YES: tumor promoter, poor prognosis biomarker and diabodies/mAb target in breast, ovary, endometrial cancers	-	MAYBE (B)
62	8088642	LRIG1	leucine-rich repeats and immunoglobulin-like domains 1	±	brain, GI-tract, T lymphocytes; literature: undiff. embryonic stem cells and normal quiescent colon SC (Lgr5-) and skin SC	mammary gland, placenta, brain, GI-tract, adipose, heart, spinal cord, skeletal muscle, prostate	binds & suppresses EGFR and MET by inducing their ubiq./degr. and RET by blunting GDNF-med. activation; repression of proliferation and response to oxidative damage signature in colon quiescent SC; regulator of stem cell quiescence in skin	tumor suppressor in intestinal cancer	soluble ectodomain	YES
69	8080562	IL17RB	interleukin 17 receptor B	+; non CSC but also tumor specific	kidney, brain areas; protein level: kidney, liver, testis, adrenal, parathyroids	kidney	binds the proinflammatory cytokines IL17B and IL17E; upregulated in intestinal inflammation; expression on macrophages induced by IL4 and enhanced by TGFβ.	low levels, good prognosis in breast cancer	soluble isoform	MAYBE
73	7916282	LRP8	low density lipoprotein receptor-related protein 8, apolipoprotein e receptor	++	testis, pineal gland	CD8+ T-cells, Retina	high-affinity binding of APOE-containing lipoproteins, such as reelin; positive factor of canonical Wnt pathway in osteoprogenitor cells	YES: gene amplification and overexpression in squamous cell lung carcinoma	soluble form (shedded)	YES
87	7972428	OXGR1	oxoglutarate (alpha-ketoglutarate) receptor 1	+++	low in all	low in all	receptor for alpha-ketoglutarate that is a substrate for dioxygenases (histone demethylases, prolyl hydroxylases, collagen prolyl-4-hydroxylases, TET family of 5-methlycytosine hydroxylases); belongs to the GPCR 1 family	downregulated in some tumors (liver, kidney), upregulated in others (intestin, ovary) (NextBio database)	?	YES
88	8022692	DSC3	desmocollin 3	±; non CSC but also tumor specific	skin and bronchial epithelium (also at protein level)	skin	adhesive protein of the desmosome cell-cell junction	downregulated by DNA hypermethylation in CRC	-	MAYBE
94	8054451	EDAR	ectodysplasin A receptor	+; non CSC but also tumor specific	pancreas	low in all	member of TNF receptor family; soluble ligand is ectodysplasin A (EDA isoform A1); required for the development of hair, teeth, and other ectodermal derivatives; activates NF-kappaB; interdependence of EDA/EDAR/NF-kappaB and Wnt pathways in initiation and maintenance of primary hair follicle placodes; putative death receptor: controversial reports; increased expression induced by ectopic expression of p53 and in p53-dependent response to genotoxic stresses caused by adriamycinin (glioblastoma cell line)	?	-	MAYBE
102	8083494	MME	membrane metallo-endopeptidase	±; non CSC but also tumor specific	whole blood, 34+ cells, adypose, small intestine, prostate, smooth muscles; protein level: GI, spleen, kidney, lung, breast, prostate	kidney, prostate, adipose, liver, lung placenta	MME gene encodes neprilysin (NEP/CALLA/Enkephalinase/ Neutral endopeptidase-NEP/Skin fibroblast elastase-SFE/ CD10) that cleaves peptides of up to 30 aa at the N-side of hydrophobic residues (particularly Phe or Tyr); activating phosphorylation of the N-term cytoplasmic domain abolishes binding to PTEN and derepresses insulin/IGF-1 stimulated activation of Akt	YES: ALL cell surface diagnostic marker; expression in tumor stroma enhances tumorigenicity of CD133+ colon cancer cells and breast and pancreatic CSCs	-	MAYBE (B)
