# Supplementary material for: Targeting Colorectal Cancer Stem Cells Through Inhibition of the Fibroblast Growth Factor Receptor 4 Pathway with a Novel Antibody
Source: Cancers (Basel). 2026 Jan 28;18(3):418. doi: 10.3390/cancers18030418 (PMC12896886; doi:10.3390/cancers18030418)
Supplement: Supplementary file 1 [file cancers-18-00418-s001.zip › Table S2_cancers-4061516.pdf]

Table S2

List of RNAs from cell lines used for experimental validation of selected targets

CSC cell line	[RNA] (ug/mL)	A_{260} /A_{280}	28S/18S	RIN	Total RNA amount (ug)
1.1	274	1.9	1.0	8.5	19.6
1.2	406	1.3	1.4	8.1	20.3
18	960	1.8	1	7.8	48
85	374	1.85	1.2	8.5	18.7
CRO1	1250	2.1	1.2	8.9	89.3
CC1	247.5	1.7	1.6	8.3	17.7
CC2	644	1.7	1.3	9.1	32.2
CC5	700	1.8	1.73	9.5	50
**Colon tumor cell line**	**[RNA] (ug/mL)**	**A_{260} /A_{280}**	**28S/18S**	**RIN**	**Total RNA amount (ug)**
Caco-2	750	1.65	1.5	8.9	53.6
DLD-1	1612.5	1.9	2.2	10	115.2
HCT 116	2437.5	1.75	2.4	10	174
HCT 15	547.5	1.65	2	10	39.1
HT-29	1550	1.8	1.3	9.2	110.7
LoVo	2275	1.6	1.9	9.9	162.5
SNU-C2B	2500	2	1.7	9.8	178.6
SW48	2900	1.9	1.8	9.7	207
SW480	2425	2	1.8	10	173.2
SW620	4150	2	1.6	9.8	296.4
WiDr	1437.5	1.55	1.8	9.9	102.7
