# Supplementary material for: Targeting Colorectal Cancer Stem Cells Through Inhibition of the Fibroblast Growth Factor Receptor 4 Pathway with a Novel Antibody
Source: Cancers (Basel). 2026 Jan 28;18(3):418. doi: 10.3390/cancers18030418 (PMC12896886; doi:10.3390/cancers18030418)
Supplement: Supplementary file 1 [file cancers-18-00418-s001.zip › Table S3 Differential expression of the top 21 candidate genes in CSC vs other cells_cancers-4061516.pdf]

Table S3 Differential expression of the top 21 canditate genes in CSC vs other cells

Color code legend:

- **Targets with ≥ 3 independent positive CSC clones (accepted)= 9 genes**
- **Targets with 2 independent positive CSC clones (backup)= 4 genes**
- **Targets with 0 or 1 independent positive CSC clones (rejected)= 8 genes**
- *** CSC and tumor-specific according to Affymetrix data**
- **Positive score (CSC-specific)**
- **Positive score (CSC and tumor-specific)**
- **n. RNA: normal colon RNA**
- **colon CSC lines 1.1 and 1.2 were not considered independent, arising from the same patient**

		1			2			3			4			5	
		TM4SF20			OR51E1			PRLR			DPEP1			RGMB	
		%CSC expression			%CSC expression			%CSC expression			%CSC expression			%CSC expression	
	normalization	*GAPDH*	*HNRNPK*		*GAPDH*	*HNRNPK*		*GAPDH*	*HNRNPK*		*GAPDH*	*HNRNPK*		*GAPDH*	*HNRNPK*
**CSC 1.1**	DLD-1	< 0.0005432320	< 0.00078984	HCT15	< 0.0001036391	< 0.000107	HCT15	< 0.0002175	< 0.00022525				DLD-1	< 0.00019	< 0.0002890
	SNU-C2B	1,03	1,50	LoVo	0,0007	0,0012	SW48	0,15	0,078				HCT116	0,012	0,010
	SW620	0,26	0,24	SW480	0,0011	0,0014	SW480	< 0.0005585	< 0.00071184				LoVo	5,59	9,81
	WiDr	0,026	0,025	SW620	0,0023	0,0022	SW620	< 0.0007472	< 0.00071184				SW48	7,36	5,55
	n. RNA	4,07	2,88	n. RNA	1,22	0,86	n. RNA	18,05	12,76				SW620	0,0008	0,0007
													n. RNA	< 0.00015	< 0.0001080
**CSC 1.2**							HCT15	< 0.0001592	< 0.00011031	DLD-1	0,097	0,18			
							SW48	0,11	0,038	LoVo	0,0056	0,012			
							SW480	< 0.0004088	< 0.00034859	SW48	0,016	0,020			
							SW620	< 0.0005470	< 0.00034859	SW480	0,37	0,60			
							n. RNA	13,21	6,25	n. RNA	0,13	0,12			
**CSC 18**	DLD-1	< 0.16006903899	< 0.16972651	HCT15	< 0,0001603882	< 0,000134	HCT15	< 0.0457177	< 0.03451617				DLD-1	0,00222	0,0026201
	SNU-C2B	304	322	LoVo	0,001101631	0,001484	SW48	31,75	11,99				HCT116	0,13621	0,0911165
	SW620	75,41	52,39	SW480	0,00166976	0,001729	SW480	< 0.1173507	< 0.10907682				LoVo	62,8507	88,884268
	WiDr	7,66	5,47	SW620	0,003629172	0,002789	SW620	< 0.1570067	< 0.10907682				SW48	82,6636	50,335696
	n. RNA	1198	618	n. RNA	1,884074731	1,082117	n. RNA	3792	5372				SW620	0,00869	0,0067255
													n. RNA	0,0017	0,0009792
**CSC 85**	DLD-1	< 0.00025518942	< 0.00023810	HCT15	< 0.0010864640	< 0.000125	HCT15	< 0.0006505	< 0.00043216				DLD-1	< 0.00066	< 0.0006196
	SNU-C2B	0,48	0,45	LoVo	0,0075	0,0014	SW48	0,4518313	0,15				HCT116	0,041	0,022
	SW620	0,12	0,073	SW480	0,011	0,0016	SW480	< 0.0016697	< 0.00136569				LoVo	18,82	21,02
	WiDr	0,012	0,0077	SW620	0,025	0,002602	SW620	< 0.0022340	< 0.00136569				SW48	24,75	11,91
	n. RNA	1,91	0,87	n. RNA	12,76	1,01	n. RNA	53,96	24,49				SW620	0,0026	0,0016
													n. RNA	< 0.00051	< 0.0002315
**CSC CRO1**	DLD-1	< 0.0107954364	< 0.00928952	HCT15	< 0,0838442809	< 0,044620	HCT15	< 0.0010486	< 0.00064249	DLD-1	0,30	0,26	DLD-1	0,00031	0,0002348
	SNU-C2B	20,49	17,63	LoVo	0,575886413	0,491021	SW48	0,73	0,22	LoVo	0,02	0,02	HCT116	0,01929	0,0081661
	SW620	5,09	2,87	SW480	0,872880577	0,571908	SW480	< 0.0026916	< 0.00203039	SW48	0,05	0,03	LoVo	8,90031	7,9660039
	WiDr	0,52	0,30	SW620	1,897179507	0,922651	SW620	< 0.0036012	< 0.00203039	SW480	1,13	0,85	SW48	11,706	4,511196
	n. RNA	80,82	33,82	n. RNA	984,9155307	358,01	n. RNA	86,99	36,40	n. RNA	0,40	0,17	SW620	0,00123	0,0006028
													n. RNA	0,00024	8,776E-05
**CSC CC1**	DLD-1	< 0.51187242338	< 0.25066912				HCT15	< 0.0100536	< 0.00513242						
	SNU-C2B	971	476				SW48	6,98	1,78						
	SW620	241	77,38				SW480	< 0.0258061	< 0.01621930						
	WiDr	24,49	8,08				SW620	< 0.0345266	< 0.01621930						
	n. RNA	3832	913				n. RNA	834	291						
**CSC CC2**	DLD-1	undet.	undet.				HCT15	< 0.0100536	< 0.00513242	DLD-1	32,87	32,09	DLD-1	< 0.00161	< 0.0015579
	SNU-C2B	> 60887.4042881	> 43654.9064				SW48	6,98	1,78	LoVo	1,89	2,19	HCT116	0,10	0,054
	SW620	> 15116.7060662	> 7101.24462				SW480	< 0.0258061	< 0.01621930	SW48	5,57	3,56	LoVo	45,69	52,85
	WiDr	> 1534.82259092	> 741.270449				SW620	< 0.0345266	< 0.01621930	SW480	125	106	SW48	60,10	29,93
	n. RNA	> 240196.597602	> 83753.1707				n. RNA	834	291	n. RNA	44,60	21,02	SW620	0,00632	0,003999
													n. RNA	< 0.00123	< 0.0005822
**CSC CC5**	DLD-1	< 0.16197647379	< 0.12975447	HCT15	< 0.0011644433	< 0.000664	HCT15	< 0.0026751	< 0.00152587	DLD-1	2,50	2,01	DLD-1	< 0.00190	< 0.0015258
	SNU-C2B	307	246	LoVo	0,012	0,0073	SW48	1,86	0,53	LoVo	0,14	0,14	HCT116	0,12	0,053
	SW620	76,31	40,05	SW480	0,012	0,0085	SW480	< 0.0068668	< 0.00482202	SW48	0,42	0,22	LoVo	53,96	51,76
	WiDr	7,75	4,18	SW620	0,026	0,014	SW620	< 0.0091872	< 0.00482202	SW480	9,54	6,65	SW48	70,97	29,31
	n. RNA	1213	472	n. RNA	13,68	5,33	n. RNA	222	86,45	n. RNA	3,40	1,31	SW620	0,00746	0,0039
													n. RNA	< 0.00146	< 0.0005702

		6			7			8*			9			10	
		GABRE			XPNPEP2			DSC3			PLA2R1			GUCY2C	
		%CSC expression			%CSC expression			%CSC expression			%CSC expression			%CSC expression	
	normal.	*GAPDH*	*HNRNPK*		*GAPDH*	*HNRNPK*		*GAPDH*	*HNRNPK*		*GAPDH*	*HNRNPK*		*GAPDH*	*HNRNPK*
**CSC 1.1**				HCT116	< 0.00524	< 0.00431				Caco-2	1,86	1,14			
				HT-29	< 0.007672	< 0.00386				HCT116	1,37	1,13			
				SNU-C2B	< 0.002675	0,0039				SW480	2,38	3,04			
				SW620	1,24	1,18				SW620	0,77	0,73			
				n. RNA	114,9	125,7				n. RNA	65,07	46,01			
**CSC 1.2**	HCT116	5,91	6,12				HCT15	< 0.03381	< 0.044312				HCT15	< 0.0002268	< 0.0002972
	HCT15	4,67	6,08				SW48	0,14	0,12				SW48	< 0.0009072	< 0.0008120
	SNU-C2B	2,35	4,30				SW480	16,15	26,06				SW480	< 0.0005822	< 0.0009392
	SW480	1,30	2,09				SW620	4,64	5,59				WiDr	0,023	0,029
	n. RNA	263,9	236,2				n. RNA	2581,3	2310,3				n. RNA	70,71	63,29
**CSC 18**							HCT15	< 8.30428	< 7.279584				HCT15	< 0.0012723	< 0.0012071
							SW48	33,22	19,89				SW48	< 0.0050893	< 0.0032980
							SW480	3967	4281				SW480	< 0.0032658	< 0.0038147
							SW620	1139	919				WiDr	0,13	0,12
							n. RNA	633883	379530,47				n. RNA	397	257
**CSC 85**				HCT116	< 0.012637	< 0.00667				Caco-2	2,50	0,98	HCT15	< 0.0001232	< 0.0000813
				HT-29	< 0.018502	< 0.00597				HCT116	1,85	0,97	SW48	< 0.0004929	< 0.0002221
				SNU-C2B	< 0.006451	0,0060				SW480	3,21	2,61	SW480	< 0.0003163	< 0.0002569
				SW620	3,00	1,83				SW620	1,03	0,63	WiDr	0,013	0,0078
				n. RNA	277	84,09				n. RNA	87,66	39,50	n. RNA	38,42	17,31
**CSC CRO1**							HCT15	< 0.00440	< 0.001601				HCT15	< 0.0004608	< 0.0002823
							SW48	0,018	0,0044				SW48	< 0.0018432	< 0.0007713
							SW480	2,11	0,94				SW480	< 0.0011828	< 0.0008922
							SW620	0,60	0,20				WiDr	0,047	0,027
							n. RNA	336	83,51				n. RNA	144	60,12
**CSC CC1**	HCT116	15,07	5,26				HCT15	< 0.06137	< 0.027089						
	HCT15	11,91	5,22				SW48	0,25	0,074						
	SNU-C2B	6,00	3,69				SW480	29,32	15,93						
	SW480	3,30	1,79				SW620	8,42	3,42						
	n. RNA	673	203				n. RNA	4685	1412						
**CSC CC2**	HCT116	2,72	1,47	HCT116	undet.	undet.				Caco-2	3805	1562	HCT15	undet.	undet.
	HCT15	2,15	1,46	HT-29	undet.	undet.				HCT116	2805	1540	SW48	undet.	undet.
	SNU-C2B	1,08	1,03	SNU-C2B	undet.	> 62.6332				SW480	4884	4150	SW480	undet.	undet.
	SW480	0,60	0,50	SW620	> 30128.81	> 19067.8				SW620	1567	995	WiDr	> 2208.5077	> 1437.0117
	n. RNA	121	56,64	n. RNA	> 2784247	> 1307932				n. RNA	133257	62817	n. RNA	> 6761238.8	> 3176168.8
**CSC CC5**				HCT116	< 0.015775	< 0.00710									
				HT-29	< 0.023097	0,0064									
				SNU-C2B	< 0.008053	0,0064									
				SW620	3,74	1,95									
				n. RNA	346	134									

		11			12			13*			14			15*	
		LRP4			CLDN15			IL17RB			FGFR4			KIAA1524	
		%CSC expression			%CSC expression			%CSC expression			%CSC expression			%CSC expression	
	normal.	*GAPDH*	*HNRNPK*		*GAPDH*	*HNRNPK*		*GAPDH*	*HNRNPK*		*GAPDH*	*HNRNPK*		*GAPDH*	*HNRNPK*
**CSC 1.1**	DLD-1	0,263	0,385247	DLD-1	4,51	6,61				DLD-1	1,66	2,42			
	HT-29	0,0057	0,002867	HCT116	12,50	10,37				HCT116	19,30	14,94			
	SNU-C2B	2,24	3,280365	HT-29	23,65	12,07				HCT15	0,47	0,48			
	WiDr	0,605	0,596195	SW480	6,00	7,69				SNU-C2B	17,31	25,00			
	n. RNA	48,2968	34,38855	n. RNA	27,74	19,75				SW48	13,29	9,96			
										SW480	14,46	18,43			
										n. RNA	11,50	8,13			
**CSC 1.2**	DLD-1	0,30	0,56				DLD-1	15,60	28,72	DLD-1	1,63	3,00	HT-29	199	127
	HT-29	< 0.00649	< 0.004140				HT-29	10,58	6,75	HCT116	18,90	18,52	SW48	157	191
	SNU-C2B	2,57	4,74				SW480	20,59	33,22	HCT15	0,46	0,60	SW620	159	192
	WiDr	0,69	0,86				WiDr	9,74	11,99	SNU-C2B	16,96	30,99	WiDr	113	140
	n. RNA	55,48	49,65				n. RNA	11,50	10,29	SW48	13,01	12,35	n. RNA	24,66	22,07
										SW480	14,16	22,85			
										n. RNA	11,27	10,08			
**CSC 18**	DLD-1	0,039	0,052	DLD-1	73,88	78,34							HT-29	230	106
	HT-29	< 0.00084	< 0.000389	HCT116	205	123							SW48	182	160
	SNU-C2B	0,33	0,45	HT-29	387	143							SW620	184	161
	WiDr	0,090	0,081	SW480	98,16	91,24							WiDr	131	118
	n. RNA	7,21	4,67	n. RNA	454	234							n. RNA	28,53	18,49
**CSC 85**	DLD-1	0,57	0,54	DLD-1	2,94	2,72	DLD-1	16,72	15,50	DLD-1	3,93	3,64	HT-29	227	75,04
	HT-29	< 0.01215	< 0.004020	HCT116	8,13	4,27	HT-29	11,34	3,64	HCT116	45,58	22,49	SW48	179	113
	SNU-C2B	4,81	4,60	HT-29	15,39	4,97	SW480	22,07	17,92	HCT15	1,10	0,73	SW620	182	114
	WiDr	1,30	0,84	SW480	3,90	3,17	WiDr	10,44	6,47	SNU-C2B	40,90	37,63	WiDr	129	83
	n. RNA	104	48,22	n. RNA	18,05	8,13	n. RNA	12,33	5,56	SW48	31,38	14,99	n. RNA	28,16	13,08
										SW480	34,15	27,74			
										n. RNA	27,17	12,24			
**CSC CRO1**	DLD-1	0,348	0,30	DLD-1	16,12	13,87	DLD-1	31,79	27,36	DLD-1	3,22	2,77	HT-29	164	28,72
	HT-29	< 0.00747	< 0.002228	HCT116	44,65	21,76	HT-29	21,56	6,43	HCT116	37,35	17,11	SW48	129	43,23
	SNU-C2B	2,96	2,55	HT-29	84,49	25,35	SW480	41,95	31,64	HCT15	0,91	0,55	SW620	131	43,53
	WiDr	0,799	0,46	SW480	21,42	16,16	WiDr	19,84	11,42	SNU-C2B	33,51	28,64	WiDr	93,30	31,86
	n. RNA	63,87	26,73	n. RNA	99,09	41,47	n. RNA	23,44	9,81	SW48	25,72	11,41	n. RNA	20,31	5,01
										SW480	27,99	21,11			
										n. RNA	22,26	9,32			
**CSC CC1**	DLD-1	2,45	1,42	DLD-1	116	71,70	DLD-1	153	94,61				HT-29	767	164
	HT-29	< 0.05269	< 0.010553	HCT116	320	113	HT-29	104	22,22				SW48	606	246
	SNU-C2B	20,88	12,07	HT-29	606	131	SW480	201	109				SW620	615	248
	WiDr	5,63	2,19	SW480	154	84	WiDr	95,26	39,50				WiDr	438	182
	n. RNA	450	127	n. RNA	711	214	n. RNA	113	33,92				n. RNA	95,26	28,52
**CSC CC2**	DLD-1	78,73	76,58	DLD-1	134,723	96,5936									
	HT-29	< 1.69214	< 0.569929	HCT116	373,213	151,572									
	SNU-C2B	670	652	HT-29	706,162	176,541									
	WiDr	181	119	SW480	179,005	112,506									
	n. RNA	14450,7	6836	n. RNA	828,212	288,786									
**CSC CC5**	DLD-1	0,92	0,62	DLD-1	160,214	128,343				DLD-1	3,10	2,49	HT-29	400	111
	HT-29	< 0.01983	< 0.004625	HCT116	443,828	201,391				HCT116	36,01	15,36	SW48	316	167
	SNU-C2B	7,86	5,29	HT-29	839,773	234,567				HCT15	0,87	0,50	SW620	320	168
	WiDr	2,12	0,96	SW480	212,874	149,485				SNU-C2B	32,31	25,70	WiDr	228	123
	n. RNA	169	55,48	n. RNA	984,916	383,706				SW48	24,79	10,24	n. RNA	49,65	19,34
										SW480	26,98	18,95			
										n. RNA	21,46	8,36			

		16			17			18			19			20*	
		EMP2			LRIG1			OXGR1			LRP8			EDAR	
		%CSC expression			%CSC expression			%CSC expression			%CSC expression			%CSC expression	
	normal.	*GAPDH*	*HNRNPK*		*GAPDH*	*HNRNPK*		*GAPDH*	*HNRNPK*		*GAPDH*	*HNRNPK*		*GAPDH*	*HNRNPK*
**CSC 1.1**							HCT116<br>HT-29<br>LoVo<br>WiDr<br>n. RNA	0,29<br>0,75<br>0,45<br>1,61<br>121	0,25<br>0,40<br>0,83<br>1,67<br>90,94	Caco-2<br>HCT116<br>LoVo<br>SNU-C2B<br>n. RNA	111<br>128<br>61,14<br>87,06<br>6,16	72,37<br>111<br>114<br>133<br>4,65	Caco-2<br>HCT15<br>HT-29<br>WiDr<br>n. RNA	214<br>21<br>0,59<br>0,71<br>25,70	133<br>22<br>0,30<br>0,69<br>18,17
**CSC 1.2**	HCT116<br>LoVo<br>SNU-C2B<br>SW620<br>n. RNA	26,43<br>32,53<br>21,32<br>65,52<br>168	27,36<br>71,70<br>39,23<br>79,00<br>152	DLD-1<br>HCT116<br>HCT15<br>HT-29<br>n. RNA	0,0013<br>4,77<br>0,044<br>18,95<br>120	0,0024<br>4,97<br>0,057<br>12,16<br>108	HCT116<br>HT-29<br>LoVo<br>WiDr<br>n. RNA	0,10<br>0,27<br>0,16<br>0,58<br>43,53	0,11<br>0,17<br>0,36<br>0,72<br>39,23	Caco-2<br>HCT116<br>LoVo<br>SNU-C2B<br>n. RNA	307<br>356<br>169<br>241<br>17,08	239<br>368<br>376<br>441<br>15,39	Caco-2<br>HCT15<br>HT-29<br>WiDr<br>n. RNA	90,75<br>9,09<br>0,25<br>0,30<br>10,88	71,20<br>11,91<br>0,16<br>0,37<br>9,74
**CSC 18**							HCT116<br>HT-29<br>LoVo<br>WiDr<br>n. RNA	0,012<br>0,031<br>0,019<br>0,067<br>4,99	0,0090<br>0,014<br>0,030<br>0,060<br>3,26	Caco-2<br>HCT116<br>LoVo<br>SNU-C2B<br>n. RNA	46,33<br>53,59<br>25,53<br>36,35<br>2,57	24,23<br>37,24<br>38,02<br>44,60<br>1,56	Caco-2<br>HCT15<br>HT-29<br>WiDr<br>n. RNA	382<br>38,27<br>1,05<br>1,26<br>45,82	173<br>28,89<br>0,38<br>0,90<br>23,63
**CSC 85**							HCT116<br>HT-29<br>LoVo<br>WiDr<br>n. RNA	0,132<br>0,342<br>0,205<br>0,734<br>55,10	0,07<br>0,11<br>0,23<br>0,46<br>25,00	Caco-2<br>HCT116<br>LoVo<br>SNU-C2B<br>n. RNA	661<br>765<br>364<br>519<br>36,73	297<br>456<br>465<br>546<br>19,06	Caco-2<br>HCT15<br>HT-29<br>WiDr<br>n. RNA	1069<br>107<br>2,93<br>3,54<br>128	436<br>72,84<br>0,97<br>2,28<br>59,58
**CSC CRO1**	HCT116<br>LoVo<br>SNU-C2B<br>SW620<br>n. RNA	16,72<br>20,59<br>13,49<br>41,47<br>106	4,77<br>12,50<br>6,84<br>13,77<br>26,43				HCT116<br>HT-29<br>LoVo<br>WiDr<br>n. RNA	0,014<br>0,035<br>0,021<br>0,076<br>5,71	0,0039<br>0,0063<br>0,013<br>0,026<br>1,42	Caco-2<br>HCT116<br>LoVo<br>SNU-C2B<br>n. RNA	169<br>196<br>93,30<br>133<br>9,41	36,60<br>56,25<br>57,43<br>67,36<br>2,35	Caco-2<br>HCT15<br>HT-29<br>WiDr<br>n. RNA	76,04<br>7,61<br>0,21<br>0,25<br>9,12	27,89<br>4,67<br>0,06<br>0,15<br>3,82
**CSC CC1**	HCT116<br>LoVo<br>SNU-C2B<br>SW620<br>n. RNA	56,64<br>69,74<br>45,69<br>140<br>361	19,75<br>51,76<br>28,32<br>57,04<br>109				HCT116<br>HT-29<br>LoVo<br>WiDr<br>n. RNA	1,30482<br>3,39605<br>2,03335<br>7,27958<br>546,416	0,35945<br>0,57589<br>1,18415<br>2,38478<br>130,134	Caco-2<br>HCT116<br>LoVo<br>SNU-C2B<br>n. RNA	285<br>329<br>157<br>223<br>15,82	74,74<br>115<br>117<br>138<br>4,80	Caco-2<br>HCT15<br>HT-29<br>WiDr<br>n. RNA	4022<br>403<br>11,03<br>13,30<br>482	1063<br>178<br>2,37<br>5,56<br>145
**CSC CC2**				DLD-1<br>HCT116<br>HCT15<br>HT-29<br>n. RNA	0,0082<br>29,73<br>0,27<br>118<br>746	0,0080<br>16,32<br>0,19<br>40<br>354	HCT116<br>HT-29<br>LoVo<br>WiDr<br>n. RNA	70,22<br>183<br>109<br>392<br>29407	43,23<br>69,26<br>142<br>287<br>15650				Caco-2<br>HCT15<br>HT-29<br>WiDr<br>n. RNA	7301<br>731<br>20,03<br>24,15<br>875	3006<br>503<br>6,70<br>15,71<br>411
**CSC CC5**				DLD-1<br>HCT116<br>HCT15<br>HT-29<br>n. RNA	0,0035<br>12,76<br>0,12<br>50,70<br>320	0,0028<br>5,79<br>0,07<br>14,16<br>126	HCT116<br>HT-29<br>LoVo<br>WiDr<br>n. RNA	0,094<br>0,244<br>0,146<br>0,523<br>39,23	0,04<br>0,06<br>0,12<br>0,24<br>12,94						

	21*		
	MME		
	%CSC expression		
	GAPDH	*HNRNPK*	*normalization*
Caco-2	19,34	11,99	**CSC 1.1**
DLD-1	0,86	1,25	
LoVo	0,17	0,29	
SNU-C2B	0,96	1,38	
SW480	3,15	4,01	
n. RNA	2425	1715	
Caco-2	0,64	0,50	**CSC 1.2**
DLD-1	0,028	0,052	
LoVo	0,0055	0,012	
SNU-C2B	0,032	0,058	
SW480	0,10	0,17	
n. RNA	80,11	71,70	
			**CSC 18**
			**CSC 85**
			**CSC CRO1**
			**CSC CC1**
			**CSC CC2**
DLD-1	2,30	1,85	**CSC CC5**
LoVo	0,45	0,43	
SNU-C2B	2,56	2,03	
SW480	8,42	5,91	
n. RNA	6489	2528	
